# Polyphenol‐Based Functional Materials: Structural Insights, Composite Strategies, and Biomedical Applications

**DOI:** 10.1002/advs.202508924

**Published:** 2025-08-27

**Authors:** Songwen Xue, Wen Tan, Shuifang Mao, Haibo Pan, Xingqian Ye, Natthawuddhi Donlao, Jinhu Tian

**Affiliations:** ^1^ College of Biosystems Engineering and Food Science Zhejiang Key Laboratory of Agri‐Food Resources and High‐value Utilization Zhejiang University Hangzhou 310058 China; ^2^ Zhejiang University Zhongyuan Institute Zhengzhou 450000 China; ^3^ Unit of Innovative Food Packaging and Biomaterials School of Agro‐Industry Mae Fah Luang University Chiang Rai 57100 Thailand; ^4^ Wuxi Xishan Modern Agriculture Joint Research Center Zhejiang University Wuxi 214117 China

**Keywords:** biomedical applications, functional materials, polyphenol, self‐assembly

## Abstract

Polyphenols hold significant promise in pharmaceutical, biotechnology, and food‐related applications owing to their potent free radical scavenging, antimicrobial, antitumor, and other properties. The unique chemical architecture, featuring multiple phenolic hydroxyl groups and aromatic ring systems‐confers a high capacity for both non‐covalent (e.g., hydrogen bonding, π–π stacking, metal ion coordination) and covalent interactions (e.g., Michael addition, Schiff base formation). These versatile interaction modes underpin the rational design and engineering of advanced composite materials with tailored functionalities. Recent advances in nanotechnology and materials science have catalyzed the integration of polyphenols with broad biomaterials, including metals, polysaccharides, and proteins, to enhance their biocompatibility, mechanical properties, and therapeutic efficacy. This review systematically explores the sources, structures, and physiological activities of polyphenols, elucidating their interaction mechanisms with different materials. Emphasis focuses on the design of polyphenol‐based nanomaterials, bioactive scaffolds, and smart drug delivery platforms capable of modulating local microenvironments and orchestrating cellular responses for precision therapeutic interventions. The translational potential of these functional materials in regenerative and precision medicine is also critically examined, alongside key challenges such as stability, responsiveness, and the fine‐tuning of release kinetics.

## Graphical abstract

This schematic representation illustrates the interaction mechanisms between polyphenolic compounds and various materials (metals, proteins, polysaccharides, alkaloids, etc.). And explains the potential clinical application value of these materials (nanoparticles, coatings, films, capsules, and hydrogels constructed using polyphenols) in the fields of regenerative medicine and precision medicine through their intelligent functional characteristics (photothermal effects, pH responsiveness, etc.). Created with BioRender.com.

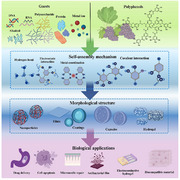

## 1 Introduction

Polyphenols are a large class of secondary metabolites widely found in plants, algae, and certain microorganisms, exhibiting diverse structures and biological activities. They are extensively distributed in the roots, stems, leaves, and fruits of higher plants. Polyphenols typically consist of one or more phenolic groups, with each benzene ring bearing one or more active hydroxyl groups. Due to their unique and complex structures, they exhibit various functional properties such as free radical scavenging, antibacterial activity, antitumor activity, pH responsiveness, and photothermal performance.^[^ ^1^ ^]^ The presence of phenolic hydroxyl groups enables polyphenols to interact more readily with other substances through non‐covalent and covalent bonds, thereby altering their properties and forming structures such as nanoparticles, films, coatings, capsules, and hydrogels.^[^ ^2^ ^]^ Through interactions such as hydrogen bonding, hydrophobic interactions, π–π stacking, MPN coordination, Michael addition, Schiff base reaction/nucleophilic addition reactions, polyphenols have become ideal candidates for developing innovative carrier systems and functional materials in fields of pharmacy, biotechnology, and food science.^[^ ^3^ ^]^

Over the past few years, with the rapid advancement of nanotechnology and materials science, the development of composite strategies integrating polyphenols with various materials has emerged as a cutting‐edge field in biomaterials and drug delivery.^[^ ^4^ ^]^ Incorporating polyphenol functionalization into various materials not only enhances the structural diversity of composite materials and improves their inherent properties but also endows the materials with unique functional characteristics. For instance, incorporating polyphenols into tissue materials could enhance their biocompatibility, strengthen antioxidant capacity, and improve adhesion. The addition of polyphenols reduces local inflammation and oxidative damage at wound sites, offering new therapeutic strategies for bone defects and skin wound healing.^[^ ^5^ ^]^ When applied to textiles, polyphenols form a polyphenol coating on the surface of the fabric through oxidative cross‐linking, imparting hydrophobicity, flame retardancy, and antibacterial properties to the fabric.^[^ ^6^ ^]^ In addition, the catechin and gallic acid (GA) units in the polyphenol structure give it powerful antioxidant properties, which could be added to sunscreen cosmetics to help effectively neutralize excess free radicals in the skin and provide protection against UV radiation.^[^ ^7^ ^]^ Based on their photothermal properties, polyphenols could also influence cell behavior in a cell‐material contact‐independent manner, serving as a multifunctional polyphenol platform for regulating cell biology.^[^ ^8^ ^]^ Collectively, polyphenols with multiple functional properties have been extensively studied and applied across various systems (e.g., tissue engineering materials,^[^ ^9^ ^]^ scaffolds,^[^ ^10^ ^]^ synthetic biology,^[^ ^8^ ^]^ and delivery carriers^[^ ^9^, ^11^ ^]^).

In recent years, reviews comprehensively summarizing the formation of various morphological structures and multiple application pathways of polyphenol–metal combinations have accounted for a significant portion of polyphenol research.^[^ ^12^ ^]^ However, there is currently no systematic discussion of the process and mechanisms by which polyphenols combine with different materials to form different morphological structures. This paper mainly reviews the combination of polyphenols with different materials, such as metals, proteins, polysaccharides, nucleic acids, and alkaloids, so as to change the properties of the materials themselves and improve the overall functional properties of the complex materials. The sources, structures and their physiological activities of polyphenols are systematically described, and their mechanisms under different structures are also discussed. Then, we focus on reviewing the synergistic effects between polyphenols and different materials, including nanomaterials, polyphenol scaffolds, and smart drug delivery systems, as well as how these composite strategies improve the local microenvironment and modulate cellular behaviors to achieve precision therapy. Finally, the potential future applications of polyphenol‐based functional materials in clinical translation and precision medicine are also involved. This review will provide important and further insights into the nature, functions, and applications of polyphenols.

## 2 Polyphenols

Polyphenols are predominantly present in plants. Attributing to their unique properties, plant polyphenols could serve as a natural defense barrier, enabling plants to resist pest and pathogen invasions, while inhibiting the proliferation of pathogens. Polyphenols also play pivotal roles in various plant processes, including signal transduction, metabolic regulation, and protection against DNA damage.^[^ ^13^ ^]^ This section will primarily introduce the sources, classification, and functions of plant polyphenols.

### 2.1 Polyphenol Sources

**Figure 1:**
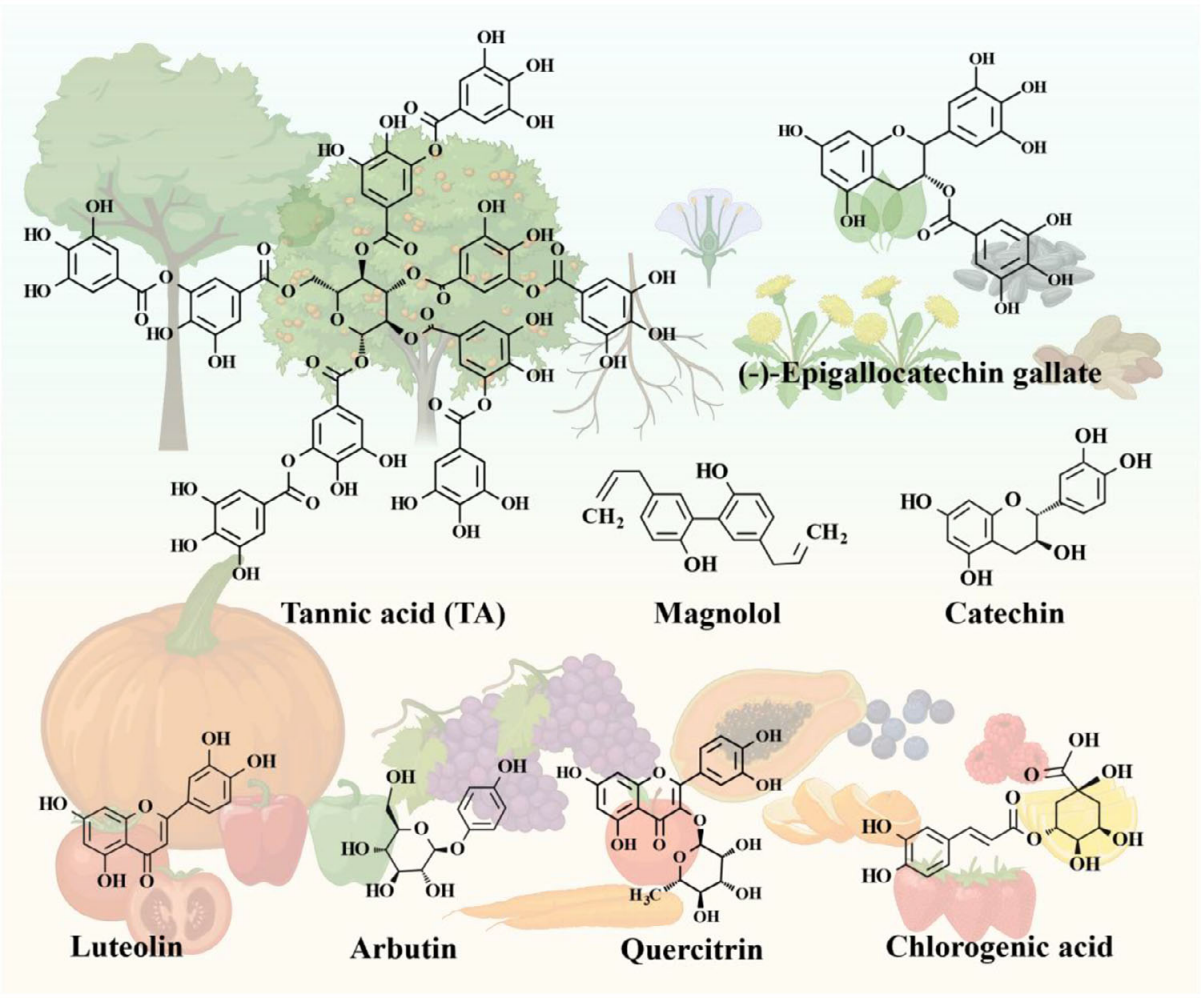
Representative natural polyphenols.Created with BioRender.com.

Polyphenols are predominantly present in roots, barks, leaves, and fruits of higher plants. Certain plants with well‐developed root systems exhibit a particularly high concentration of diverse polyphenols (**Figure** **1**). *Glycyrrhiza* species are notably rich in bioactive secondary metabolites, and their roots have historically been utilized for the treatment of various ailments.^[^ ^14^ ^]^ The stems of *Rubus idaeus* incorporate polyphenolic compounds, and the accumulation of polyphenols within the stems varies among different parts, and the content of polyphenols in the stems fluctuates with the seasons.^[^ ^15^ ^]^ Some evergreen plants, such as tea plants,^[^ ^16^ ^]^ olive trees,^[^ ^17^ ^]^ mulberry trees,^[^ ^18^ ^]^ and *Pistacia lentiscus* ^[^ ^19^ ^]^ likewise contain abundant polyphenols in their leaves. *Pistacia lentiscus* has polyphenols in its fruits, leaves, and roots, with the highest total polyphenol content found in the leaves, mainly consisting of flavonoids and tannins.^[^ ^20^ ^]^ The vast majority of plant fruits also contain various polyphenolic compounds, such as berries of blueberries,^[^ ^21^ ^]^ strawberries,^[^ ^22^ ^]^ and cranberries,^[^ ^23^ ^]^ and citrus fruits like tangerines, oranges, and lemons^[^ ^24^ ^]^ (Figure 1). A previous study had utilized LC‐ESI‐QTOF‐MS/MS and HPLC‐PDA techniques to qualitatively and quantitatively analyze the content and types of polyphenolic compounds from 20 types of fruit peels, and the results supported the notion that fruit peels are potential sources of phenolic compounds.^[^ ^25^ ^]^

### 2.2 Polyphenol Classifications by Chemical Structures

The chemical structures of polyphenols are highly complex and diverse, typically consisting of one or more phenolic moieties, each benzene ring bearing one or more active hydroxyl groups. This intricate structural arrangement is fundamental to their remarkable biological activities, including antioxidant, anti‐inflammatory, antitumor, cardioprotective, and neuroprotective effects.^[^ ^26^ ^]^ Polyphenols are generally classified according to the complexity of their carbon skeleton into simple phenolic acids, flavonoids, lignans, and tannins.

#### 2.2.1 Flavonoids

**Figure 2:**
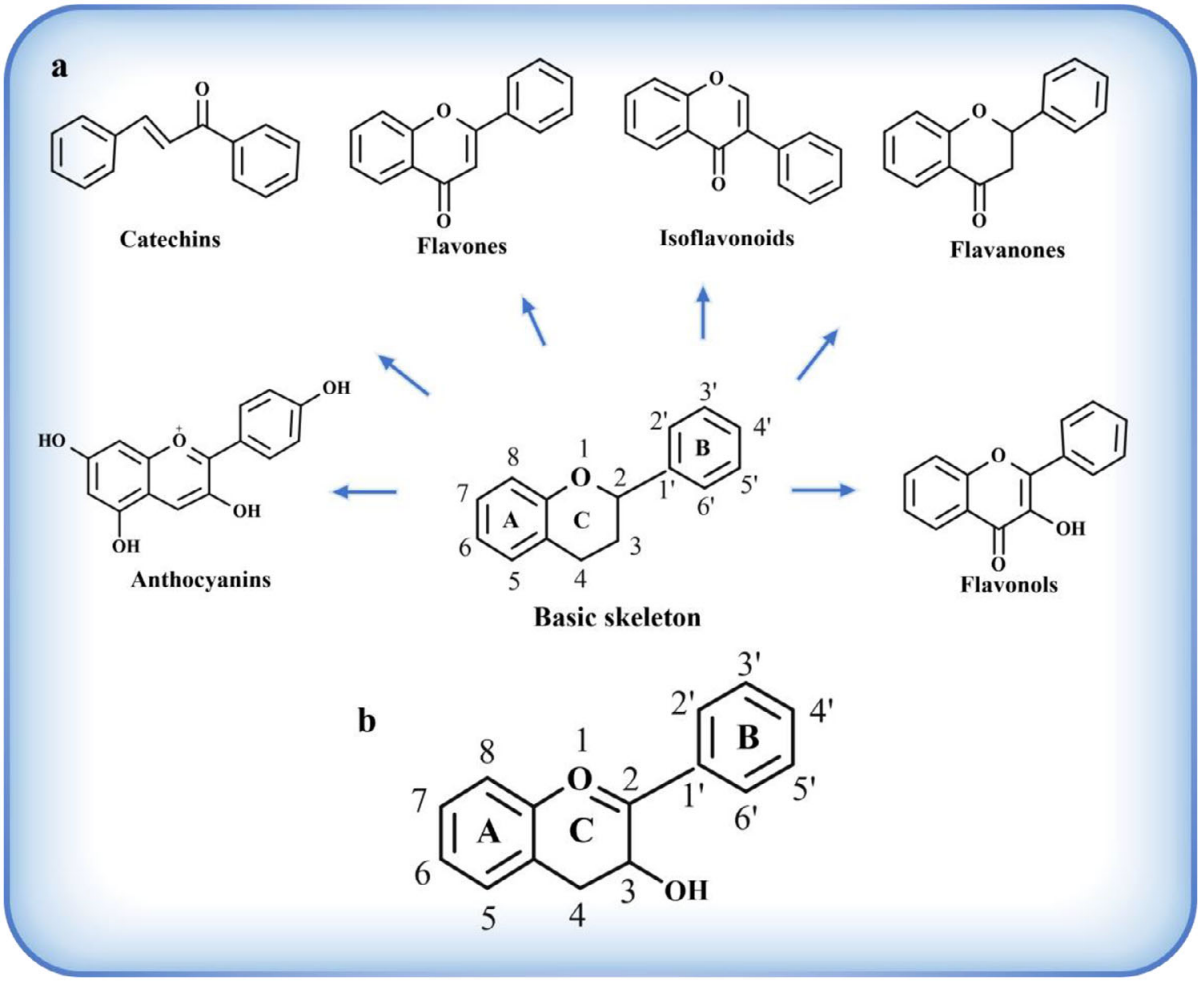
a) Basic skeleton structure of flavonoids and their classes. b) Structure of Flavan‐3‐ol and its nomenclature.

Flavonoids represent the most extensively studied subclass of polyphenols, characterized by a C6‐C3‐C6 carbon backbone.^[^ ^27^ ^]^ The flavonoids are the general term for a series of compounds consisting of two benzene rings interconnected by three carbon atoms, which could be further classified into different subgroups based on the carbon of the C‐ring, to which the B‐ring is attached and the degree of unsaturation and oxidation of the C‐ring, such as flavones, flavanones, flavanols, catechins, anthocyanins, chalcones and so on^[^ ^28^ ^]^ (**Figure** **2**a). Flavones are the simplest structural flavonoids, with a molecular structure containing only two benzene rings and the pyran ring. Its anti‐inflammatory properties are thought to be more potent than those of other flavonoids, which might be related to the number and position of phenolic hydroxyl groups on the A and B rings.^[^ ^29^ ^]^ Flavonols are flavonoids with a ketone group and are components of proanthocyanidins. They differ from flavonoids in that they have only non‐phenolic hydroxyl group at the 3 position of the C ring, and the hydroxyl group at the 3 position is easily glycosylated.^[^ ^27^, ^30^ ^]^ Flavonoids are also known as dihydroflavonoids, in which the C ring is saturated, there is no substituent at position 3, and they do not contain double bonds at positions 2 and 3. In addition, ring A or ring B contains O‐ (hydroxyl, methoxy, methylenedioxy, etc.).^[^ ^31^ ^]^

#### 2.2.2 Tannins

In addition to flavonoids, tannins are another important subclass of polyphenolic compounds with a wide range of biological activities, such as antioxidant, antimicrobial, and anti‐inflammatory activities.^[^ ^32^ ^]^ Tannins are usually classified into hydrolysable tannins, condensed tannins, and phlorotannins based on their structural characteristics.^[^ ^33^ ^]^ Hydrolyzable tannins are formed by esterification of glucose with gallic acid/ellagic acid, which, upon hydrolysis, produce gallic acid, ellagic acid, and sugar. These compounds could undergo hydrolysis under acidic conditions, releasing free phenolic acids that exhibit potent radical scavenging abilities.^[^ ^34^ ^]^ Based on compositional classification, hydrolysable tannins could be divided into ellagitannins and gallotannins. However, based on structural characteristics, hydrolysable tannins could be classified into at least three types: simple gallic acid derivatives, gallotannins, and ellagitannins. Simple gallic acid derivatives are characterized by containing ≤ 5 galloyl groups and are often esterified with quinic acid or glucose. Gallotannins are characterized by a core of glucose or quinic acid, a hydroxyl group that is pentacosanoylated, and contain ≥ 6 dicarbonyl groups. The structure of ellagitannins is characterized by the formation of galloyl groups attached to the glucose chain mainly by “de‐side bonding” (ester bonding between multiple aromatic units). As shown in Figure 2b, the core structure of condensed tannins consists of flavan‐3‐ol monomers polymerized by C─C bonds to form repeating units, each containing three rings, A, B, and C.^[^ ^35^ ^]^ The different structures of the A and B rings result in different structures of the repeating units in the tannins. the A ring is usually present as resorcinol and phloroglucinol, whereas the B ring is present as catechol or pyrocatechol.^[^ ^36^ ^]^ Connected by carbon–carbon bonds, it is not easy to be hydrolyzed and shows strong stability and long‐lasting biological activity prolonged.^[^ ^37^ ^]^ Phlorotannins, predominantly found in marine algae, are formed by the polymerization of phloroglucinol units and are distinguished by their high antioxidant capacity and metal‐chelating properties.^[^ ^38^ ^]^ They are formed from chloroglucosanol units (≥2) linked by C─O─C or C─C bonds, and are divided into three classes based on the coupling relationship between subunits: fucoidan (C─C), chloroethanol (C─O─C), and fucoidan chloroethanol (C─C and C─O─C). Additional hydroxyl groups in the molecule, bonds between monomers, and more chloroglucitol substituents (3 to 7 substituents) result in structural changes.

#### 2.2.3 Phenolic Acids and Lignans

Phenolic acids and lignans are also important components of polyphenolic compounds. Phenolic acids are compounds containing hydroxyl and carboxyl groups in a benzene ring. Based on differences in their carbon skeleton structures, phenolic acids could be classified into four types: benzoic acid‐type, phenylacetic acid‐type, cinnamic acid‐type, and phenolic acid derivatives. Common phenolic acids include protocatechuic acid, gallic acid, ferulic acid, caffeic acid, and salvianic acid, etc. Chlorogenic acid belongs to the class of phenolic acid derivatives, with a chemical structure containing one caffeic acid and one quinic acid, which are linked via a ─COO─ group to form a structurally stable ester. Chlorogenic acid possesses rich biological activity due to its unique phenolic hydroxyl structure and ester bond activity. Researchers have reported that chlorogenic acid primarily banded to dietary polysaccharides via non‐covalent bonds, exhibiting synergistic antioxidant effects. During simulated gastric and intestinal digestion processes, the free radical scavenging rate of digestive fluids increases with prolonged time.^[^ ^39^ ^]^ Gallic acid, also known as 3,4,5‐trihydroxybenzoic acid, is composed of a benzoic acid skeleton. It is widely distributed in various fruits, tea leaves, and medicinal plants. Due to the presence of three hydroxyl groups substituted on the benzene ring, gallic acid exhibits extremely high polarity, enabling it to effectively donate electrons or hydrogen atoms to scavenge free radicals, thereby demonstrating outstanding antioxidant properties.

Over the past few years, gallic acid has been found to possess significant antibacterial properties, capable of disrupting bacterial cell wall structures.^[^ ^40^ ^]^ Lignans are a class of complex polyphenolic bioactive plant chemicals and are a type of phytoestrogen. They contain multiple phenolic hydroxyl and methoxy functional groups and are composed of two molecules of phenylpropane (C6‐C3) forming the basic carbon skeleton.^[^ ^41^ ^]^ Lignans exhibit various bioactivities similar to estrogen, regulating hormone‐related diseases.^[^ ^42^ ^]^ Additionally, they possess free radical scavenging capabilities, inhibiting reactive oxygen species (ROS) production and modulating the activity of proteins associated with the cell cycle and apoptosis. Recent studies have indicated that long‐term consumption of lignans might reduce the risk of coronary heart disease.^[^ ^43^ ^]^

### 2.3 Polyphenol Physiological Activities

Polyphenols, a class of naturally occurring compounds characterized by diverse structures and rich functionalities, exhibit a wide range of significant biological activities. These activities include antioxidant,^[^ ^44^ ^]^ anti‐inflammatory,^[^ ^45^ ^]^ antibacterial,^[^ ^46^ ^]^ and antitumor effects,^[^ ^47^ ^]^ as well as the regulation of immune responses^[^ ^48^ ^]^ and the protection of the cardiovascular^[^ ^49^ ^]^ and nervous systems.^[^ ^50^ ^]^ The biological activities of polyphenols are primarily attributed to their abundant phenolic hydroxyl groups, which enable them to scavenge ROS^[^ ^51^ ^]^ and reactive nitrogen species,^[^ ^52^ ^]^ chelate metal ions, inhibit oxidative chain reactions, and modulate various cellular signaling pathways.

#### 2.3.1 Antioxidant and Anti‐Inflammatory Properties

**Figure 3:**
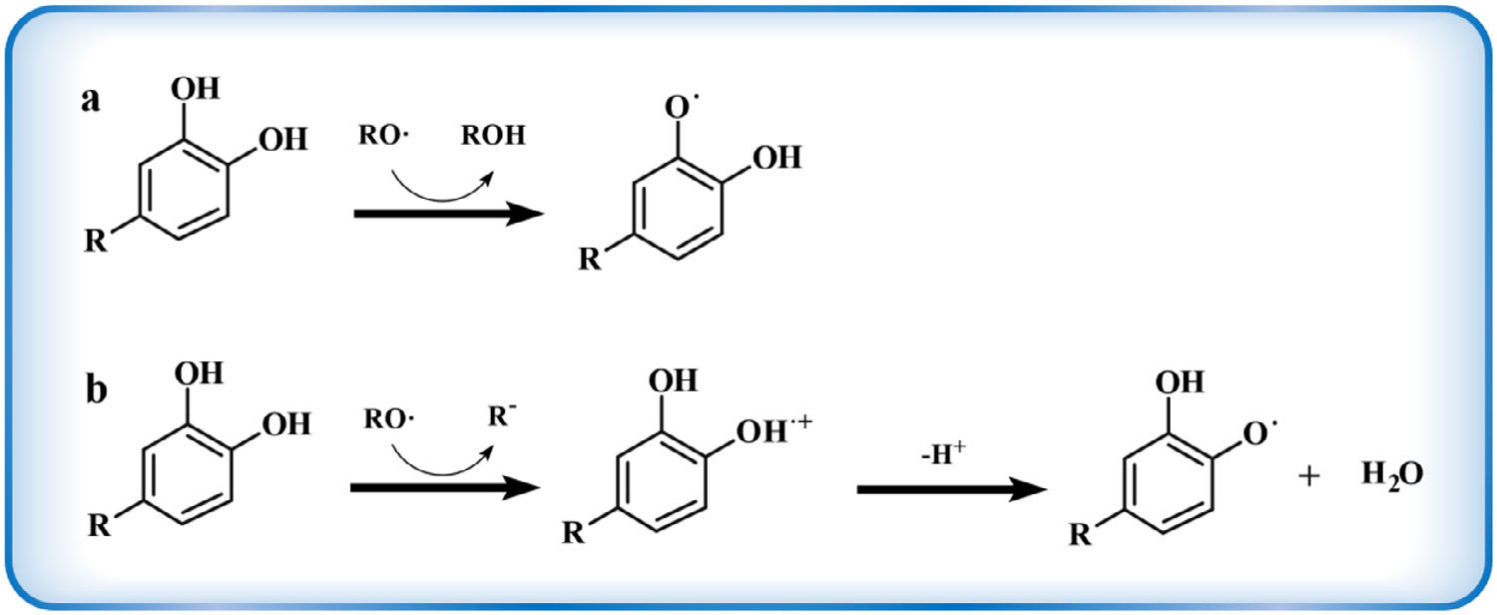
A schematic diagram illustrating the free radical scavenging mechanism of polyphenols. a) Hydrogen supply. b) Electron transfer.

Numerous studies have demonstrated that polyphenols possess a wide range of biological activities, including immunomodulatory, neuroprotective and therapeutic, and preventive effects on cardiovascular diseases. However, the most notable role of polyphenols as effective antioxidants is their ability to scavenge free radicals both in vivo and in vitro.^[^ ^53^ ^]^ The primary mechanisms by which polyphenols scavenge free radicals include hydrogen donor and electron transfer.^[^ ^13^, ^54^ ^]^ Polyphenols could convert free radicals into stable compounds by transferring their own hydrogen atoms to free radical‐containing compounds (**Figure** **3**a).^[^ ^10^, ^54^ ^]^ Polyphenols also donate an electron to free radicals, leading to free radical recombination, ultimately forming stable compounds and water (Figure 3b).^[^ ^10^, ^54^ ^]^ Additionally, both the aforementioned mechanisms could occur simultaneously to eliminate free radicals. Furthermore, polyphenols could also exhibit antioxidant effects through metal chelation. By chelating with metal ions (Fe^2^⁺, Cu^2^⁺), they prevent these ions from participating in processes such as the Fenton reaction that generate ROS, thereby reducing free radical production at its source.^[^ ^55^ ^]^

A growing body of evidences have demonstrated that polyphenolic compounds exert systemic anti‐inflammatory effects by modulating multiple intracellular signaling pathways involved in oxidative stress and immune activation. For instance, Catalina et al.^[^ ^56^ ^]^ showed that quercetin alleviated cholesterol‐induced inflammation by suppressing the activation of key apoptotic regulators caspase‐3 and ‐9, inhibiting cytochrome c release from mitochondria, and blocking the activation of the NF‐κB pathway, thereby mitigating inflammation at the cellular level. Similarly, Kelly et al.^[^ ^57^ ^]^ systematically assessed 23 studies on anthocyanins and reported that these flavonoids attenuated hepatic steatosis and oxidative stress by downregulating pro‐inflammatory cytokines (e.g., TNF‐α, IL‐6, IL‐1β) while enhancing the activities of antioxidant enzymes such as superoxide dismutase (SOD), glutathione (GSH), glutathione peroxidase (GPx), and catalase (CAT). These effects are likely mediated through activation of the Nrf2 signaling axis and suppression of NF‐κB‐mediated transcriptional responses. Yu et al.^[^ ^58^ ^]^ further verified that treatment with epigallocatechin gallate (EGCG) effectively reduced the expression of pro‐inflammatory cytokines (e.g., IL‐6, MCP‐1, and TNF‐α) in the intestine, inhibited the infiltration of CD3⁺ T cells and CD68⁺ macrophages, and improved the immune microenvironment in the colitis model. Those findings underscored the therapeutic potential of polyphenols in treating metabolic disorders, inflammatory bowel disease.

#### 2.3.2 Antimicrobial Activity

In addition to their well‐documented antioxidant and anti‐inflammatory properties, polyphenols have demonstrated potent antimicrobial activity. For instance, isoflavones (genistein and its derivatives) exhibited broad‐spectrum antibacterial effects against eight bacterial strains, including methicillin‐resistant *Staphylococcus aureus* (MRSA), with minimum inhibitory concentration (MIC) and minimum bactericidal concentration (MBC) values ranging from 8 to128 µg mL^−1^.^[^ ^59^ ^]^ Likewise, quercetin and kaempferol also exhibited strong antimicrobial activity against *Staphylococcus aureus* and MRSA, with MIC values as low as 1.95 and 7.8 µg mL^−1^, respectively.^[^ ^60^ ^]^ The antimicrobial mechanisms of polyphenols are relatively well elucidated. These compounds could disrupt the integrity of bacterial cell walls and membrane permeability, induce intracellular oxidative stress, inhibit DNA replication and protein synthesis, interfere with metabolic regulation, and suppress key enzymatic activities.^[^ ^61^ ^]^ Furthermore, polyphenol‐based nanocomposites have shown promise in preventing biofilm formation. For example, curcumin‐containing bio‐nanocomposites effectively inhibited the adhesion of *Streptococcus mutans* to tooth surfaces by blocking Sortase A activities, thereby reducing biofilm development.^[^ ^62^ ^]^

#### 2.3.3 Anticancer Activity

During recent years, polyphenolic compounds have garnered significant attention for their potential anticancer properties against various human malignancies. The anticancer mechanisms mediated by polyphenols primarily involve: 1) induction of oxidative stress and mitochondrial dysfunction via ROS, 2) activation of apoptotic and necrotic signaling pathways, 3) regulation of autophagic processes, and 4) cell cycle arrest, etc. Distinct classes of polyphenols exhibit diverse structural features, enabling differential anticancer effects across tumor types. For instance, quercetin has been shown to induce apoptosis in BT‐474 breast cancer cells by downregulating HER2 expression and inhibiting the phosphorylation of JAK1 and STAT3.^[^ ^63^ ^]^ Luteolin triggers iron metabolism, imbalance of mitochondrial membrane potential, production of ROS, and abnormal GSH depletion in clear‐cell renal cell carcinoma by over‐regulation of HO‐1 expression and activation of labile iron pool, which exerts antitumor effects.^[^ ^64^ ^]^ Likewise, rutin inhibited the proliferation and migration of pancreatic cancer cells by suppressing the transcription of Bcl‐2 and inducing apoptosis by upregulating the expression of miR‐877‐3p.^[^ ^65^ ^]^ Despite these promising effects, the clinical efficacy of polyphenols remains limited due to their inherently low bioavailability in the gastrointestinal tract, rapid hepatic metabolism, and extensive renal and biliary excretion.^[^ ^66^ ^]^ Therefore, enhancing the stability, mucosal adhesion, and tumor targeting capability of polyphenol‐based therapeutics is of great importance in cancer treatment.^[^ ^67^ ^]^

## 3 Interaction Mechanism of Polyphenols

Phenolic compounds contain a large number of dihydroxyphenyl (catechol) or trihydroxyphenyl (pyrogallol) groups, which have high π‐electron content and aromaticity and could provide effective negative binding sites for cations. Their biological activities depend not only on their own structure, but also closely related to their interactions with proteins, lipids, metal ions, and other biomolecules. In the following, an in‐depth introduction will be provided on the mechanisms of these non‐covalent (hydrogen bonding, electrostatic, hydrophobic, π–π stacking, metal coordination, and boronate–phenolic network) and covalent (oxidative coupling reactions, nucleophilic addition reactions, Schiff base reactions, and free radical coupling reactions) interactions.

### 3.1 Non‐Covalent Interactions

#### 3.1.1 Hydrogen Bond Interaction

**Figure 4:**
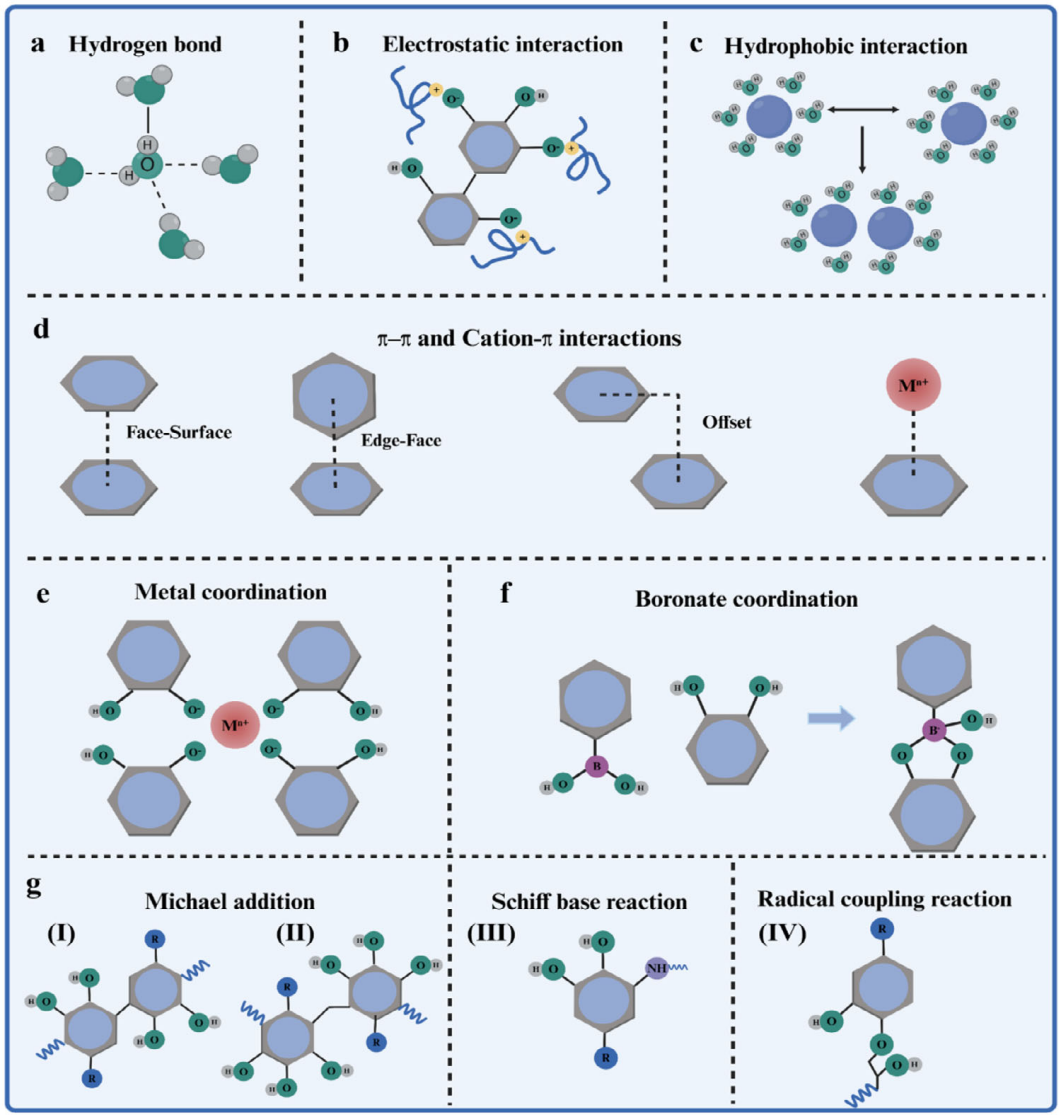
Chemical interactions between polyphenols and different materials. a–d) Noncovalent interactions of polyphenols, hydrophobic interaction (a), electrostatic interaction (b), hydrophobic interaction (c), and π–π/Cation–π interactions (d),^[^ ^72^ ^]^ respectively. Copyright 2024, Wiley‐VCH GmbH. e,f) Dynamic covalent bonds of polyphenols, metal–polyphenol interactions (e), and phenylboronic acid–polyphenol interactions (f), respectively. g) Covalent interaction. Created with BioRender.com.

Hydrogen bonding is a common intermolecular force, as shown in **Figure** **4**a. Polyphenols rich in hydroxyl groups (─OH) (e.g., TA, EGCG, anthocyanins) could serve as hydrogen bond donors and acceptors to form noncovalent complexes with polar molecules.^[^ ^68^ ^]^ When the pH is below the pKa value, most of the hydroxyl groups in polyphenols are protonated and form extensive intermolecular hydrogen bonds with other substances. The phenolic hydroxyl groups of polyphenols form hydrogen bonding networks with polar amino acid residues of proteins (e.g., glutamic acid, lysine), and cross‐links with polysaccharide compounds, such as cellulose and starch, through hydrogen bonding. For example, by incorporating TA into hexanoyl glycol chitosan, the pyrogallol/hydroxyl groups of TA could hydrogen‐bond with the amine and hydroxyl groups of hexanoyl glycol chitosan, which enhanced the mechanical strength, self‐healing ability, stability, and biodegradability of the hexanoyl glycol chitosan thermosensitive hydrogel.^[^ ^69^ ^]^ Hence, hydrogen bonding regulated the thermodynamic stability of polyphenol complexes through directed donor–acceptor interactions. The hydrogen bonding interaction between tannins and the ─OH and ─NH_2_ groups in chitosan played a crucial role in modifying the surface properties and triboelectric charging behavior of the chitosan films.^[^ ^70^ ^]^ In addition, the catechol moiety produces intermolecular hydrogen bonding interactions with polyvinyl alcohol and polyacrylic acid. Under acidic conditions, a small sustained release of EGCG could reduce the inflammatory response, promote vascular maturation, and collagen deposition due to the re‐formation of intermolecular hydrogen bonding and the reduction of swelling of the fibrous structure.^[^ ^71^ ^]^

#### 3.1.2 Electrostatic Interaction

When the pH value exceeds the pKa of polyphenols, the phenolic hydroxyl group could be partially ionized, resulting in a large number of negatively charged groups, as shown in Figure 4b.^[^ ^10^ ^]^ Normally, the negative charges generated by the dissociation of polyphenols form complexes with positively charged biomolecules or material passages through a charge complementarity mechanism (electrostatic interaction), which affects their biological activity and stability. For example, in the assembly of tannic acids (TA) with quaternary ammonium salts (e.g., benzalkonium chloride), the phenolic hydroxyl group of the TA molecule is partially deprotonated and negatively charged under alkaline conditions, and is combined by electrostatic attraction to form a stable TA‐benzalkonium chloride complex with the quaternary ammonium salts with positively charged nitrogen atoms.^[^ ^73^ ^]^

#### 3.1.3 Hydrophobic Interaction

The nonpolar groups of polyphenols (aromatic rings, alkyl chains, etc.) are embedded in the nonpolar regions of biomolecules (proteins, nucleic acids, etc.) through hydrophobic interactions, driving the formation of complexes and the regulation of functional properties, as shown in Figure 4c. Researchers used thermodynamic analysis and fluorescence spectroscopy to analyze the main interaction between rice protein and polyphenols, which was a hydrophobic interaction, and indicated that the hydrophobic portions of the GA, TA benzene ring structures were embedded into the hydrophobic pockets or hydrophobic regions on the surface of rice protein, forming a complex. This process was temperature‐regulated, manifested as dynamic changes in the binding sites. Although the process was endothermic (∆H > 0), the increase in entropy (∆S > 0) dominated the change in free energy (∆G < 0), revealed the thermodynamic essence of hydrophobic interactions in polyphenol–protein binding: by releasing ordered water molecules, they increase the disorder of the system, thereby driving the formation of the complex.^[^ ^74^ ^]^ Besides, previous research has also shown that hydrophobic interactions had a crucial role in the binding process of proteins and polyphenols, leading to the formation of stable complexes. This was due to the fact that polyphenol molecules containing hydrophobic groups could enter the hydrophobic pocket of proteins through hydrophobic reactions. As shown by fluorescence spectroscopy analysis, it was evident that the binding between the protein and polyphenol was dominated by static quenching, indicating that the complexes were formed through hydrophobic interactions.^[^ ^75^ ^]^

#### 3.1.4 π–π and Cation–π interactions

Mutual attraction (π–π interactions) resulting from the interaction of π electron clouds in the aromatic benzene rings of polyphenols is also widespread in phenol‐containing materials.^[^ ^76^ ^]^ Such interactions are noncovalent and typically occur between two or more aromatic ring molecules (Figure 4d). For example, the catechol moiety of dopamine and the benzene/quinone ring structure of Aniline Tetramer enhanced adhesion strength through π–π stacking.^[^ ^77^ ^]^ The delocalized π‐electron‐rich graphene oxide interacts with polyphenols through π–π stacking, inducing the formation of an intelligent hydrogel with microwave absorption properties. Moreover, the study demonstrated that temperature could modulate the dynamic interplay between π–π stacking and hydrogen bonding.^[^ ^78^ ^]^ In addition to the π–π interaction, polyphenols could form cation–π interactions with a variety of cations, substances containing positive charges. Additionally, the interactions between polyphenols and cation–π increased the superhydrophilicity of the coating. The binding of oxygen atoms in the hydroxyl group to the π system led to electronic delocalization of the lone electron pair of oxygen, which improved the negative charge density of the phenyl ring(Figure 4d).^[^ ^79^ ^]^

### 3.2 Metal–Phenolic Networks

Metal coordination represents a dynamic interaction that bridges covalent and noncovalent bonding, characterized by its reversible nature and unique coordination properties.^[^ ^80^ ^]^ The phenolic hydroxyl, carboxyl, and nitrogen groups in polyphenols could form dynamic coordination bonds with metal ions (Fe, Zn, Cu, etc.), as shown in Figure 4e.^[^ ^81^ ^]^ These metal ions are chelated within the polyphenol framework by interacting with electron‐donating groups, creating multiple coordination sites that significantly enhance the structural stability of the resulting complexes. The researchers directly assembled metal–polyphenol networks (MPNs) nanoparticles without templates or seeds by modulating pH and precursor concentration. And the results showed that the coordination cross‐linking of polyphenols with metal ions (e.g., Fe─O vibrational peaks) endowed the MPNs nanoparticles with high stability and biocompatibility in physiological environments, and the drug release could be achieved by pH modulation. In addition to amplifying the stability of the material, the MPNs confer self‐healing ability, dynamic response properties enabling the material to adapt to external stimuli (e.g., mechanical damage, pH changes). Metal ions such as Fe^3+^ form coordination bonds with the ─OH and ─COOH groups of catechols, which reversibly break and recombine in response to pH changes or redox reactions, giving the material the ability to self‐repair.^[^ ^82^ ^]^

MPNs are protonated by the phenolic hydroxyl groups under acidic conditions, which weakens the binding to the metal ions and could lead to network dissociation or size reduction. The binding is recovered under alkaline conditions. TA and Fe^3^⁺ complexes dissociated at pH < pKa, led to network disintegration, and re‐bind under alkaline conditions, enabling materials reconfiguration. TA/Fe coatings temporarily protected probiotics in the gastrointestinal tract against gastric acid and antibiotics through pH‐responsiveness.^[^ ^83^ ^]^ In addition, polyphenols (e.g., catechol) could contribute to the adhesion of biomaterials by generating quinone groups through oxidation and forming more stable complexes with metal ions, as well as participating in free radical reactions as electron donors. MPNs are of considerable interest in the fields of material science, environmental science, and drug delivery, attributing to their unique properties such as pH responsiveness, anti‐oxidative ability, and bioactivity. The versatility of MPNs allows for their application in various areas, including catalysis, tissue engineering, and the creation of functional coatings and films.^[^ ^1^, ^84^ ^]^

### 3.3 Boronate–Phenolic Network

Boronate–phenolic network is a dynamic covalent interaction in which the phenolic hydroxyl group is coordinated to a boronic acid or boronic ester to form a reversible covalent bond, as shown in Figure 4f. The core mechanism is that the boron atoms are bonded to the hydroxyl group of the polyphenol through shared electron pairs, and the reaction is reversible and dependent on pH conditions.^[^ ^85^ ^]^ Phenolic compounds are able to efficiently construct borate complexes via dynamic covalent bonding by virtue of the stereochemical ordering of the hydroxyl groups on the aromatic ring and the strong electron–donor property.^[^ ^10^ ^]^ These complexes are stable at alkaline pH, but dissociate at acidic pH or in the presence of exogenous competition. Liu et al.^[^ ^86^ ^]^ designed pH‐responsive antimicrobial hydrogels for catechol–borate complexation with reversible borate bonds formed between catechol chloride and phenylboronic acid. At acidic pH, catechol chloride dissociated from phenylboronic acid with a bacterial killing effect, while at alkaline pH conditions, it was not toxic to bacteria. In addition, a previous study had developed a pH dynamically responsive wound microenvironment and on‐demand drug release hydrogel dressing with matrix materials based on aminophenylboronic acid‐functionalized alginate and polyhydroxy polymers, which utilized dynamic boronic acid ester bonding and catechol metal ion ligand bonding, and exhibited significant shape adaptation, self‐repairing ability, tissue adhesion, antioxidant activity, and photothermal responsiveness.^[^ ^87^ ^]^

### 3.4 Covalent Interaction

Polyphenolic compounds are susceptible to oxidative coupling reactions by oxidizing agents (e.g., oxidizing reagents, pH fluctuations, enzymes, etc.) or thermodynamic conditions (e.g., elevated temperatures) to form quinone compounds.^[^ ^81^, ^88^ ^]^ The Michael addition is a type of nucleophilic addition reaction, where a nucleophile adds to an α, β‐unsaturated carbonyl compound, typically a conjugated enones or enals. The mechanism involves the attack of a nucleophile, such as a thiol, amine, or alkoxide, to the β‐carbon of the conjugated system, resulting in the formation of a new bond^[^ ^89^ ^]^(Figure 4g(I),(II)). For example, in a high‐temperature environment with the presence of Cu^2+^, the catechol and pyrogallol moieties of EGCG were converted to the corresponding highly reactive semiquinones and quinones. This was followed by the formation of dehydrocatechins with interflavanine linkages mainly through nucleophilic addition reactions of the quinones.^[^ ^90^ ^]^ In addition, under alkaline conditions, ovalbumin could be linked between the amino group and the phenolic group of TA by Schiff base reaction/nucleophilic addition reaction to form TA‐ovalbumin nanoconjugates.^[^ ^91^ ^]^ Nucleophilic addition/Schiff base reactions strengthened the network structures of the hydrogel, as shown in Figure 4g(III). The catechol moiety in gallic acid was readily oxidized to the quinone form, which then reacted with the amine and hydroxyl groups of chitosan via Schiff base and Michael addition reactions, respectively, to form a 3D hydrogel network with high mechanical strength and adhesive properties.^[^ ^92^ ^]^ Besides, when polyphenols undergo oxidative activation to form phenoxy radicals, they combine with other substances and undergo free radical coupling reactions to form conjugates, as shown in Figure 4g(IV).^[^ ^93^ ^]^ For example, the coupling reaction of carbodiimide with 1‐(3‐dimethylaminopropyl)‐3‐ethylcarbodiimide hydrochloride and N‐hydroxysuccinimide covalently bonded the amine group of 5‐hydroxydopamine to the carboxyl group of the hyaluronic acid backbone to form a structurally stable hydrogel.^[^ ^94^ ^]^

## 4 Self‐Assembly Strategies of Polyphenols

At present, polyphenols, either alone or in combination with diverse bioactive substances, have been widely explored for self‐assembly into functional structures such as nanoparticles,^[^ ^95^ ^]^ hydrogels,^[^ ^96^ ^]^ and capsules^[^ ^97^ ^]^ due to their specific chemical structural properties and versatile interaction capabilities. These self‐assembled systems are designed to enhance antibacterial efficacy, reduce toxicity, improve bioavailability, and optimize therapeutic outcomes, thereby representing a rapidly advancing area of research. This has undoubtedly emerged as a rapidly evolving field. Self‐assembly of polyphenol compounds possesses numerous advantages, including high drug loading ratios,^[^ ^98^ ^]^ excellent biocompatibility, controllable degradability, and pharmacological activities with synergistic effects.^[^ ^99^ ^]^ After that, the emphasis will be placed on discussing the assembly strategies of polyphenols with metals, proteins, polysaccharides, other polyphenols, and other natural products, to deepen the comprehension of polyphenol self‐assembly behavior and provide a theoretical foundation for formulating targeted self‐assembly strategies.

### 4.1 Self‐Assembly of Polyphenols with Metal Ions

Polyphenols and metals could be assembled through spontaneous coordination‐driven processes to form MPNs, where metal ions serve as electron acceptors and phenolic compounds act as electron donors. When two or more phenolic ligands offer non‐bonding electron pairs to the empty orbitals of metal ions, metal–phenol coordination is generated.^[^ ^100^ ^]^ The phenolic moieties could have diverse interactions with various molecules and substrates (including coordination interactions, hydrogen bonds, electrostatic interactions, redox reactions, etc.). Consequently, the diverse phenolic ligands and metal ions utilized in the MPNs assembly process could easily produce polyphenolic materials with different shape–structures (such as nanoparticles, coatings, films, capsules, and hydrogels) and specific functional characteristics (**Table** **1**).

**Table 1:** Different structures of MPNs formed by polyphenols and metal ions.

Phenolic ligands	Metal ion	Structure	Main interaction	Functional characteristics	Refs.
EGCG	Cu^2+^	Nanoparticle	Hydrogen bonding, metal coordination bond, nucleophilic addition reaction	Antioxidant and anti‐inflammatory	[102]
EGCG	Sm^3+^	Nanoparticle	Hydrogen bonding, metal coordination bond	Antitumor	[103]
TA	Co^2+^, Pt^2+^	Nanoparticle	Hydrogen bonding, electrostatic effect, and metal coordination bond	Photothermal performances	[104]
Quercetin	Fe^2+^	Nanoparticle	Hydrogen bonding, metal coordination bond	Photothermal performances, antimicrobial	[106]
TA	Fe^3+^	Film or Coating	Hydrogen bonding, electrostatic effect, and metal coordination bond	pH response	[108]
TA	Fe^3+^	Film or Coating	Hydrogen bonding, electrostatic effect, and metal coordination bond	High adhesiveness	[109]
TA, catechol, pyrogallol, etc.	Fe^2+^	Film or Coating	Hydrogen bonding, metal coordination bond	pH response, antioxidant	[110]
GA	Fe^2+^	Film or Coating	Hydrogen bonding, metal coordination bond, and electrostatic effect	pH response,	[112]
TA	Cu^2+^, Fe^3+^	Film or Coating	Hydrogen bonding, metal coordination bond, electrostatic effect, hydrophobicity	pH response, photothermal performances, and antitumor	[11]
TA	Gd^3+^	Film or Coating	Hydrogen bonding, metal coordination bond, and electrostatic effect	pH response, photothermal performances, and antitumor	[113]
TA	Fe^3+^	Capsule	Hydrogen bonding, metal coordination bond, and electrostatic effect	pH response	[116]
TA	Al^3+^	Capsule	Metal coordination bond,	pH response	[118]
Quercetin	Fe^2+^	Capsule	Hydrogen bonding, metal coordination bond, and electrostatic effect	pH response, photothermal performances,	[119]
TA	Fe^3+^	Hydrogel	Hydrogen bonding, metal coordination bond	High gel strength, strong water retention capacity	[121]
TA, dopamine	Fe^3+^	Hydrogel	Hydrogen bonding, metal coordination bond, and electrostatic effect	Thermoplastic reversibility, self‐healing properties, electrical conductivity	[122]
TA	Fe^3+^	Hydrogel	Hydrogen bonding, metal coordination bond, and hydrophobic interactions	High mechanical strength and photothermal effect	[123]

#### 4.1.1 Nanoparticles

**Figure 5:**
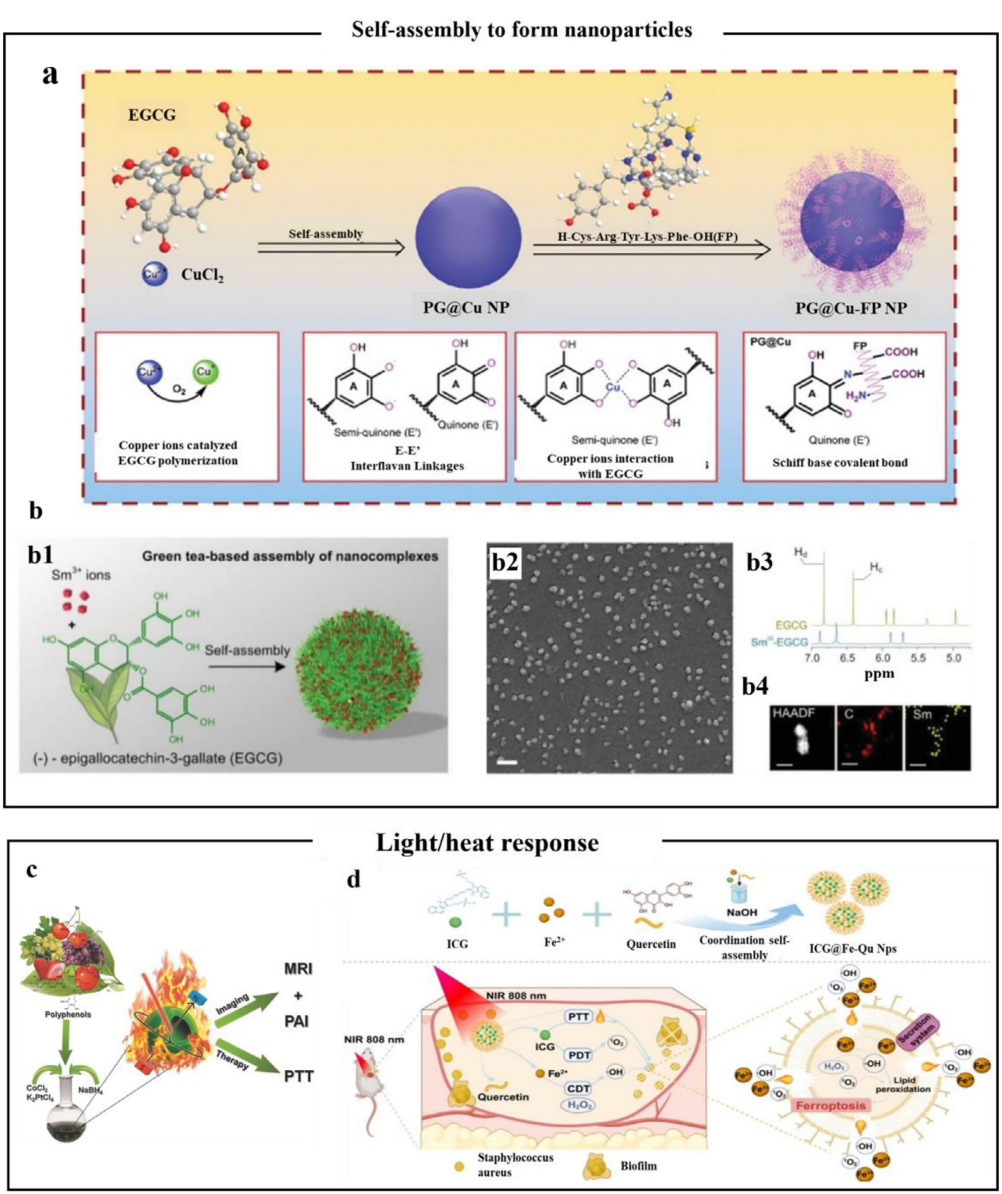
Self‐assembly formation and environmentally responsive release of metal polyphenol nanoparticles. a) Self‐assembly of polyphenols with metals to form MPN structures.^[^ ^102^ ^]^ Copyright 2023, Wiley. (b1) Easy synthesis of Sm^3+^‐EGCG nanocomplexes by self‐assembly. (b2) Scanning electron microscopy images of narrowly dispersed Sm^3+^‐EGCG nanocomplexes with a shape close to a sphere, scale bar is 100 nm. (b3) ^1^H NMR spectra of EGCG molecule and Sm^3+^‐EGCG nanocomplex showing coordination complexation between the galloyl group of EGCG and Sm^3+^ ion. (b4) HAADF‐TEM and EDS spectra of Sm^3+^‐EGCG nanocomplex. Scale bar is 50 nm.^[^ ^103^ ^]^ Copyright 2019, Wiley‐VCH. c) Schematic representation of the synthesis and therapeutic application of HCPA‐NPs.^[^ ^104^ ^]^ Copyright 2016, Wiley. d) Schematic representation of the synthesis process and antimicrobial mechanism of ICG@Fe‐Qu nanoplatforms.^[^ ^106^ ^]^ Copyright 2024, Elsevier.

Phenolic compounds exhibit a strong chelating affinity for multivalent metal ions, facilitating the spontaneous formation of nanoparticles with well‐defined structures through mild and controllable self‐assembly processes. Polyphenolic compounds containing multiple hydroxyl or carboxyl groups (such as GA and TA) could combine with certain metal ions (Cu^2+^, Fe^3+^) to form nanoparticles under different pH environments.^[^ ^101^ ^]^ For example, EGCG and Cu^2+^ oxidatively polymerized to become a quinone structure in an alkaline environment, which self‐assembled into nanoparticles (**Figure** **5**a).^[^ ^102^ ^]^ The nanoparticles have antioxidant and anti‐inflammatory properties, and work synergistically with peptide drugs to enhance mitochondrial targeting and inhibition of pyroptosis. Sm^3^⁺–EGCG nanoparticles were formed through ion coordination between EGCG and Sm^3^⁺ in a pH‐neutral solution, self‐assembling into a nearly spherical shape (Figure 5b). Furthermore, the ^1^H NMR spectrum showed characteristic peaks Hd and Hc in the 5.5–7.0 ppm range, which shifted to lower field positions after chelation with Sm^3^⁺ ions. This shift confirmed the complexation of EGCG with metal ions.^[^ ^103^ ^]^ The incorporation of metal ions could confer upon MPNs nanostructures distinctive magnetic, optical, and other properties. A study used TA as a ligand to form chelates with hollow cobalt–platinum alloy nanoparticles through metal coordination bonds, thereby prepared metal–polyphenol hollow nanoparticles.^[^ ^104^ ^]^ Results had indicated that these nanoparticles exhibited superior photothermal performance, strong near‐infrared absorbance, and excellent photothermal transduction efficiency, which had the capacity for magnetic resonance imaging/photoacoustic imaging dual‐mode imaging and photothermal therapy therapeutic drugs (Figure 5c). Additionally, studies have reported that metal–polyphenol nanoparticles exhibit photothermal properties.^[^ ^105^ ^]^ Liu et al.^[^ ^106^ ^]^ designed an innovative metal–polyphenol antimicrobial nanoparticle that effectively accelerated epithelialization of skin infection wounds under near‐infrared irradiation, promoted collagen deposition, and achieved rapid healing of infected wounds (Figure 5d). One reason for this was that near‐infrared irradiation generated localized heat that disrupted bacterial membranes, thereby facilitating the penetration of iron ions into bacterial cells. This was followed by the Fenton reaction, which produced a large amount of bacterial iron death.

#### 4.1.2 Film and Coating

**Figure 6:**
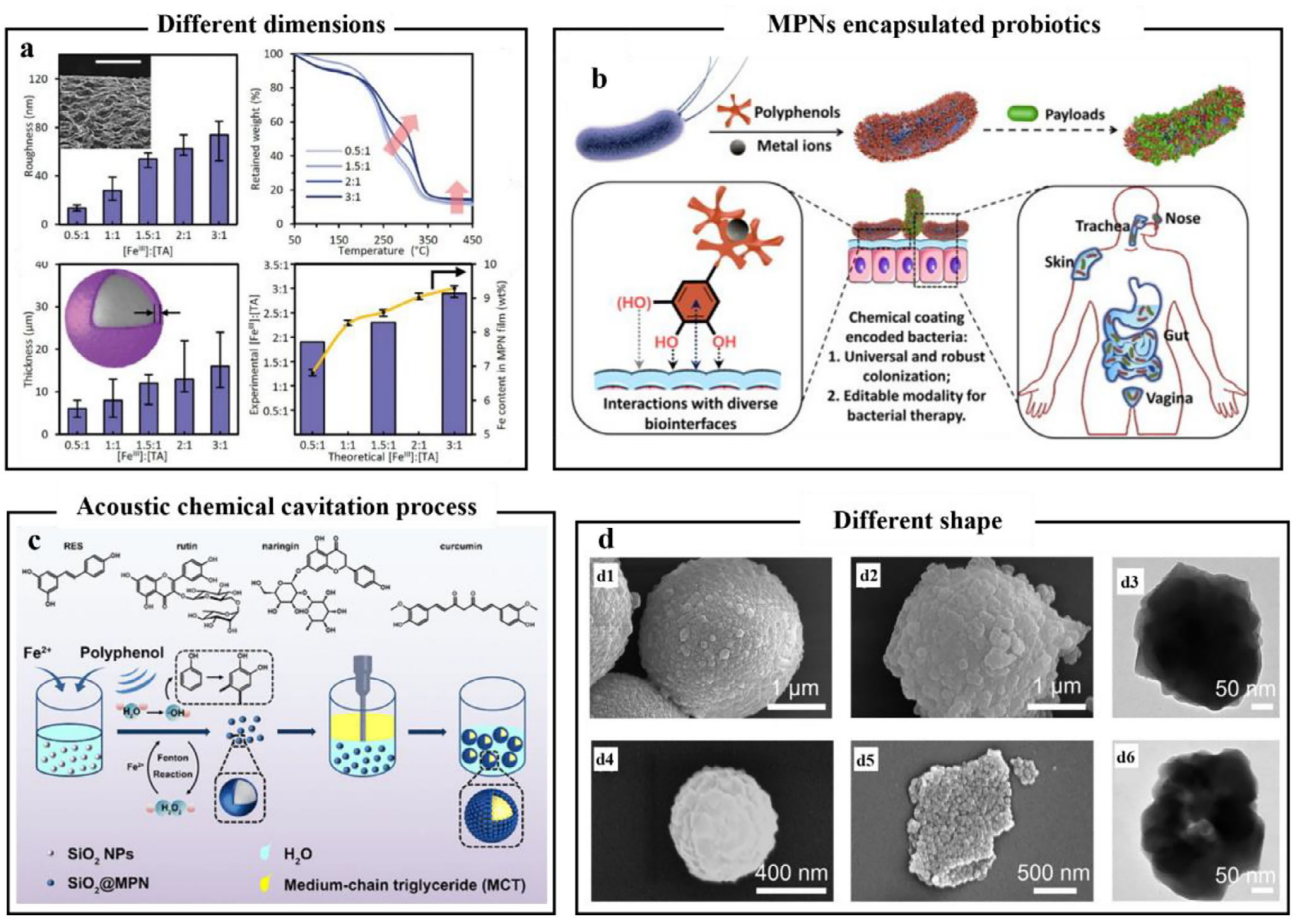
Self‐assembly formation of metal polyphenol films and coatings. a) Effect of different ratios on the formation of MPN films.^[^ ^108^ ^]^ Copyright 2022, Wiley. b) Schematic representation of the preparation of TA‐encapsulated bacteria by complexation with Fe^3+^ supramolecular and interaction with various bio‐interfaces in the host body.^[^ ^109^ ^]^ Copyright 2023, Elsevier. c) Schematic representation of the preparation of MPNs formed by different polyphenolic materials to stabilize the Pickering emulsion with SiO_2_ NPs.^[^ ^110^ ^]^ Copyright 2024, American Chemical Society. d) SEM and TEM images of crystalline MOF coatings on different MPN‐modified substrates including melamine formaldehyde (MF) (d1), CaCO_3_ (d2), gold nanoparticles (AuNPs) (d3), aminated silica (NH2‐SiO_2_) (d4), graphene oxide (GO) (d5), and silver nanoparticles (AgNPs) (d6).^[^ ^111^ ^]^ Copyright 2024, Wiley.

MPNs, known for their simplicity, safety, and cost‐effectiveness, have demonstrated broad applicability across a wide range of substrates. Through specific processing steps, they could be coated on various nanoscale and microscale objects (metal nanoparticles, CaCO_3_, SiO_2_, probiotics).^[^ ^107^ ^]^ The controllable formation of films often demands slow deposition conditions or multiple coating cycles, conferring the films with dense and high‐strength characteristics. The combination of TA and Fe^3+^ to form MPNs coatings is frequently observed in research. Mazaheri et al.^[^ ^108^ ^]^ reported that TA and Fe^3+^ could form MPNs through coordination‐driven self‐assembly and form coatings on the surface of urea particles. Moreover, the coating thickness was designed based on the ratio of TA and Fe^3+^ (**Figure** **6**a). Additionally, as coatings, metal–polyphenol networks exhibited high adhesion performance. There was a research report that indicated that by repeatedly adding 10 µL of FeCl_3_ solution (10 mg mL^−1^) and 10 µL of TA solution (30 mg mL^−1^) to a bacterial suspension and stirring after each addition, a stable TA‐Fe^3+^ coating was formed on the bacterial surface (Figure 6b).^[^ ^109^ ^]^ As coatings, MPNs have high adhesion characteristics, mainly attributed to the unstable surface charges of different matrix cores, which exert an adsorption force on the MPNs formed by the combination of metals and polyphenols. One research had reported that using hydrophilic SiO_2_ nanoparticles as templates and selecting various phenolic compounds (TA, catechol, pyrogallol, rutin, naringin, and curcumin), combined with Fe^2+^, it was found that the MPNs structure formed a film on the SiO_2_ surface. This was because the continuous generation of hydroxyl radicals (·OH) through the sonochemical cavitation process promoted the phenolation of phenolic compounds, and the moist and charge‐imbalanced SiO_2_ surface attracted the MPNs structure to form an MPN coating (Figure 6c).^[^ ^110^ ^]^ Furthermore, MPNs, as an interface layer, assisted in the deposition and growth of metal–organic framework (MOF) coatings on different particle surfaces, and the thickness and morphology of the coating could be adjusted according to different synthesis conditions (Figure 6d).^[^ ^111^ ^]^ Interactions such as hydrogen bonding, metal coordination bonds, electrostatic effects, and hydrophobicity enable MPN to have immobilization capabilities. Since polyphenols and metal ions form MPNs through multiple chemical interactions, the stability of MPNs is influenced by chemical substances in the solution environment. Based on this mechanism, responsive nano‐coatings have been extensively studied. Dong et al.^[^ ^112^ ^]^ reported the strong coordination interaction between GA and iron ions (Fe^2+^), and prepared pH‐dissociable metal–polyphenol‐network‐coated CaCO_3_ hollow nanoparticles (GA‐Fe@CaCO_3_) using biocompatible amorphous CaCO_3_ nanoparticles as templates. When accumulated in the acidic tumor microenvironment (pH 6.5–7), the TA‐Fe network structure weakened, and the nanoparticles shrunk to a smaller size, thereby achieving deeper intratumor penetration. In addition to pH‐responsive delivery of drugs or bioactive substances to enhance drug efficacy and sensitivity, currently, one research encompassed the dual‐responsive release of drugs via the combination of photothermal and pH, which enhanced cancer and tumor treatment.^[^ ^113^ ^]^ CuS with photothermal conversion properties served as the core material, and TA and metal ions (Fe^3+^) act as the acid‐sensitive shell, forming nanoparticles loaded with the drug doxorubicin. In the tumor microenvironment, the acidic pH combined with photothermal stimulation led to MPN shell dissociation and controlled doxorubicin release, thereby enhancing the therapeutic efficacy through synergistic chemo–photothermal treatment.^[^ ^11^ ^]^

#### 4.1.3 Capsule

**Figure 7:**
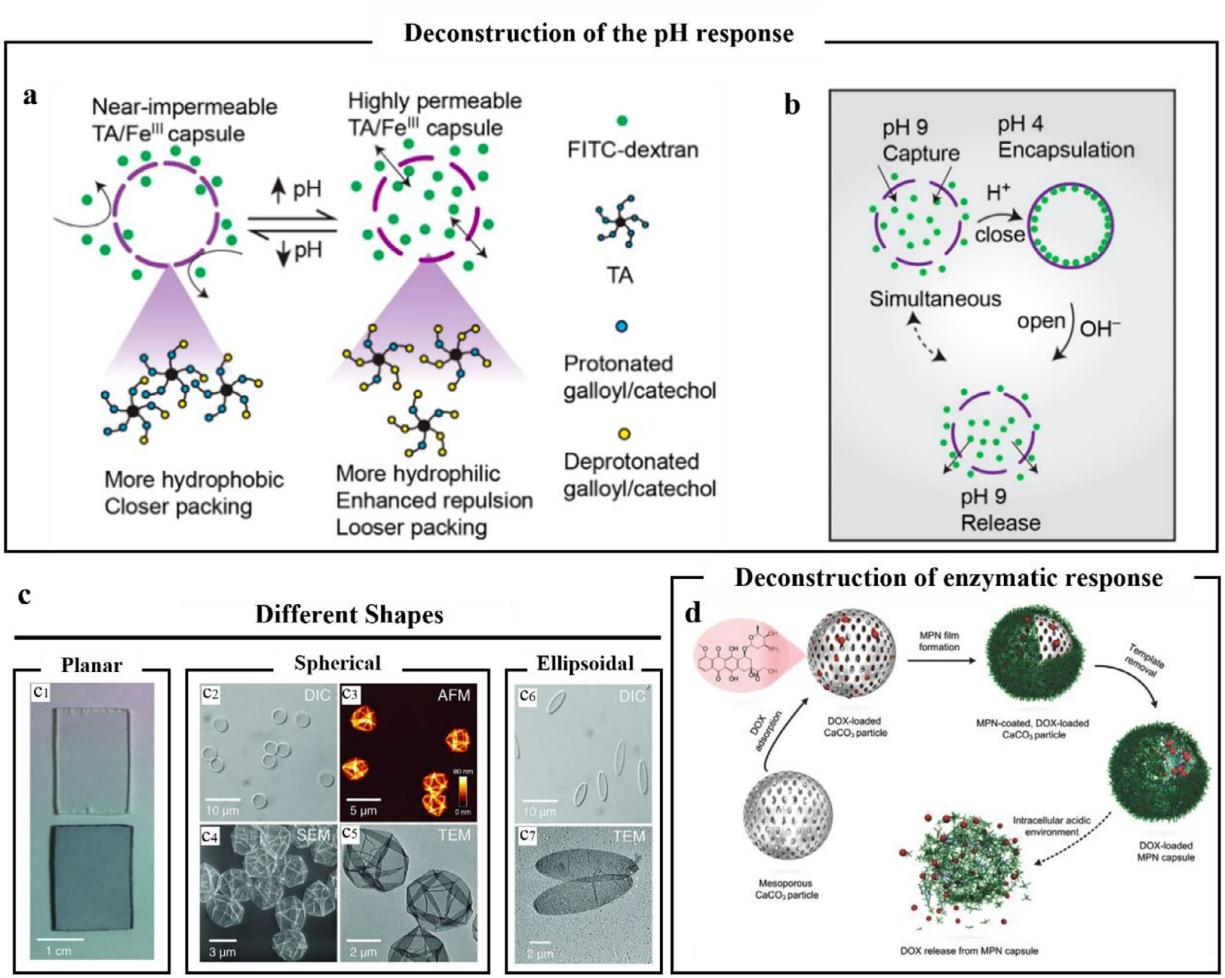
Self‐assembly of metal polyphenols to form capsules and pH‐responsive release. a) Schematic of the effect of pH on capsule permeability. b) Schematic of pH‐modulated switchable permeability of TA/Fe^3+^ capsules for cargo encapsulation and release.^[^ ^117^ ^]^ Copyright 2020, American Chemical Society. c) On planar, spherical, and elliptical PS substrates prepared on Fe^3+^‐TA films.^[^ ^116^ ^]^ Copyright 2013, Science. d) Schematic of the preparation process of DOX‐loaded MPN capsules and the release mechanism of (doxorubicin) DOX from MPN capsules.^[^ ^118^ ^]^ Copyright 2015, Wiley.

**Figure 8:**
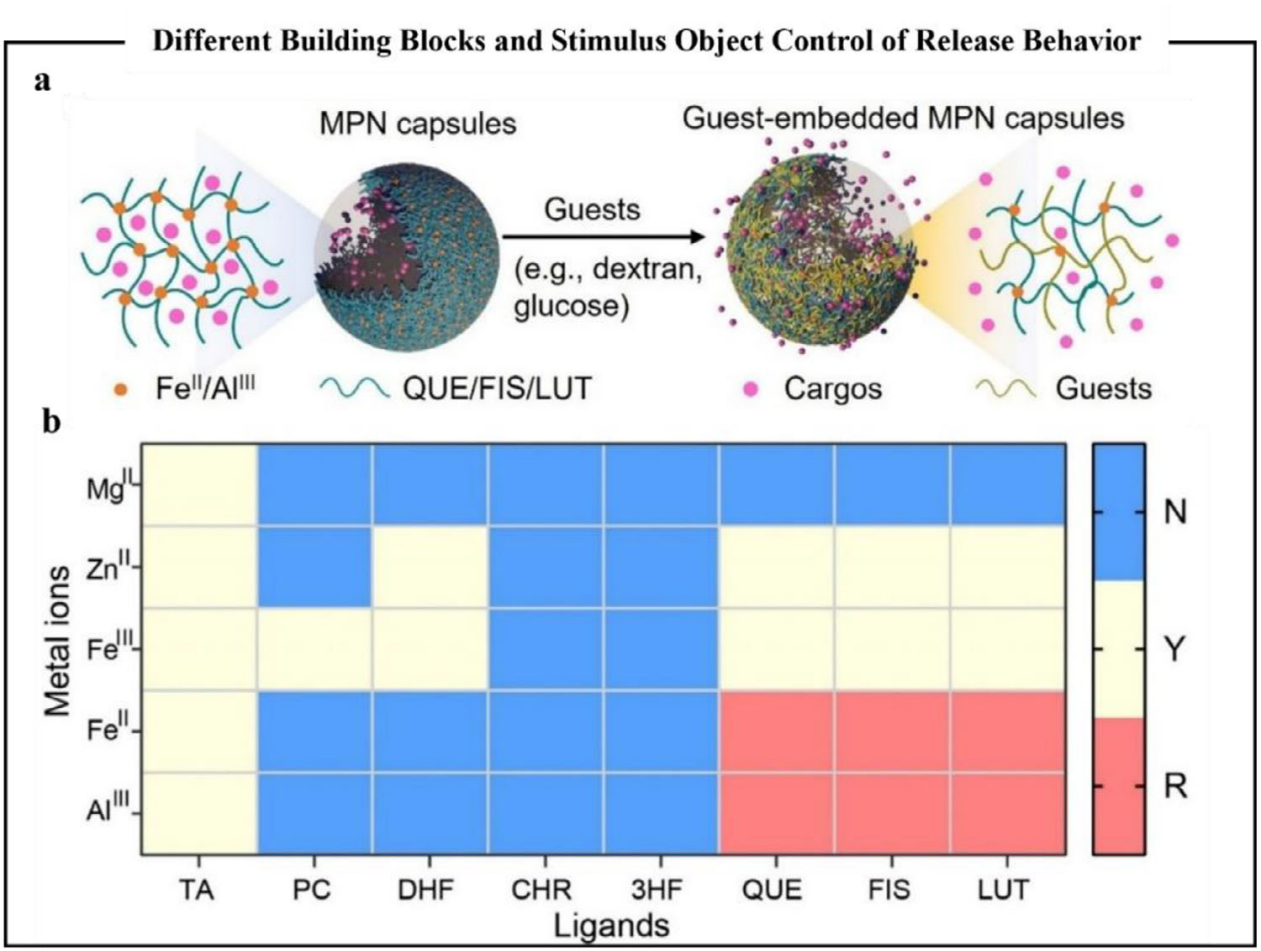
Stimulus object with different building blocks controlling the release behavior. a) Schematic diagram of the structural remodeling of the MPN capsule when stimulated by an external object; b) Heatmap showing the MPN capsule formed by different metal ions and phenol ligands and their corresponding responses to 100 mM Glu solution. N, no capsule formed; Y, capsule formed; R, capsule formed in response to Glu.^[^ ^119^ ^]^ Copyright 2023, Wiley.

The formation of MPNs hollow capsules bears resemblance to that of MPNs coatings. The reason for the formation of hollow capsules is that after MPNs form a film on the surface of the particle template, it can be separated from the surface of the particle template through specific treatment approaches while maintaining its structural integrity.^[^ ^97^, ^114^ ^]^ Various types of capsules based on MPNs have been extensively reported, and the majority of them were fabricated by one‐step deposition of MPNs on particle templates.^[^ ^115^ ^]^ It had been previously reported that the mixture of TA and Fe^3+^ in water led to film deposition on the template particles. Subsequently, the dissolution of the template resulted in the formation of 3D independent films, namely hollow capsules (**Figure** **7**c).^[^ ^116^ ^]^ However, during the process of template particle removal, it might influence the refilling of functional substances within the hollow interior and the in situ encapsulation of guest molecules during the shell formation process. In order to avoid this issue, Tan et al.^[^ ^115^ ^]^ reported that based on bovine serum albumin microbubbles as soft templates and carriers, the addition and coordination assembly of TA and Fe^3+^ ions could induce conformational changes in bovine serum albumin protein, forming translucent microcapsules. This method eliminated the need for template removal and pre‐encapsulated functional substances in a single step, thereby achieving favorable encapsulation effects. To investigate the enhancement of drug delivery and the separation effect between drugs and carriers by regulating the permeability of capsules, which is of great research value in drug delivery. Researchers examined the effects of internal factors (such as the ratio of polyphenols to metal ions, the type of metal, and phenolic ligands) and external factors (such as pH) on the permeability of MPN capsules.^[^ ^117^ ^]^ The findings revealed that 1) the permeability of the capsules increased with the molar ratio of Fe^3^⁺: TA, and nanoscale defects were observed. 2) When comparing different metal ions, it was discovered that Al^3+^ and Cu^2+^ did not bind tightly with TA, resulting in the shrinkage of the capsule size after the removal of the template particles, which was detrimental to their permeability. Due to the presence of multiple phenolic hydroxyl groups in TA, it underwent deprotonation at higher pH, and the intermolecular electrostatic repulsion intensified, thereby causing the expansion of pores in the membrane (Figure 7a). 3) The differences in permeability caused by varying pH values suggested that TA/Fe^3+^ capsules could be designed for programmable cargo encapsulation and release. At pH 4, the permeability state of TA/Fe^3+^ capsules was “closed,” and at pH 9, the permeability state of TA/Fe^3+^ capsules was “open” (Figure 7b). It was displayed that the permeability of MPN capsules was programmable under different environmental conditions. Thus, the permeability of MPNs capsules was programmed to adjust based on environmental pH, enhancing their targeted delivery efficacy. For example, there was research demonstrated that drug‐loaded MPN capsules, which were based on the formation of coordination complexes between TA and Al^3+^ ions, could be assembled and used as pH‐responsive carriers for drug loading and intracellular drug release, as illustrated in Figure 7d.^[^ ^118^ ^]^ Naturally, in addition to the response mechanisms of controlled release and degradation under pH and photothermal conditions, it was also formed by intermolecular competitive coordination. Xu et al.^[^ ^119^ ^]^ investigated the MPNs composed of Fe^2+^ and quercetin, namely Fe^2+^‐quercetin MPNs, which exhibited significant guest‐responsive behavior (**Figure** **8**a). The researchers systematically studied the formation of capsules by varying the types of metal ions and polyphenols, and results verified that ligands with multimodal coordination sites and metal ions with appropriate binding affinities were crucial for the fabrication of Glu‐responsive MPNs (Figure 8b).

#### 4.1.4 Hydrogel

**Figure 9:**
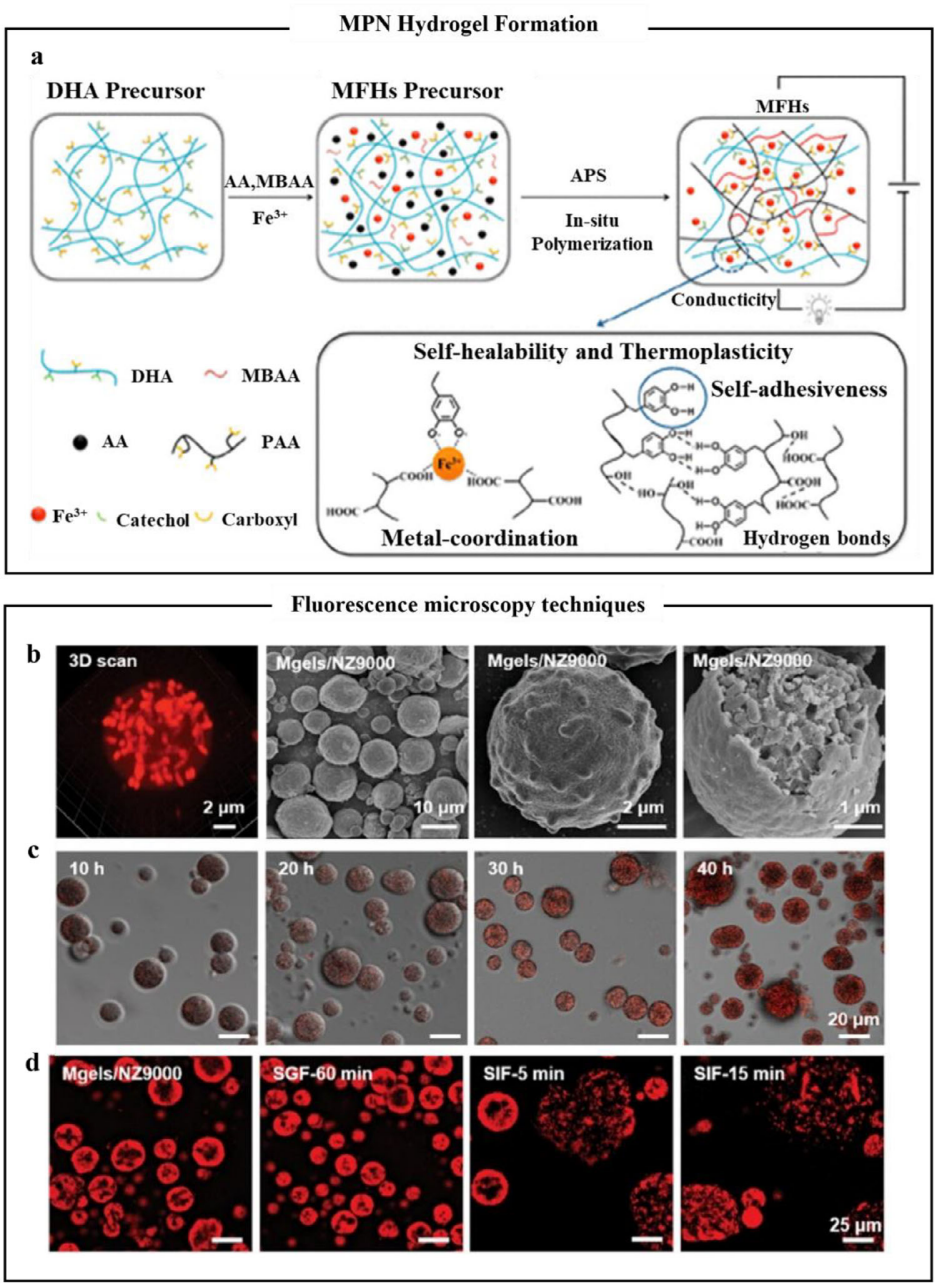
Self‐assembly of metal polyphenol networks to form hydrogels and pH response. a) Schematic of the synthesis process of MFHs hydrogels and the corresponding interactions within the multifunctional networks.^[^ ^122^ ^]^ Copyright 2019, American Chemical Society. b) CLSM and SEM images of NZ9000 (red) loaded MCPC microgels. The CLSM image shows the growth of NZ9000 in a microgel in M17 broth (pH 5.5). c) CLSM image showing the growth of NZ9000 in a microgel in M17 broth (pH 5.5). d) Fluorescence image of MCPC/NZ9000 microgel before and after treatment in SGIF.^[^ ^124^ ^]^ Copyright 2024, Wiley.

For years, hydrogels have been a big deal for biomaterials scientists because of their great hydrophilicity and biocompatibility. Polyphenols, with their multi‐hydroxy structure, can bind with other materials through non‐covalent or covalent interactions. The combined design of MPN and hydrogels could augment the mechanical strength, adhesion, and responsiveness of hydrogels and offer dual‐layer protection for the substrate. For instance, Mao et al.^[^ ^120^ ^]^ developed a sodium alginate hydrogel with an MPN network structure, in which Fe^3^⁺ and anthocyanin self‐assemble to form an MPN structure under the action of hydrogen bonds and metal coordination bonds, enhancing the hydrogel`s high gel strength and water retention capacity. Zhou et al.^[^ ^121^ ^]^ developed a novel probiotic hydrogel system (Gel/L@FeTA) with high adhesion and mechanical strength, where a layer of metal–polyphenol complex (Fe/TA) was overlaid on the surface of probiotics, forming a dual‐network hydrogel in conjunction with carboxylated chitosan and oxidized hyaluronic acid. The incorporation of MPN confers smart responsiveness to the hydrogel. For example, a multifunctional conductive hydrogel was developed by incorporating Fe^3^⁺ ions into dopamine‐modified hyaluronic acid, wherein the catechol groups of dopamine served as active coordination sites. The abundant hydrogen bonding and strong metal coordination between catechol and Fe^3^⁺ endowed the resulting hydrogel with thermoplastic reversibility and excellent self‐healing properties in both mechanical and electrical domains (**Figure** **9**a).^[^ ^122^ ^]^ In addition, TA and Fe^3+^ could help construct hydrogels with controllable photothermal effects. Under near‐infrared irradiation, the hydrogels displayed programmed deformation using real‐time spatio–temporal precision and remote control. MPNs have been extensively studied for enhancing the biocompatibility and adhesion of hydrogels.^[^ ^123^ ^]^ Zhang et al.^[^ ^124^ ^]^ reported that the chelation of Fe^3+^ with mesona chinensis polysaccharide (MCPC) formed microgels. Due to the rich polyphenolic brown active substances in MCPC coordinating and chelating with Fe^3+^, the surface strength of the microgels was increased. Owing to the pH‐responsive property of metal–polyphenol coordination, the release was slow in gastric juice, which enhanced the colonization ability of probiotics in the gastrointestinal tract (Figure 9b–d). Thereby, conventional hydrogels (formed by polysaccharides and proteins) could utilize MPNs to enhance drug colonization, colonization time, and response release at specific sites.

### 4.2 Self‐Assembly of Polyphenols with Proteins

Proteins are biomolecules composed of amino acids linked by peptide bonds. The functional groups (e.g., amino groups, carboxyl groups, and side chain R groups) confer unique physiological functions on proteins. Polyphenols and proteins interact with each other through both covalent and non‐covalent forms in the process of binding, and either form could improve the physicochemical characteristics and functional properties to a certain extent.^[^ ^125^ ^]^ Covalent interactions generally occur in oxidative environments where the phenolic hydroxyl groups of polyphenols are oxidized to quinones. These quinones could subsequently react with nucleophilic amino or sulfhydryl side chains in polypeptides, forming stable covalent complexes. Generally, polyphenols are known to be excellent hydrogen donors that form hydrogen bonding interactions with C═O, ─OH, and ─NH_2_ of proteins.^[^ ^125^ ^]^ Additionally, electrostatic interaction is also the main force for the formation of polyphenol–protein complex particles.^[^ ^126^ ^]^ There are numerous factors (including temperature, pH, protein type, and the specific type and structure of the phenolic compound) that can influence the non‐covalent interactions between polyphenols and proteins.^[^ ^127^ ^]^ Under these forces, polyphenols and proteins could form functional materials with diverse structural shapes, such as nanoparticles, coatings, capsules, and hydrogels (**Table** **2**).

**Table 2:** Different structures of polyphenol–protein.

Phenolic ligands	Protein	Structure	Main interaction	Functional characteristics	Refs.
TA	TANNylated green fluorescent protein (GFP)	Nanoparticle	Hydrogen bonding, hydrophobic interaction, nucleophilic addition reaction	Increase targeted action	[128]
TA	Zein	Nanoparticle	Hydrogen bonding, hydrophobic interaction	Improving the stability of emulsions	[129]
EGCG	Zein	Nanoparticle	Hydrogen bonding, hydrophobic interaction	Improving the stability of emulsions	[130]
TA	poly(lysine) (PLys), PArg, poly(histidine) (PHis), poly (glutamic acid) (PGlu), etc.	Coating or capsule	Hydrogen bonding, electrostatic effect	pH response, efficient delivery	[131]
TA	Zein	Coating or capsule	Hydrogen bonding, hydrophobic interaction, nucleophilic addition reaction	pH response, efficient delivery	[132]
TA	Anionic peptides (Asp6), neutral hydrophilic peptides (Gly6), neutral hydrophobic peptides (Ile6 and Phe6), etc.	Coating or capsule	Hydrogen bonding, electrostatic effect	pH response, high adhesiveness, antibacterial properties	[133]
TA	Gelatin	Hydrogel	Hydrogen bonding, hydrophobic interaction, Schiff base reaction	Good elasticity, high phase transition temperature, high adhesiveness, and good ionic conductivity	[135]
EGCG	Honey bee pupal protein (HBPP)	Hydrogel	Hydrogen bonding	High adhesiveness	[136]

#### 4.2.1 Nanoparticles

**Figure 10:**
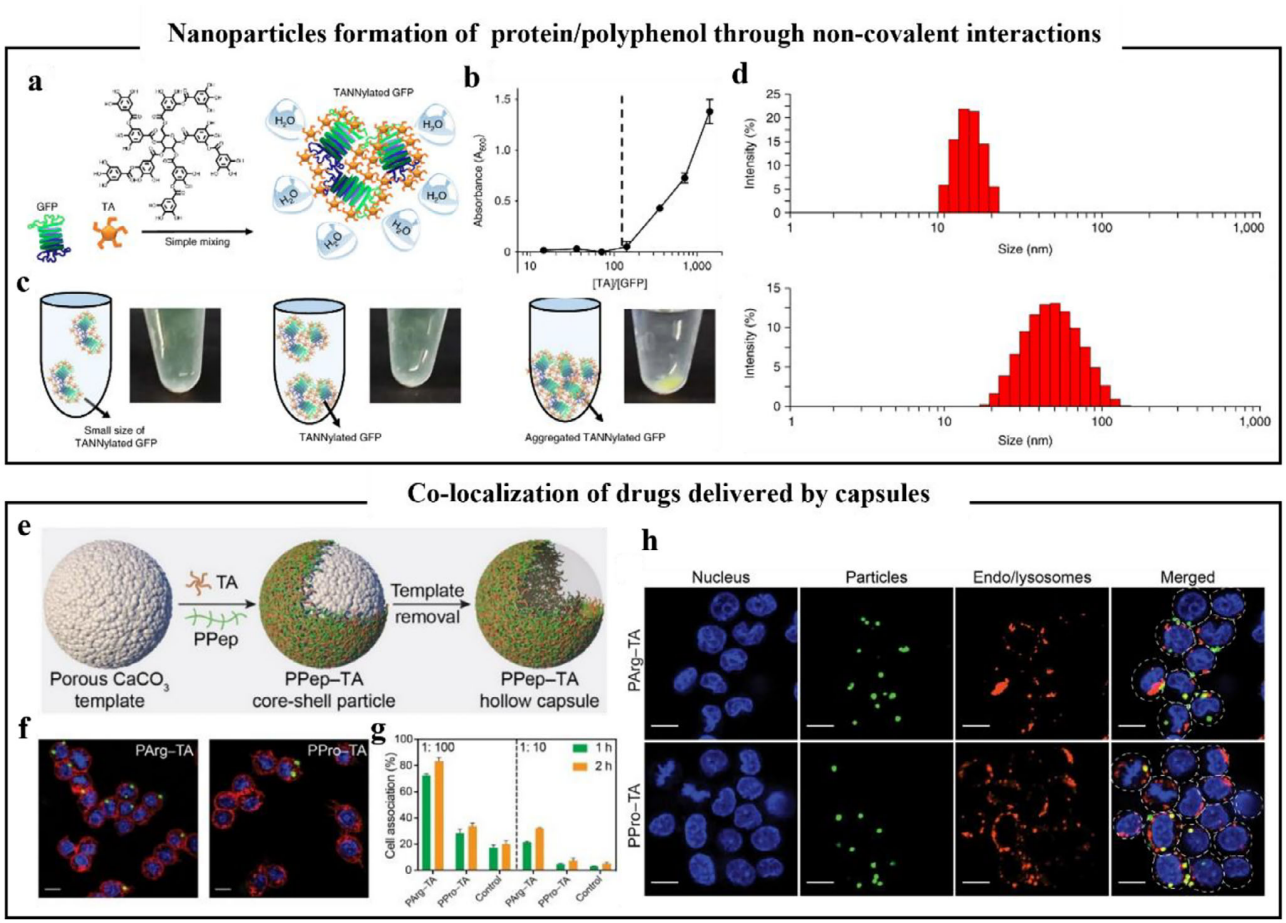
Polyphenols self‐assemble with proteins to form delivery vehicles. a) Schematic of the preparation of TANNylated GFP. b) Specific turbidity of the critical stoichiometric ratio of [TA]/[GFP]. The dashed line indicates a stoichiometric ratio of [TA]/[GFP] of 143. c) Images of TANNylated GFP with [TA]/[GFP] stoichiometric ratios of 72 (left), 143 (center), and 357 (right). d) DLS images of TANNylated GFP in the nanoscale complexes at [TA]/[GFP] stoichiometric ratios of 72 and 143.^[^ ^128^ ^]^ Copyright 2018, part of Springer Nature. e) Schematic diagram of PPep‐TA core–shell particles and hollow capsule formation. f) Confocal laser scanning microscopy (CLSM) images of PArg‐TA and PPro‐TA particles after 1 h of co‐incubation with HeLa cells. Scale bar is 10 µm. g) Percentage of cells incorporating particles after co‐incubation of HeLa cells with PArg‐TA, PPro‐TA particles, and TA‐coated particles (control) for 1 or 2 h, respectively, as assessed by flow cytometry. The cell‐to‐particle ratios were 1:100 and 1:10, respectively, and the data are expressed as the mean ± standard deviation of three independent experiments. h) CLSM imaging results of LysoTracker co‐localization experiments performed after 4 h of co‐incubation of HeLa cells with PArg‐TA or PPro‐TA particles.^[^ ^131^ ^]^ Copyright 2022, American Chemical Society.

In protein‐based drug delivery systems, the incorporation of polyphenols regulated the sizes of the carriers based on pH, thereby affecting the efficiency of targeted drug delivery. For example, Shin et al.^[^ ^128^ ^]^ mixed recombinant protein (Tannic‐acid‐modified green fluorescent protein, (GFP)) with TA to form the polyphenol protein complex particles (**Figure** **10**a,b). In an alkaline solution, nanosized particles were present. In contrast, in acidic solution, thereby formed larger micrometer‐sized particles were formed, as shown in Figure 10c,d. This was due to the fact that TA and GFP formed hydrogen bonds efficiently in alkaline solutions and inefficiently in acidic solutions. Polyphenols could be used to stabilize Pickering emulsions. When bound to protein substances, the hydrophobic interactions and hydrogen bonding in the solution are enhanced, thus improving the stability of Pickering emulsions. For example, since zein, a proline‐rich water‐insoluble protein, is highly hydrophobic, it often brings about the formation of highly unstable emulsions. To address this limitation, researchers introduced TA onto the surface of corn alcohol‐soluble protein to reduce its hydrophobicity, making it easier to disperse in the continuous aqueous phase, and then improved the stability of Pickering emulsions.^[^ ^129^ ^]^ In addition, EGCG was also added to zein, and the surface of zein nanoparticles was modified by EGCG, increasing hydrogen bonding, reducing hydrophobicity, then the prepared zein‐EGCG composite particles exhibited excellent surface activity and higher interfacial stability.^[^ ^130^ ^]^

#### 4.2.2 Coatings and Capsules

Similar to the formation of capsules by polyphenol–metal combinations, polyphenol–protein combinations on sacrificial templates could form films, which subsequently form closed capsules after the template is removed. Han et al.^[^ ^131^ ^]^ choose charged and uncharged polyamino acids (i.e., poly(lysine) (PLys), poly(arginine) (PArg), poly(histidine) (PHis), poly(glutamic acid) (PGlu), poly(threonine) (PThr), poly(proline) (PPro), and poly alanine (PAla)) to reveal the intermolecular interactions between synthetic polypeptides and polyphenols (TA), as shown in Figure 10e. The pH‐responsive charge reversal behavior of synthetic polypeptide‐TA nanoparticles formed from polyamino acids with positively charged side chains was demonstrated through the study of molecular binding kinetics and thermodynamics. Furthermore, hydrogen bonds and electrostatic interactions in the solution contribute to the stability of the polyphenol–protein gel network. This suggested that the combination of polyphenols and polypeptides could form capsules with adjustable coating thickness and responsive behavior. As shown in Figure 10f–h, the PArg‐TA network capsules suggested higher cellular relevance compared to other synthetic polypeptide–polyphenol networks, facilitated intracellular drug delivery. This is possible due to positively charged peptides (arginine) possess higher cell binding and cell penetration activity. Consistent with the pH‐responsive characteristics of polyphenol–metal capsules, other studies have designed polyphenol–protein capsules based on the property that polyphenols contain multiple phenolic hydroxyl groups and are highly sensitive to hydrogen ion concentration in solutions. For example, under alkaline conditions, the oxidation of TA phenolic groups formed quinone intermediates, which reacted with amino or sulfhydryl groups of peptides, oxidatively coupling to the surface of corn protein particles to form a coating of DOX‐corn protein particles. Researchers utilized this pH‐responsive property to prepare TA‐zein DOX‐loaded capsules, allowing TA to be biodegraded in an acidic environment, thereby delivering DOX for cellular uptake.^[^ ^132^ ^]^ Environmental stimuli play a significant role in the formation of polyphenol–peptide coatings on templates. Another study had investigated a diverse library of peptides, polyphenols, and various regulators to elucidate their roles in the formation and regulation of polyphenol–peptide coatings. The results indicated that cationic peptides outperform anionic and neutral peptides in forming peptide–polyphenol coatings, as electrostatic interactions were the primary driving force for stable coating formation, followed by hydrogen bonding. Compared to other organic acids, citric acid was an effective regulator of cationic peptide–polyphenol interactions. This efficacy was attributed to citric acid regulating the electrostatic interactions between polyphenols and peptides by forming multiple hydrogen bonds.^[^ ^133^ ^]^ From the above overview of capsules and coatings, it is evident that electrostatic interactions play a crucial role in the formation of polyphenol–protein complexes.

#### 4.2.3 Hydrogel

**Figure 11:**
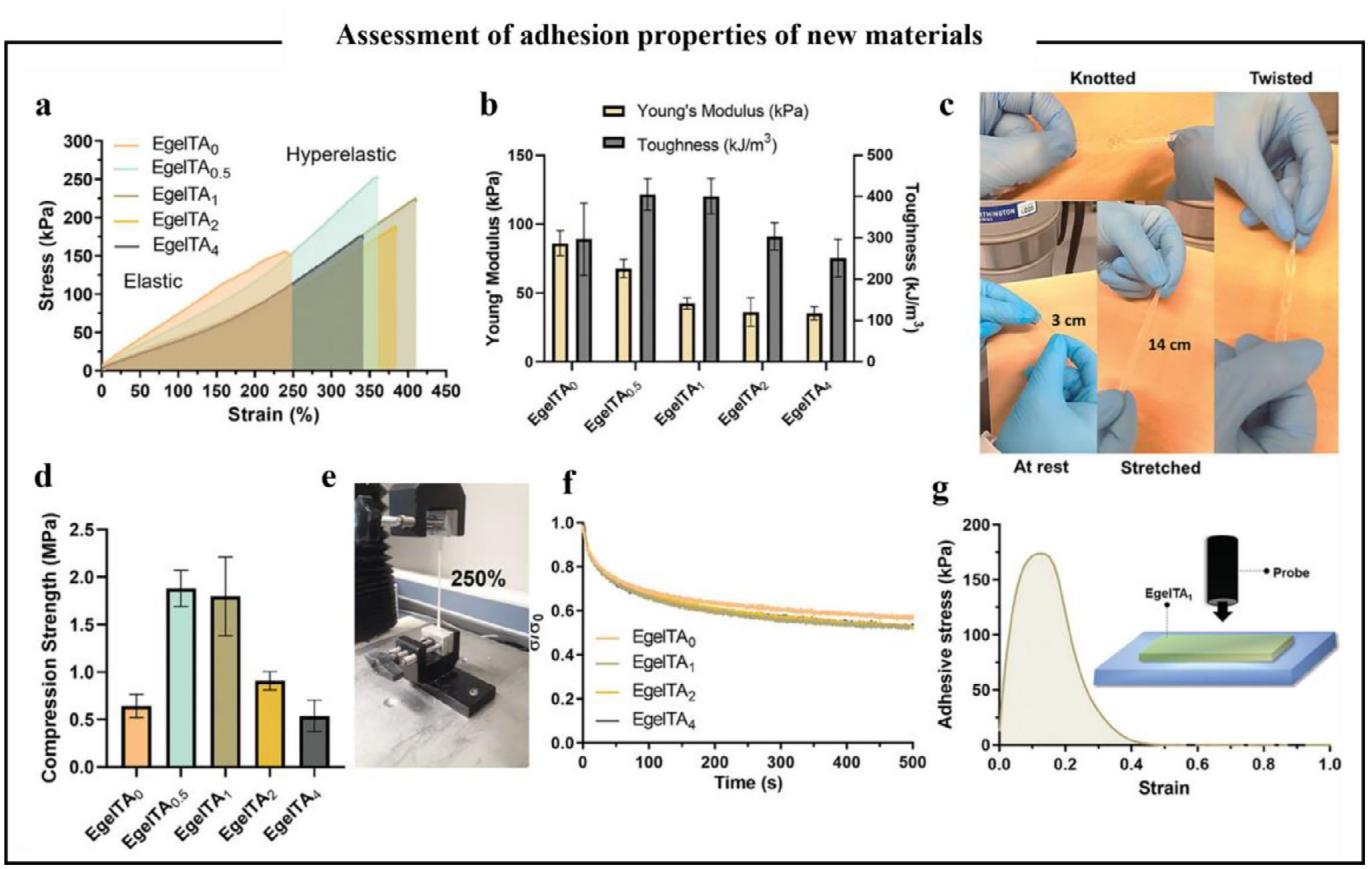
Assessment of adhesion properties of new materials synthesized with different concentrations of TA. a) Stress versus strain curves of eutectogels with different TA concentrations. b) Young's modulus versus toughness of the protein–elastomer eutectogels. c) Photos of EgelTA_1_ being stretched, knotted, and twisted. d) Compression strength of the protein elastomer eutectogels. e) Photo of EgelTA_1_ under 250% of strain. f) Stress–relaxation curves for eutectogels with different TA content. g) Adhesive stress versus strain curve for EgelTA_1_. Inset: Schematic drawing of the probe tack test used for adhesion measurements.^[^ ^135^ ^]^ Copyright 2024, Wiley.

Similar to the characteristics of polyphenol–metal hydrogels, pH significantly affects the gel properties of polyphenol–protein hydrogels. In acidic pH solutions, gels are barely observed. On the contrary, a higher proportion of larger aggregates formed during gelation of polyphenol–protein complexes was observed at pH 7 and 8.5, which indicated that neutral or alkaline environments promote the formation of a gel network by polyphenol–protein complexes.^[^ ^134^ ^]^ At this point, hydrogen bonding is the key factor in the gel formation of polyphenol–protein complexes. Polyphenols, as multifunctional substances, bind to proteins, regulating their structural conformation, enhancing the network, and transforming the elastic behavior of eutectic gels into super‐elasticity. A previous study had reported a dynamic cross‐linking of TA with protein to form eutectogels (EgelTA_x_) gelatin patches. As shown in **Figure** **11**, the experimental results revealed that the addition of TA conferred the EgelTA_1_ eutectic gel had good elasticity, high phase transition temperature, high self‐adhesion, and good ionic conductivity. This was attributed to the high density of hydroxyl groups and strong hydrogen bonding capacity in TA.^[^ ^135^ ^]^ Additionally, EGCG has been used to regulate the structure of honeybee pupa protein (HBPP), thereby enhancing the physicochemical properties of HBPP gels. Among the HBPP‐EGCG gels evaluated, those with ≥2% EGCG addition showed the best printing performance. This was believed to be due to the addition of EGCG that enhanced the hydrogen bonding interactions of HBPP, while weakening hydrophobic interactions, thereby inducing HBPP aggregation behavior and forming a stronger gel network.^[^ ^136^ ^]^ As evidenced by the content on polyphenol–protein‐formed hydrogels, stronger hydrogen bonding interactions enhanced the gel properties of hydrogels.

### 4.3 Self‐Assembly of Polyphenols with Polysaccharides

Polysaccharides are biomolecules widely found in plants, algae,^[^ ^137^ ^]^ and microorganisms, and are often used as functional additives due to their thermal stability, water retention properties, biocompatibility, and low toxicity. When combining polyphenols with polysaccharides to form functional materials, the various functional groups in polysaccharides (such as hydroxyl groups as hydrogen bond donors, the distribution of π electrons within specific structural domains, and hydrophobic regions, etc.) interact with the groups of polyphenols, through various intermolecular interactions, driving the ordered assembly and stabilization of polysaccharide–polyphenol composites.^[^ ^138^ ^]^ Moreover, covalent interactions between polysaccharides and polyphenols mainly include 1) Dehydration condensation of polysaccharide hydroxyl groups with polyphenol carboxyl groups, 2) Nucleophilic substitution of hydroxyl groups under alkaline conditions, 3) Condensation of polysaccharide reducing ends with polyphenol phenolic hydroxyl groups, and 4) Michael addition reactions between polysaccharide double bonds and quinone structures generated by polyphenol oxidation.^[^ ^139^ ^]^ Polyphenols and polysaccharides spontaneously form polyphenol–polysaccharide complexes, exhibiting multiple functional characteristics such as antibacterial properties, pH responsiveness, high viscosity, and good mechanical strength (**Table** **3**). The following sections primarily outline the interactions between polyphenols and starch, fiber, and chitin to improve the physicochemical properties of polysaccharides, thereby enhancing the functional characteristics of polyphenol–polysaccharide complexes.

**Table 3:** Different structures of polyphenol–polysaccharides.

Phenolic ligands	Polysaccharides	Structure	Main interaction	Functional characteristics	Refs.
Ferulic acid	Corn starch	Nanoparticle	Hydrogen bonding	Relieve postprandial blood sugar levels	[141]
Lonicera caerulea berry polyphenols	Wheat starch	Nanoparticle	Hydrogen bonding	Relieve postprandial blood sugar levels	[142]
Lingonberry polyphenols	Corn starch	Nanoparticle	Hydrogen bonding	Relieve postprandial blood sugar levels	[143]
Anthocyanin‐3‐ o ‐glucoside (Cy3Glc)	Different citrus pectin polysaccharide fractions	Nanoparticle	Hydrogen bonding, electrostatic effect, hydrophobic interaction	/	[145]
TA	Pectin and carboxymethyl chitosan	Film	Hydrogen bonding	Antibacterial, color protection, and UV/visible light protection	[146]
Quercetin	Chitin	Hydrogel	Hydrogen bonding, hydrophobic interaction, and electrostatic effect	Excellent biocompatibility, excellent photothermal and electrochemical properties	[149]
TA	Chitin	Hydrogel	Hydrogen bonding	Compressive strength	[150]
TA, EGCG	Chitin	Film	Hydrogen bonding	Antibacterial, free radical scavenging, and increased tensile strength	[151]

#### 4.3.1 Starch

Nanoparticles formed by polyphenols and starch could reduce starch digestion. Rapid digestion of starch leads to elevated postprandial blood glucose levels, and long‐term sustained elevated postprandial blood glucose induces a variety of chronic diseases, including cardiovascular disease and hyperglycemia.^[^ ^140^ ^]^ Researchers have developed a composite of corn starch and ferulic acid, and the results showed that the nanoparticle had a much greater effect on starch digestibility than ferulic acid alone.^[^ ^141^ ^]^ Additionally, a composite of wheat starch and Japanese honeysuckle berry polyphenols formed through high‐pressure processing also reduced starch digestibility, thereby lowering postprandial blood glucose levels.^[^ ^142^ ^]^ Similarly, the nano‐composite formed by lingonberry polyphenols and corn starch also reduced the digestibility of corn starch.^[^ ^143^ ^]^ These studies indicated that during starch gelatinization, the non‐covalent interactions formed between polyphenols and starch disrupted the short‐range ordered structure of starch, altered the crystal structure, and increased starch viscosity and viscoelasticity. Furthermore, polyphenols could inhibit the activity of α‐amylase and α‐glucosidase, thereby reducing starch digestion.

#### 4.3.2 Cellulose

Pectin is an anionic polysaccharide composed mainly of galacturonic acid (GalA) and is a water‐soluble fiber.^[^ ^144^ ^]^ A recent study had emphasized that charge interactions, hydrogen bonds, hydrophobic effects, and anthocyanin‐3‐O‐glucoside (Cy3Glc) stacking contribute to the binding of polyphenols to pectin.^[^ ^145^ ^]^ When polyphenols and polysaccharides interact, they are also affected by the solution environment (polyphenol concentration, polysaccharide type). Research has shown that pectin polysaccharides with less branched arabinoglycans exhibited higher affinity for proanthocyanidins and formed more insoluble aggregates. In contrast, pectin polysaccharides with arabinose glycosides covalently bound to polyphenols exhibit lower affinity for proanthocyanidins.^[^ ^138^ ^]^ As mentioned in the previous overview, the addition of polyphenols endowed other materials with multifunctional properties. For example, polyphenols strengthened the network structure between pectin and carboxymethyl chitosan through intermolecular hydrogen bonds. This not only enhanced the toughness and corrosion resistance of pectin and carboxymethyl chitosan films, but also gave the films stronger coloring and UV and visible light protection.^[^ ^146^ ^]^ The abundant ─COO─ and ─OH groups in TA facilitated hydrogen bonding and electrostatic interactions with carboxymethyl cellulose, as well as photothermal properties, thereby conferred pH responsiveness and photoelectric responsiveness to the material, as well as free radical scavenging, thus enhancing the antioxidant, anti‐inflammatory, and antibacterial properties of hydrogels.^[^ ^147^ ^]^

#### 4.3.3 Chitin

The addition of polyphenols alters the degree of polymerization and rheological properties of hydrogels. This is because phenolic compounds have hydrogen bonding capabilities, and the more hydrogen bonding groups, the higher the degree of aggregation.^[^ ^148^ ^]^ Consistent with Sections 4.1 and 4.2, the addition of polyphenols could enhance the functional properties of hydrogels. A previous study had described a strategy to fabricate high‐performance and multifunctional chitin‐based composite hydrogels using quercetin. It was found that quercetin mediated non‐covalent interactions (hydrogen bonding, ionic bonding, and hydrophobic interactions) between chitin chains and enhanced the mechanical properties, biocompatibility, photothermal, and electrochemical properties of chitin hydrogels.^[^ ^149^ ^]^ Furthermore, non‐covalent cross‐linking between polyphenols and chitosan reduced the self‐assembly of chitosan molecules, thereby enhancing the compressive strength of chitosan hydrogels. In this study, various polyphenols, including TA, GA, pyrogallic acid, quercetin, and protocatechuic acid, were used to form hydrogels with chitosan. At optimal concentrations, all of these polyphenols improved the compressive strength of the chitosan hydrogels.^[^ ^150^ ^]^ In addition, polyphenols (EGCG and TA) interacted with chitin to form hydrogen bond networks in the film, which could increase the tensile properties, free radical scavenging, and antibacterial activity of the film material.^[^ ^151^ ^]^

### 4.4 Self‐Assembly of Polyphenols with Other Substances

#### 4.4.1 Nucleic Acid

**Figure 12:**
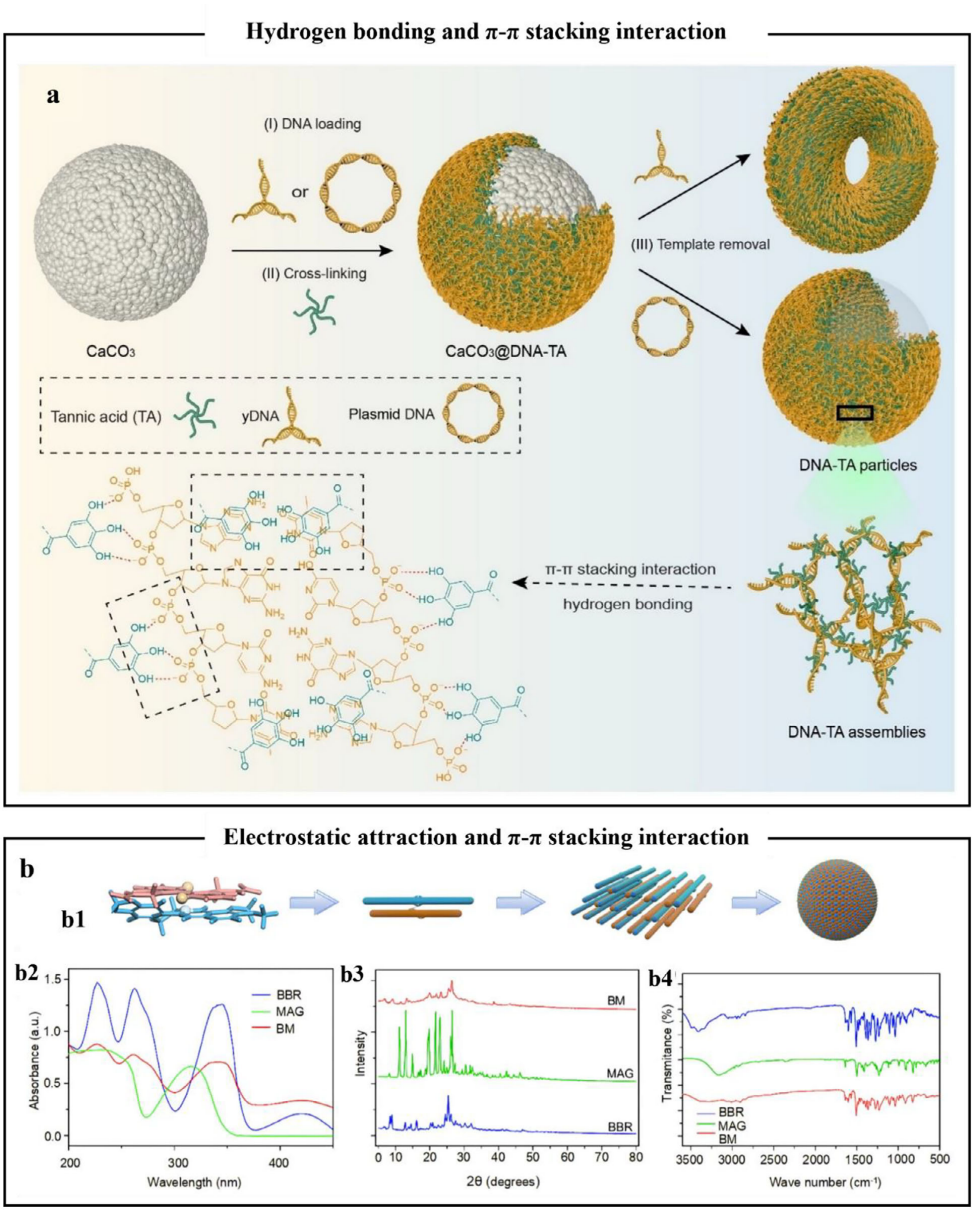
Polyphenols interact with other bioactive substances to self‐assemble to form delivery systems. a) Schematic representation of the assembly of DNA‐TA particles: (I) loading of CaCO_3_ particles with DNA (yDNA or plasmid DNA); (II) cross‐linking by TA; and (III) removal of CaCO_3_ template core to yield DNA‐TA particles.^[^ ^154^ ^]^ Copyright 2023, Wiley. b) Stable spherical nanoparticles (BM) formed by self‐assembly of berberine (BBR) and magnolol (MAG) were formed by electrostatic attraction and π–π stacking. (b1) Self‐assembling mode between BBR and MAG in BM. (b2) UV–vis spectra of BBR, MAG, and BM. (b3) XRD spectra of BBR, MAG, and BM; (b4) FTIR spectra of BBR, MAG, and BM.^[^ ^162^ ^]^ Copyright 2024, BMC.

As a key biological macromolecule, nucleic acid is known for its unique structure and programmability, which is mainly divided into deoxyribonucleic acid (DNA) and ribonucleic acid (RNA). Specifically, DNA and RNA materials not only provide innovative solutions for drug delivery, but also play a key role in cutting‐edge technologies such as molecular diagnostics,^[^ ^152^ ^]^ gene editing,^[^ ^153^ ^]^ and immunotherapy.^[^ ^154^ ^]^ Polyphenols, as versatile assembly materials, form carrier drugs when combined with nucleic acids, offering unique advantages in the development of gene and vaccine delivery platforms. Zhang et al.^[^ ^155^ ^]^ described a pH‐responsive TA conjugated with a tetrahedral framework nucleic acid as a transdermal RNAi drug for the treatment of psoriasis. The system dynamically regulates loading and release through hydrogen bonding for efficient silencing of skin inflammatory factors. Qu et al.^[^ ^154^ ^]^ developed a DNA‐polyphenol microsphere prepared through supramolecular assembly of TA with DNA (**Figure** **12**a). This system stabilized its structure through hydrogen bonding and π–π stacking, enabled simultaneous delivery of ovalbumin antigen and nucleic acid adjuvant, activation of macrophages, induction of efficient immune responses, and resistance to nucleases degradation. Zhu et al.^[^ ^156^ ^]^ developed polyphenolic TA‐mediated DNA smart nanocarriers, which formed a 3D network skeleton through hydrogen bonding to achieve intracellular cascade‐responsive drug release in tumor cells. It was evident that polyphenols played a pivotal role in the preparation of smart responsive carriers. Moreover, pH responsiveness was the most intriguing functional property of polyphenols in functional materials.

#### 4.4.2 Multiple Polyphenols

The chemical structure of polyphenolic compounds is centrally characterized by a phenolic hydroxyl‐substituted benzene or thick ring skeleton. This structure confers antioxidant activity, anti‐inflammatory, antitumor, bacteriostatic, metabolic, and immunomodulatory functions.^[^ ^157^ ^]^ Resveratrol is a natural polyphenolic compound with multiple biological activities, including antioxidant, anti‐inflammatory, lipid‐regulating, and cardiovascular protective properties. In a previous study, researchers utilized the high antioxidant capacity of resveratrol and proanthocyanidins to design stable nanoparticles through hydrogen bonding. Due to the polyhydroxy and charge imbalance characteristics of resveratrol and proanthocyanidins, the nanoparticles were grafted with matrix metalloproteinases‐targeted peptides, thereby enhancing the nanoparticles’ ability to target damaged myocardial tissue. Additionally, the synergistic antioxidant and anti‐inflammatory properties of the two phenols, combined with the nanoparticles, alleviated inflammation and apoptosis damage in mouse myocardial cells induced by ischemia–reperfusion.^[^ ^157^ ^]^ Curcumin is a type of polyphenol compound containing various functional groups such as phenolic hydroxyl groups, ketone groups, and enol hydroxyl groups. These properties conferred it with strong free radical scavenging capacity, cardiovascular protective effects, and other biological activities. Additionally, it is sensitive to light, heat, and iron ions.^[^ ^158^ ^]^ Huang et al.^[^ ^159^ ^]^ fabricated a patch based on self‐assembled nanoparticles of curcumin and TA composite with a gelatin methacryloyl hydrogel. The patch could repair and prevent radiation dermatitis by integrating the antioxidant and photothermal response properties of curcumin/tannic acid nanoparticles with the physical shielding function of gelatin methacryloyl hydrogel.

#### 4.4.3 Alkaloid

Alkaloids are a class of natural compounds primarily containing basic nitrogen atoms, exhibiting a wide range of pharmacological activities, including antimalarial, anticancer, antibacterial, and antidiabetic activities etc.^[^ ^160^ ^]^ In this section, the interactions between berberine and polyphenols are the main focus of discussion. The positively charged quaternary ammonium ions are the most critical structural components in the self‐assembly process between polyphenols and berberine.^[^ ^161^ ^]^ This is because the positively charged berberine could attract negatively charged groups through electrostatic interactions, making it the most important step in its self‐assembly with polyphenols. Xu et al.^[^ ^162^ ^]^ developed the formation of stable spherical nanoparticles based on self‐assembly of berberine and magnolol, in which electrostatic interactions (deprotonation of phenolic hydroxyl groups combined with quaternary ammonium salts) and π–π stacking enhance their spatial structure (Figure 12b). These nanoparticles could synergistically exert anti‐inflammatory effects, repair the intestinal barrier, and regulate the microbiota, demonstrating superior efficacy and safety compared to traditional drugs in an ulcerative colitis model. Fu et al.^[^ ^95^ ^]^ constructed based on berberine (BBR) and chlorogenic acid (CGA) using molecular self‐assembly technology. The electrostatic interactions between the quaternary ammonium structure of BBR (positively charged N) and the carboxylic acid portion of CGA primarily contribute to the stability of the nanoparticles, followed by hydrogen bonding or π–π conjugation effects. Additionally, BBR and CGA exhibited synergistic effects, achieved efficient antibacterial performance, accelerated wound healing, and reduced inflammatory responses. Based on the pH responsiveness of polyphenols, controlled release was achieved, and the nanoparticles demonstrated good biosafety and tissue compatibility (**Table** **4**).

**Table 4:** Self‐assembly of polyphenols and other substances.

Phenolic ligands	Other substances	Structure	Main interaction	Functional characteristics	Refs.
TA	Tetrahedral framework nucleic acid	Nanoparticle	Hydrogen bonding	pH response, anti‐inflammatory	[155]
TA	DNA	Capsule	Hydrogen bonding, π–π stacking	Efficient delivery	[156]
Resveratrol	Proanthocyanidin	Nanoparticle	Hydrogen bonding	Antioxidant	[157]
TA	Curcumin	Hydrogel	Hydrogen bonding, metal coordination bond	Antioxidant, anti‐inflammatory, excellent photothermal and electrochemical properties	[159]
Magnolol	Berberine	Nanoparticle	Electrostatic effect, π–π stacking	Antioxidant, anti‐inflammatory	[162]
Chlorogenic acid	Berberine	Nanoparticle	Hydrogen bonding, π–π stacking	Antioxidant, anti‐inflammatory, antimicrobial	[95]

## 5 Functional Contribution of Polyphenols in Biomedicine

Polyphenolic compounds exhibit multidimensional application value in biopharmaceutical delivery due to their unique chemical structures and biological activities. The abundant phenolic hydroxyl groups in their molecules form stable complexes with biomolecules or nanocarriers through non‐covalent and covalent interactions, which could significantly enhance the efficiency and targeting of drug delivery. thus, in this section, the biomedical applications of polyphenols will be detailed in terms of drug delivery, bioreactor simulation, and biomaterials.

### 5.1 Bioreactors

#### 5.1.1 Mimetic Organelles

**Figure 13:**
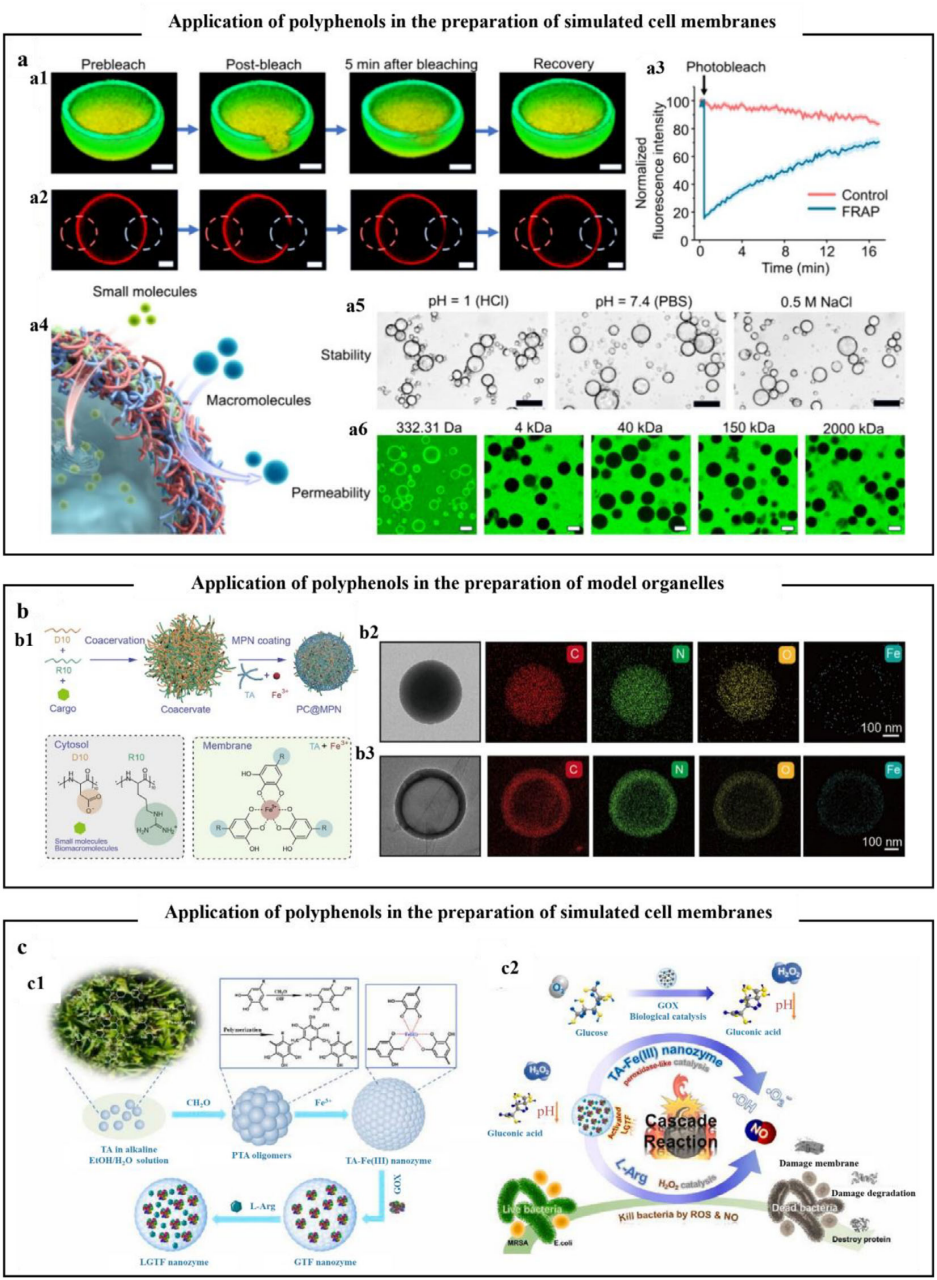
Polyphenols in organelles, cell membranes, and microreactors. a) Stable fluidic membranes on FMPs to allow cascade reactions. (a1) FRAP measurements of membrane fluidity using time‐lapse CLSM 3D reconstruction depth coding images. Scale bar, 10 µm. (a2) 2D CLSM images of membrane fluidity. Scale bar, 10 µm. (a3) Time‐dependent membrane fluorescence intensity measurements for assessing membrane fluidity. (a4) Schematic illustration of the semi‐permeability of the membrane. (a5) Stable FMPs configurations in HCl, PBS, and saline solutions. Scale bar, 50 µm. (a6) CLSM images of permeability studies showing that small molecules (fluorescein) penetrated the interior, while large FITC‐dextran molecules (4–2000 kDa) were impermeable. Scale bar, 25 µm.^[^ ^165^ ^]^ Copyright 2024, American Chemical Society. b) Design and fabrication of PC@MPN membrane‐bound protocells. (b1) Schematic illustration of the formation of PC@MPN. D10, polyAsp; R10, polyArg. (b2) TEM and EDX elemental mapping of PC@MPNs. (b3) TEM and EDX elemental mapping of PC@MPNs after core removal using dimethylformamide.^[^ ^166^ ^]^ Copyright 2023, American Chemical Society. (c1) Synthetic process of LGTF nanozyme. (c2) Illustration of the glucose‐activated dual‐cascade reaction mechanism for enhanced synergistic bacteria‐infected wound healing.^[^ ^169^ ^]^ Copyright 2024, Elsevier.

As the basic structural and biological units of life, cells can easily lose functional activity or die in vitro due to their inherent complexity and fragility. To overcome these problems, researchers have constructed several types of artificial cells that mimic natural cells.^[^ ^163^ ^]^ In recent studies, cohesive droplets formed by liquid–liquid phase separation have been used for the construction of cells that mimic natural cells. However, their presence lacked selective molecular uptake and release, and limited membrane fluidity.^[^ ^164^ ^]^ To improve the fluidity and stability of the mimetic cellular structures, the researchers constructed a stable, flexible, and functionally scalable fluidic membrane‐bound protoplast based on the dynamic reorganization of phenolic hydroxyl groups in polyphenols via hydrogen bonding, which balanced structural stability and dynamic responsiveness through a network of hydrogen bonds. Liquid–liquid phase separation of TA and polyethylene glycol was explored for the formation of coalescing droplets. Upon introduction of polyvinylpyrrolidone molecules, a dense hydrogen‐bonding network spontaneously formed on the surface of the coalescing droplets, resulting in a strong fluid‐membrane‐bound progenitor cell (FMP). The membrane fluidity, stability, and permeability of FMP were investigated by photobleaching fluorescence recovery, permeability experiments, and other research methods. The results showed that membrane fluidity allowed fluorescence recovery to 70% of the original value within 16 min after photobleaching (**Figure** **13**a1, a2, a3). These FMPs were stable in various neutral and acidic solutions (hydrochloric acid at pH 1, PBS at pH 7.4, and 0.5 M NaCl brine) (Figure 13a4,a5). Moreover, the permeability of the FMP membranes was related to molecular size (Figure 13a6), which provided a platform for regulating chemical reactions by designing different molecular diffusion routes across the membrane.^[^ ^165^ ^]^

#### 5.1.2 Simulated Cell Membranes

Conventional membrane‐free copolymers are prone to fusion due to the lack of stability and cannot mimic the semipermeable membrane properties of real cells. Existing lipid or polymer membranes could provide stability but suffer from complex preparation and environmental sensitivity. MPN is formed by self‐assembly of metal ions and polyphenols through ligand bonding, which is suitable for use as a biomimetic membrane material because of its high biocompatibility, antioxidant properties, and tunable permeability. Currently, a study reported a peptide‐based amphiphilic copolymer‐encapsulated protoplast, and a biomimetic model with cytoprotective function was constructed by combining MPN membranes with peptide‐based copolymer cytoplasm (PC@MPNs) (Figure 13b1). Oligopeptides with opposite charges acted as molecularly crowded cytoplasmic sols and MPN coatings as membranes. It was further confirmed by TEM images and energy dispersive X‐ray spectroscopy elemental mapping that PC@MPN consists of C, O, N, and Fe (Figure 13b2). Notably, removal of the peptide‐based copolymer inner core yielded the MPN shell, confirming that the MPN membrane was a separate entity from the peptide‐based copolymer cytoplasm (Figure 13b3). Additionally, a glucose‐driven cascade reaction was utilized to demonstrate the ability of PC@MPNs to segregate biomolecular transformations and preserve loaded molecules.^[^ ^166^ ^]^ Metal–phenol synergizes with other substances to synthesize biofilms that are biocompatible and stable, encapsulating and separating biomolecules from the external environment. Their modular design supports customization of membrane properties, load types, and surface modifications, providing a novel platform for synthetic biology, drug delivery, and disease treatment.

#### 5.1.3 Biomimetic Nano enzyme

Natural enzymes are characterized by high catalytic efficiency and high substrate affinity and are therefore widely used in biological fields such as disease diagnosis and cancer therapy.^[^ ^167^ ^]^ However, the disadvantages of high cost, low stability, and easy inactivation limit their practical application in harsh environments. Nanozyme, as enzyme substitutes with not only enzyme‐like activity but also high stability, tunable catalytic performance, and high pH/temperature tolerance, has been intensively investigated for the establishment of biomimetic catalytic systems.^[^ ^168^ ^]^ Microenvironment‐triggered, metal–polyphenol‐loaded nanozymes for ROS/NO synergistic hyperglycemic wound healing. An enzyme‐responsive nanozyme system was designed by combining metal polyphenols (TA‐Fe) with natural enzymes (GOx) (Figure 13c1). Based on the mechanism where GOx consumes high concentrations of glucose while generating reactive oxygen species (ROS) and nitric oxide (NO). Glucose consumption and gluconic acid production lowered glucose levels to promote wound healing and lowered the pH of the wound microenvironment to enhance the catalytic activity of the LGTF nanozyme system (Figure 13c2).^[^ ^169^ ^]^

### 5.2 Biomaterials

#### 5.2.1 Biosensor

Biosensors are portable analytical devices used for the rapid identification of biological agents such as pathogens, proteins, glucose, and blood pressure.^[^ ^170^ ^]^ Biosensors are moving toward the simultaneous identification and analysis of multiple analytes. Polyphenol molecules have become important functional materials for the construction of high‐performance biosensors due to their abundant phenolic hydroxyl groups, bacteriostatic, and antioxidant effects. During recent years, the strategy of constructing dense functionalized membranes on the surface of metal, carbon‐based, and ceramic electrodes by taking advantage of the self‐polymerization and cross‐linking properties of polyphenols has been widely used in the preparation of biosensor platforms.^[^ ^171^ ^]^

**Figure 14:**
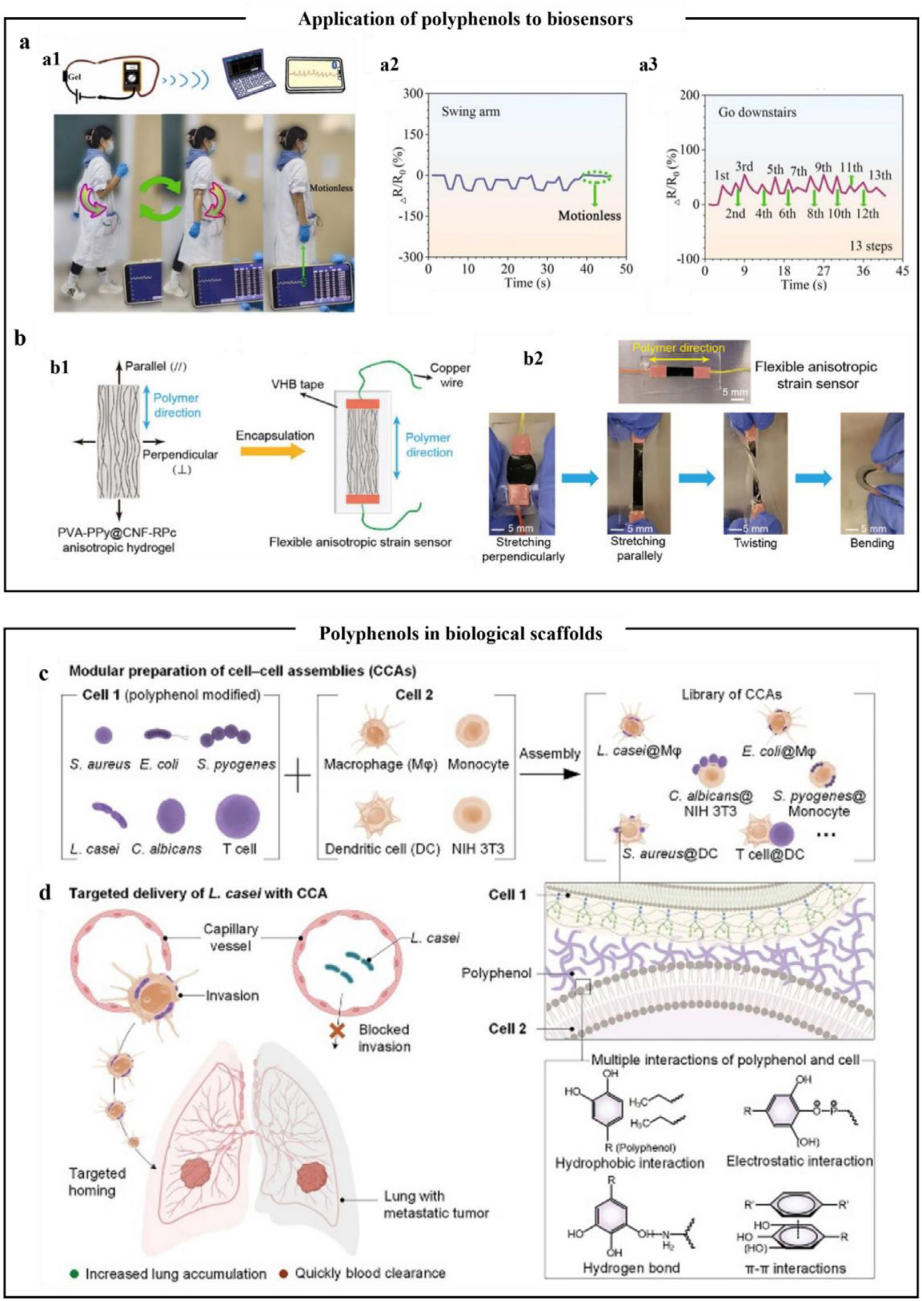
Polyphenols in biosensors, bone healing, and biological scaffolds. a) Wireless strain sensor based on CMM gel: (a1) Gel connected with Bluetooth device; The schematic diagram of the swing arm movement. (a2) Relative resistance changes during the process of arm flexion. (a3) Wireless monitoring of real‐time resistance changes during stair descent (13 steps).^[^ ^172^ ^]^ Copyright 2024, Elsevier. b) Tensile properties of PVA‐PPy@CNF‐RPc hydrogel. (b1) Schematic design of anisotropic PVA‐PPy@CNF5‐RPc100 hydrogel strain sensor. (b2) The obtained hydrogel sensor has good flexibility, stretchability, and elastic reversibility.^[^ ^173^ ^]^ Copyright 2024, Wiley. c) Modular construction of cell–cell assemblies (CCAs) to enhance intercellular cooperation for lung metastasis immunotherapy. d) Facile assembly of CCAs with polyphenol‐modified cells (cell 1) and other cells (cell 2). Multiple intermolecular interactions, such as hydrophobic interactions, electrostatic interactions, hydrogen bonds, and π–π interactions, derived from polyphenols, trigger CCA formation. d) Mechanism of *L. casei*@Mφ targeting accumulated in the lung. Mφ in *L. casei*@Mφ showed the capability to tumor homing, which provides the driving force for targeted delivery of *L. casei*.^[^ ^179^ ^]^ Copyright 2024, Wiley.

In practical applications, gel sensors are in urgent need of recyclable gels with excellent mechanical properties and outstanding self‐healing ability due to their undesirable stretchability. For example, an investigation reported a novel versatile gel in which TA built a 3D network structure through hydrogen bonding, electrostatic interaction, and hydrophobic interaction to enhance the cross‐linking property of the gel. Due to the reversible cross‐linking formed by electrostatic interaction and hydrogen bonding, the gel showed excellent mechanical properties, self‐healing, and recyclability. The gel‐based sensors were widely used for healthcare detection, such as fine motion, breathing, and handwriting (**Figure** **14**a).^[^ ^172^ ^]^ In addition, conventional conductive sensors usually require cumbersome salting‐out treatments or cross‐linking of biotoxic heavy metal ions to immobilize the ordered structure and achieve ionic conductivity, which made the prepared anisotropic hydrogel sensors unsuitable for implantable electronics due to the risk of ion diffusion in the organism. A previous research reported a combination of pre‐stretching and TA cross‐linking strategies with hydrogen bonding to immobilize the anisotropic structure after pre‐stretching to develop an anisotropic conductive hydrogel (PVA‐PPy@CNF‐RPc) with excellent mechanical properties, orientation‐dependent conductivity, and biocompatibility (Figure 14b).^[^ ^173^ ^]^ The material displayed great promise in the field of wearable devices, smart robots, and implantable bioelectronics, and provided a new design paradigm for the next generation of flexible electronic devices. With the continuous progress of nanomaterials and intelligent response systems, polyphenol‐based biosensors are expected to realize multimodal joint detection and real‐time dynamic monitoring, which would further promote the development of precision medicine and in vitro diagnostic technology.

#### 5.2.2 Healing of Bone

Bone healing is a dynamic, multistep physiological process that relies on cellular collaboration and precise molecular regulation. Polyphenols are potential candidates for improving bone defect repair due to their dual antioxidant, anti‐inflammatory, and osteogenic/osteoclastic regulatory properties.^[^ ^174^ ^]^ However, for the present, clinical applications for bone healing need to incorporate material science and individualized medical strategies to overcome challenges such as low bioavailability and complex mechanisms of action. Conventional artificial ligaments are prone to fiber encapsulation and poor bone healing due to a lack of osteogenic bioactivity. One study had reported the successful development of a functionalized coating material (PGPH) based on polyimide fiber (PIF) fibers by constructing polyphenol–metal network (PMN) as a surface coating for PIF artificial ligaments. In vitro and in vivo studies confirmed that PGPH could effectively inhibit fiber encapsulation, promote bone regeneration, enhance its anti‐inflammatory and antimicrobial functions, and foster ligament‐bone healing.^[^ ^175^ ^]^ Tea polyphenol (TP) nanoparticles (NPs), a nature‐inspired antioxidant in combination with 2‐(4‐Carboxyphenyl)‐4,4,5,5‐tetramethylimidazoline‐1‐oxyl‐3‐oxide (carboxy‐PTIO), a NO scavenger, could provide maximized positive therapeutic effects on osteoarthritis by eradicating both ROS and NO.^[^ ^176^ ^]^ In summary, polyphenol‐modified biomaterials not only provide scaffolding and guidance for bone healing but also achieve “smart” repair of bone tissue regeneration through the modulation of cell signaling and improvement of the microenvironment, and this strategy provides a novel and effective therapeutic platform for future clinical bone repair.

#### 5.2.3 Biological Scaffold

A biological scaffold is a three‐position support structure for tissue repair or regeneration, usually made of biodegradable or biocompatible materials, which provides mechanical support to target tissues for repair and regeneration of bone, cartilage, vascular fields, and others. Polyphenols themselves have excellent biocompatibility, which could reduce immune rejection after scaffold implantation. Meanwhile, their antioxidant properties scavenge free radicals and inhibit inflammatory responses, while their antimicrobial properties reduce the risk of infection and provide a favorable microenvironment for tissue repair.^[^ ^177^ ^]^ For example, MPN‐modified bone scaffolds significantly promoted osteogenic differentiation by scavenging ROS and releasing Sr^2^⁺ ions.^[^ ^178^ ^]^ Polyphenols responded to the scaffolds through reversible dynamic coordination (with metal ion binding) to achieve responsive degradation and self‐repair of scaffolds. For example, the MPN network constructed by iron ions and TA modulates intercellular interactions and achieves multicellular self‐assembly (Figure 14c).^[^ ^179^ ^]^ In particular, the study highlights the specific application of cell–cell assemblies in combination with *Lactobacillus casei and Macrophages* (*L. casei*@Mφ) in enhancing cancer immunotherapy. Following intravesical administration, *L. casei*@Mφ allowed chemotactic Mφ to promote *L. casei* accumulation in tumors. Accumulated *L. casei* polarizes tumor‐associated Mφ toward a pro‐inflammatory phenotype, significantly improving the immunotherapeutic response, slowing tumor progression, and attenuating lung metastasis (Figure 14d). In contrast, vascular scaffolds in which TA was combined with thrombin inhibitors achieve synergistic regulation of anticoagulant‐promoter endothelial function through dynamic chemical bonding.^[^ ^180^ ^]^ Through chemical cross‐linking, integration of bioactive molecules, and dynamic functional regulation, polyphenols have emerged as an ideal material for the construction of multifunctional biological scaffolds, which were widely used in trauma repair and bone regeneration.

#### 5.2.4 Wound Healing and Hemostasis

The materials used for wound treatment are categorized by modality into dressings and sutures. Dressings are designed to protect the wound from secondary injuries, maintain moisture at the wound site, and prevent infection, among other things.^[^ ^181^ ^]^ Sutures are made of natural or synthetic materials and are intended to promote wound healing, reduce bleeding and risk of infection, and provide the mechanical support necessary for wound repair.^[^ ^182^ ^]^ Conventional wound healing materials mainly rely on passive physical protection, lack of bioactivity, insufficient antimicrobial properties, mechanical and functional limitations, and difficulties in striking an ideal balance between the rate of biodegradation and the biocompatibility of the degradation products, which might trigger localized inflammation or other undesirable reactions.^[^ ^183^ ^]^

**Figure 15:**
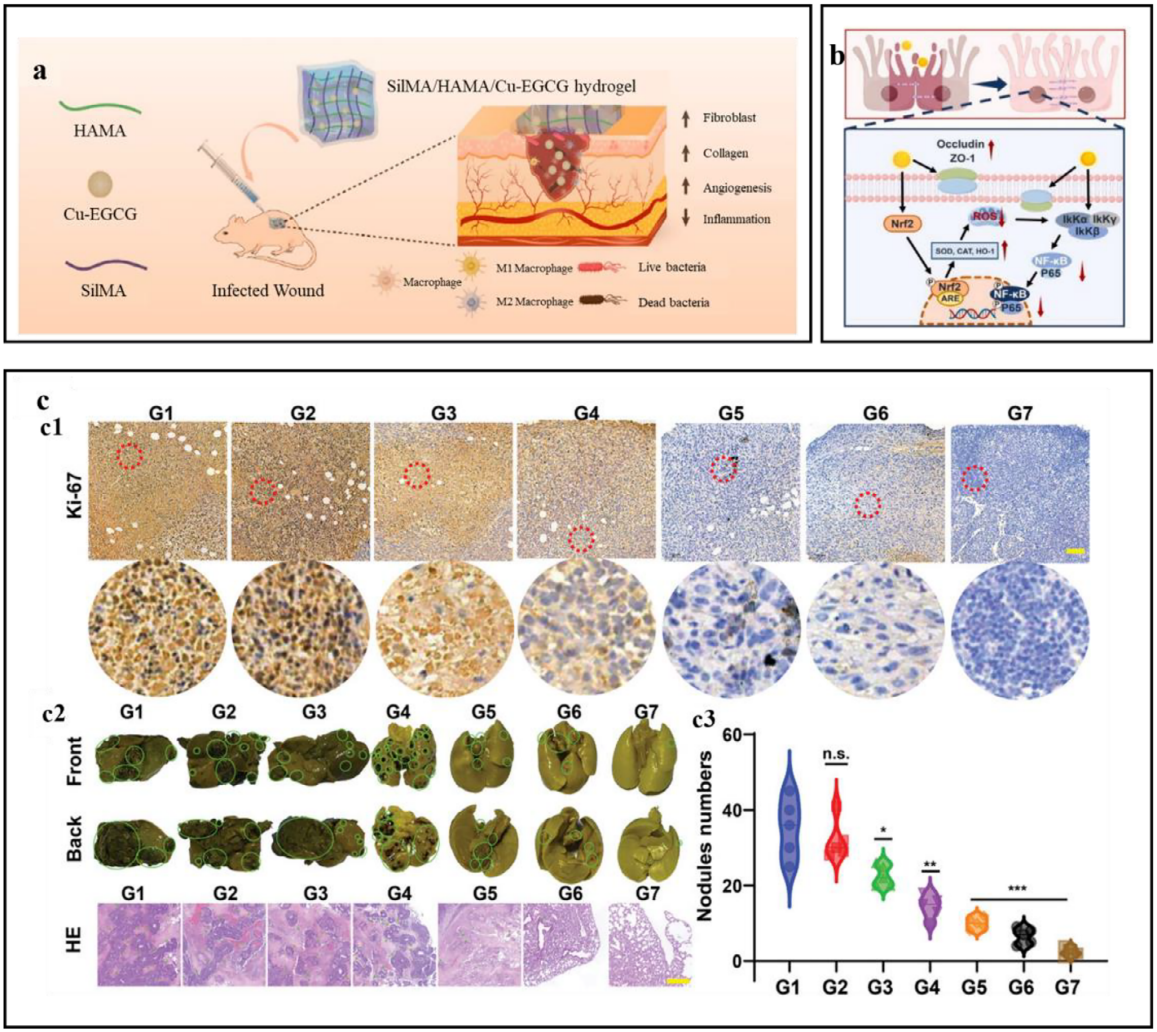
a) SilMA/HAMA/Cu‐EGCG hydrogel in infected wound healing and skin reconstruction.^[^ ^184^ ^]^ Copyright 2023, Elsevier. b) Signaling pathway mechanism diagram of CuL NCs for the treatment of colitis.^[^ ^191^ ^]^ Copyright 2024, Elsevier. c) Inhibition of tumor metastasis after surgery. c1) Immunohistochemistry showing expression of Ki‐67 in the tumor tissues; Scale bars, 100 µm. c2) Digital photo and documentation of B16F10 cells metastasis into lungs stained with Bouin's reagent and H&E images of different groups; Scale bars, 500 µm. Images are representative photographs from five biologically independent replicates. c3) Pulmonary metastatic nodules determined in individual groups: G1: control, G2: surgery, G3: surgery + GelTA, G4: surgery + GelTAMNPs, G5: surgery + PD‐1, G6: surgery + GelTAMNPs + AMF, and G7: surgery + GelTAMNPs + AMF + PD‐1.^[^ ^193^ ^]^ Copyright 2024, Wiley.

Polyphenol‐based biomaterials have shown great potential in the field of wound healing due to their unique chemical structure and multiple bioactivities, which not only endow the materials with excellent antioxidant and anti‐inflammatory effects but also cross‐link with plasma proteins rapidly to promote blood coagulation and clot formation, thus realizing highly efficient hemostasis in the early stage of wounding. Meanwhile, polyphenol coatings improve the local microenvironment of the wound, modulate the inflammatory response, and activate cell migration and proliferation, accelerating re‐epithelialization and tissue regeneration.^[^ ^24^ ^]^ A previous research had proposed a new hydrogel dressing that combined high strength, anti‐swelling, antimicrobial, and antioxidant capabilities. The hydrogel was mechanically supported by a dual network structure composed of polyvinyl alcohol and agarose, with hyperbranched polylysine, a highly effective antimicrobial cationic polymer, and TA, a strong antioxidant molecule, as functional components. The TA enhances the antioxidant capacity of the dressing and synergizes with hyperbranched polylysine to enhance the antimicrobial capacity.^[^ ^24^ ^]^ Another article reported SilMA/HAMA/Cu‐EGCG, a multifunctional hydrogel dressing with high strength, anti‐swelling, antimicrobial, and antioxidant capabilities. Cu^2+^ and EGCG served as functional substances that acted on the bacterial outer membrane, leading to its rupture. It was shown that the release of Cu^2+^ was effectively prolonged by the dual locking effect of the hydrogel and the metal polyphenol network. Thus, this hydrogel significantly promoted the healing rate of infected wounds and reduced scar formation through the sustained release of Cu^2^⁺ and EGCG, and displayed good biocompatibility and clinical translational prospects (**Figure** **15**a). The metal polyphenol capsule (Cu‐EGCG) played a key role in this and achieved efficient treatment of infected wounds through multiple mechanisms, including antimicrobial, antioxidant, and pro‐angiogenic.^[^ ^184^ ^]^ In addition, the multiple phenolic groups present in natural polyphenols undergo various interactions with amino acids, metal ions, and other units, thereby greatly enhancing the adhesion strength and stability of the biological gel in the internal microenvironment.^[^ ^185^ ^]^ The previous study had developed a novel bio‐glue based on visible‐light responsive polyphenol–acrylamide–ruthenium cross‐linking reaction, which had an adhesion strength of up to 500 kPa, and was much higher than that of the conventional acrylamide‐based glues (250 kPa), through the multiple interactions between polyphenols (e.g., TA, EGCG) and the tissues (hydrogen‐bonding, covalent‐bonding, and cation–π interactions).^[^ ^186^ ^]^

### 5.3 Drug Delivery Systems

#### 5.3.1 Antioxidant and Anti‐Inflammatory Therapy

In the past few years, polyphenols have gained particular prominence in disease treatment and biomaterial development due to their antioxidant and anti‐inflammatory properties. Phenolic hydroxyl groups in polyphenol molecules effectively neutralize ROS and reactive nitrogen species through hydrogen supply and electron transfer mechanisms, thus reducing cellular damage induced by oxidative stress. This property not only scavenges free radicals directly but also enhances endogenous antioxidant capacity by activating intracellular signaling pathways (e.g., the Nrf2 pathway) and inducing regulated antioxidant enzyme activities such as SOD, glutathione peroxidase (GPx), and CAT.^[^ ^187^ ^]^ Additionally, it could regulate the activity of transcription factors (such as Nrf2 and NF‐κB) by activating signaling pathways like PI3K and MAPK, thereby influencing antioxidant gene expression and inflammatory responses.^[^ ^188^ ^]^ By inhibiting p38 MAPK phosphorylation and blocking the expression of downstream pro‐inflammatory factors (such as IL‐6, TNF‐α, iNOS, and COX‐2), it shows anti‐inflammatory effects. This provides an efficient and low‐toxicity treatment strategy for chronic inflammatory diseases such as osteoarthritis.^[^ ^189^ ^]^ For instance, research has reported a one‐step assembly of polyphenols, iron chelators, and alpha‐lipoic acid to produce an injectable hydrogel. In vitro and in vivo studies demonstrated that the prepared hydrogel not only exhibited excellent antioxidant and anti‐inflammatory properties but also possessed distinctive features such as iron chelation, anti‐adhesion, and wear resistance, offering promising prospects for clinical application as a therapeutic agent.^[^ ^190^ ^]^

Additionally, studies have reported that polyphenols, as part of a drug delivery carrier, could be used to alleviate symptoms of inflammatory bowel disease. Researchers developed a copper–lignin nanocomposite (CuL NCs), and experimental results showed that CuL NCs regulated the inflammatory microenvironment by scavenging ROS, modulating the Nrf2/HO‐1 and NF‐κB pathways, and regulating the inflammatory microenvironment (Figure 15b). In mouse models of ulcerative colitis and Crohn`s disease, CuL NCs demonstrated significant preventive and therapeutic effects by reducing inflammation, oxidative stress, and restoring intestinal homeostasis. Thus, CuL NCs exhibited multifunctional regulation of oxidative stress, inflammation, intestinal barrier function, and the intestinal microbiota.^[^ ^191^ ^]^

#### 5.3.2 Antitumor Effects

Polyphenols often play a role in plants in preventing invasion from pathogens or pests. As a result, polyphenols have been extensively studied for their antitumor activity. It had been demonstrated that systems based on the assembly of Fe^3+^ and TA provided drug‐carrying nanoparticles, which had small and uniform sizes and showed beneficial potential in enhancing colloidal stability and preventing premature drug leakage. In addition, the metal phenolic coating was found to be highly cell biocompatible as a delivery vehicle for controlled drug delivery, whereas the final prepared drug nanoparticles possessed potent antitumor activity by inducing higher apoptosis and inhibiting tumor metastasis, which was superior to that of the bare drug formulation.^[^ ^192^ ^]^ Moreover, polyphenols are used to achieve efficient inhibition of recurrence and metastasis of tumors after surgery through the construction of magnetically responsive hydrogels integrating multiple mechanisms such as magnetic thermotherapy, focal death induction, immune activation, and metal ion modulation.^[^ ^193^ ^]^ For example, it had been reported that the phenolic hydroxyl group of TA combined with ions such as Fe^3^⁺ and Zn^2^⁺ might inhibit tumor cells by regulating the intracellular metal ion concentration, affecting the redox balance, and inducing oxidative stress. Polyphenols helped immobilize magnetic nanoparticles to improve the magneto–thermal conversion efficiency, and local high temperature induced tumor cell focal death. Finally, the systemic immune response triggered by local treatment inhibited distant tumor growth and lung metastasis (Figure 15c).^[^ ^193^ ^]^ Moreover, the multifunctional nanomedicine platform composed of nanomedicines and polyphenols self‐assembled and employed a three‐pronged strategy combining photothermal therapy, immunotherapy, and ferroptosis to efficiently induce tumor cell necrosis and apoptosis, then reversed tumor immune suppression.^[^ ^105^, ^194^ ^]^

Thus, it was seen that the polyphenol‐based smart drug delivery system had achieved precise release to tumor targets, significantly improved drug bioavailability, and reduced toxicity to normal tissues. These combined effects not only provide a solid theoretical basis for polyphenol‐based antitumor strategies but also open up new avenues for clinical translation in precision tumor therapy.^[^ ^195^ ^]^

#### 5.3.3 Suppresses the Rise in Blood Glucose

In addition to the aforementioned drug delivery applications of polyphenols in wound healing, antioxidant, and antitumor applications, polyphenols have attracted much attention in recent years for their multiple roles in regulating energy metabolism and glucose metabolism signaling, and their hypoglycemic effects have been demonstrated in a variety of in vitro and in vivo models.^[^ ^196^ ^]^ Polyphenols (e.g., anthocyanins, phenolic acids) improve postprandial hyperglycemia by regulating glucose transporter protein (GLUT4), the AMPK pathway, and glucagon (GLP‐1).^[^ ^197^ ^]^ A study had reported that wheat starch‐Lonicera caerulea berry polyphenol complex had a postprandial glucose‐lowering effect in T2DM model mice. The results indicated that exposure to wheat starch‐Lonicera caerulea berry polyphenol complex activated the GLP‐1R/PI3K/AKT signaling pathway in the liver tissues of T2DM mice, thus reducing insulin resistance.^[^ ^137^ ^]^ In addition, polyphenols delayed the hydrolysis of intestinal carbohydrates to monosaccharides by targeting α‐glucosidase and α‐amylase, then reduced postprandial blood glucose peaks. For example, walnut septum polyphenol inhibited α‐glucosidase with the IC50 value of 36.2 µg mL^−1^, and the synergistic inhibition with pancreatic lipase improved lipid metabolism disorders in obese diabetes.^[^ ^196^ ^]^

### 5.4 Other Biomedical Aspects

#### 5.4.1 Diagnostics and Imaging

**Figure 16:**
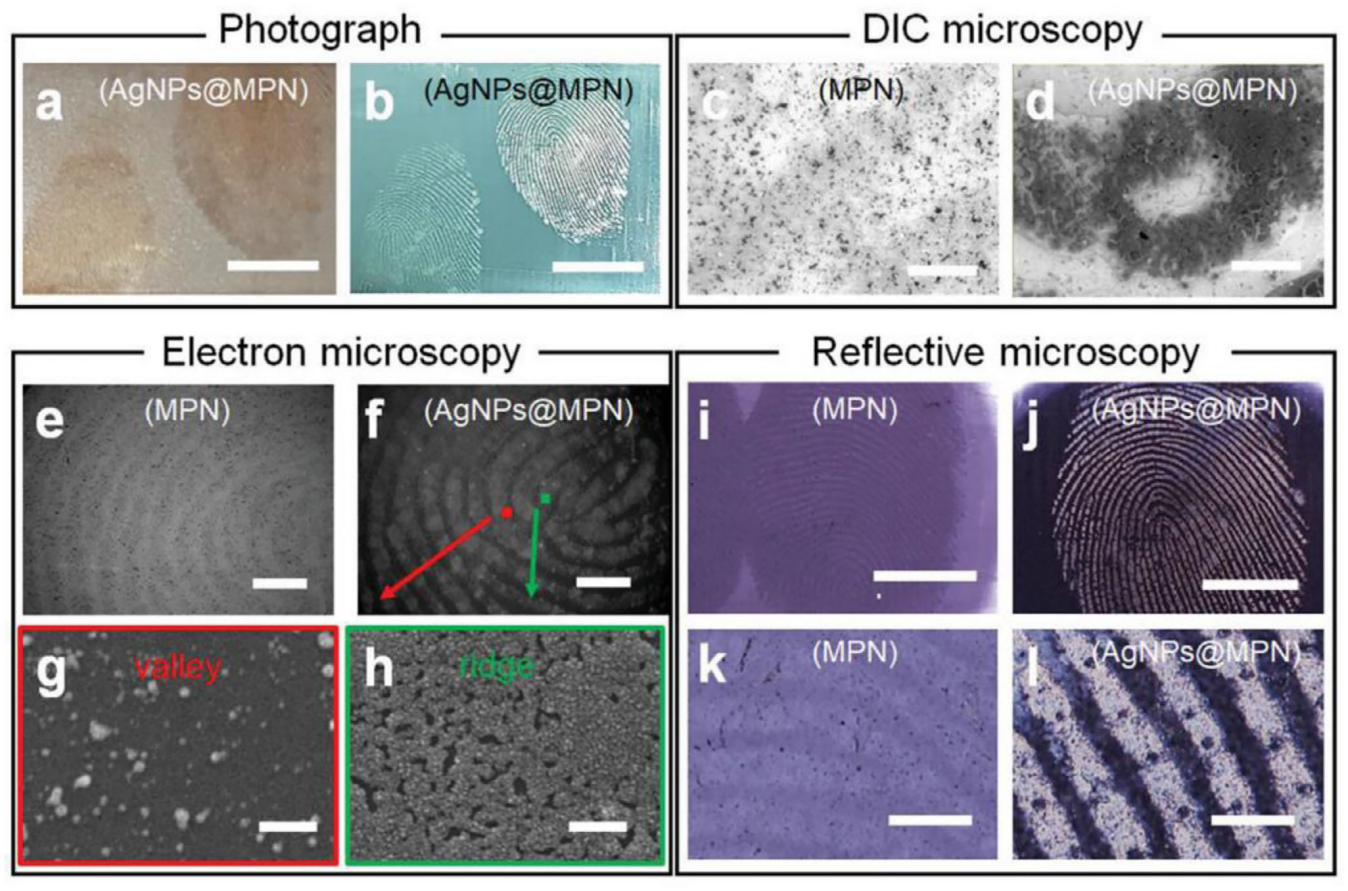
a) Characterization of fingerprint patterns (c,e,i,k) after deposition of MPN and subsequent formation of AgNP (a,b,d,f,g,h,j,l) highlighting natural biomolecule patterns (i.e., a,b) cell phone digital photography (without a) and with b) flash); c,d) DIC microscopy. e–h) Scanning electron microscopy (SEM). i–l) Reflection microscopy. Scale bars a,b) 1 cm, c,d) 200 µm, e,f) 1 mm, g,h) 1 µm, i,j) 0.5 cm, k,l) 400 µm.^[^ ^198^ ^]^ Copyright 2019, Wiley.

**Figure 17:**
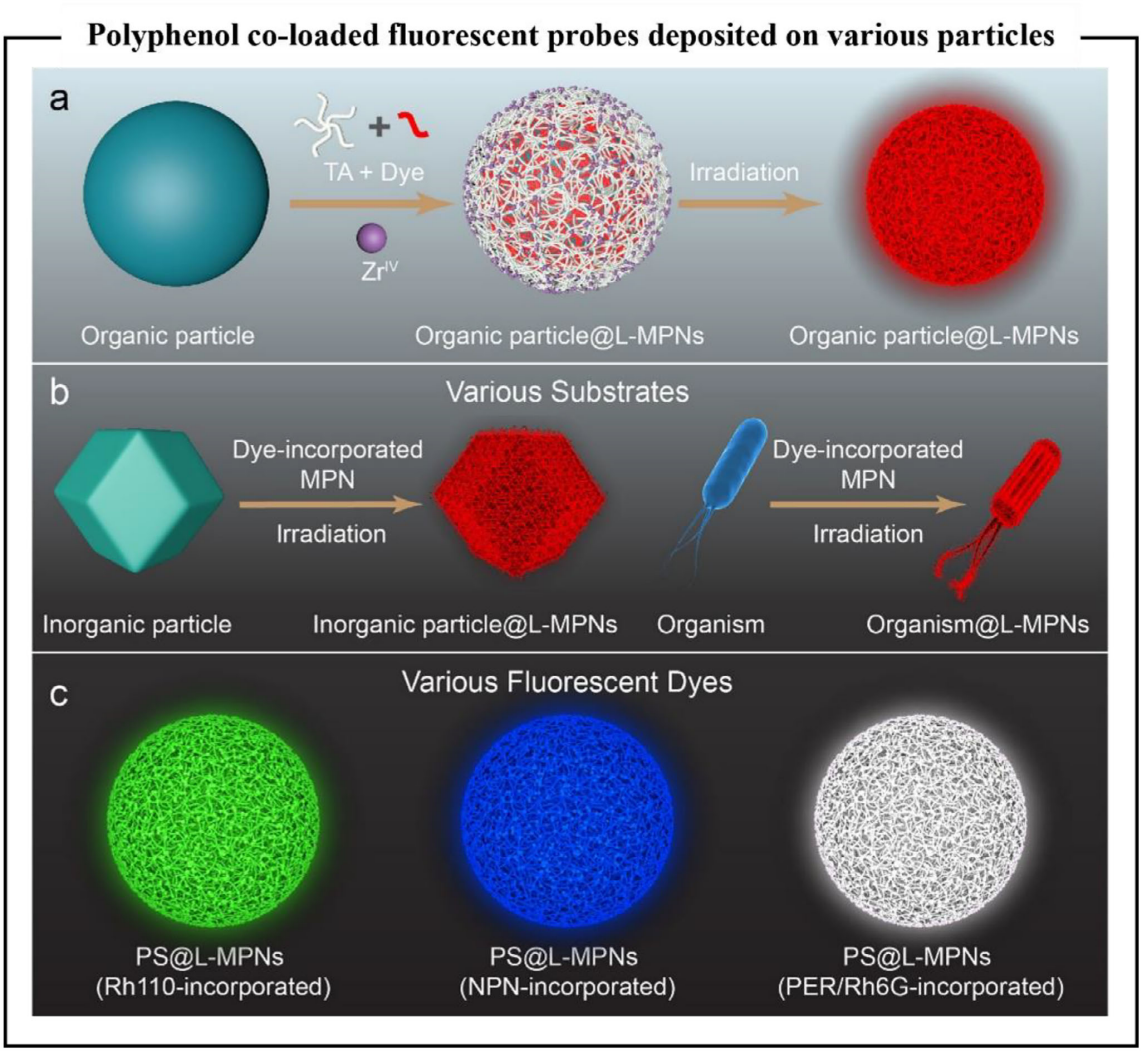
a) Schematic representation of L‐MPNs deposited on particles (particle@L‐MPNs) and subsequently fluorescing. b) L‐MPNs assembled on a variety of particles, including inorganic and biological entities. c) Schematic of the incorporation of different fluorescent dyes. Rh110 = rhodamine 110 chloride, NPN = N‐phenyl‐1‐naphthylamine, PER = perylene, Rh6G = rhodamine 6G.^[^ ^199^ ^]^ Copyright 2021, Wiley.

Recent advances in polyphenols in medicine have motivated unprecedented opportunities for precision therapy and bioassays. Advanced imaging modalities, including PET, MRI, and near‐infrared fluorescence, could be realized through metal ion chelation or fluorescent dye encapsulation, thus enhancing spatial and temporal tracking of therapeutic outcomes.^[^ ^8^ ^]^ MPNs have emerged as versatile scaffolds for decoding the phenomenon of “biomolecular corona.” It had been shown that by reverse‐engineering the interactions between NPs and biomolecules on a planar substrate, MPNs can selectively pattern proteins, lipids, nucleic acids, and polysaccharides, enabling ultrasensitive detection of latent fingerprints through AgNP‐mediated reflection contrast (**Figure** **16**).^[^ ^198^ ^]^ Meanwhile, a study had pioneered a supramolecular fluorescent labeling platform that utilizes porous MPNs as dynamic hosts for commercial dyes. Through π‐interaction‐driven assembly, researchers engineered ultrathin (10 nm), pH‐stabilized coatings on different particles (inorganic, organic, and biological) with customizable emission profiles, including red, blue, white, and multicolor patterns (**Figure** **17**).^[^ ^199^ ^]^ This strategy helped to track cellular uptake and endosomal transport in HeLa cells in real time, while also maintaining an order of magnitude higher photostability than free dyes. Taken together, these studies illustrated the transformative potential of polyphenols for next‐generation bio‐detection, imaging, forensics, and diagnostics, and as a link between synthetic material design and complex biological systems.

#### 5.4.2 Neurological Treatment

Ferroptosis is an iron‐dependent cell death pathway. Ferroptosis in biological organisms is closely associated with neuronal damage. Excess iron ions catalyze the Fenton reaction to generate free radicals, leading to lipid peroxidation and triggering iron‐dependent programmed cell death, thereby exacerbating neuronal damage and cognitive decline,^[^ ^200^ ^]^ such as in Alzheimer's disease, Parkinson's disease, and irreversible spinal cord injury.^[^ ^201^ ^]^ Due to the unique structure and biological activity of polyphenols, recent studies have explored their use as adjunctive therapies for neurological disorders. The research had developed a quercetin‐based triphenylphosphine‐modified nanomedicine, which targeted the brain mitochondria of Alzheimer's disease mice by intranasal administration and utilizes the self‐assembly properties of plant polyphenols to form metal–phenol nanocomplexes. The drug worked through a dual mechanism: on the one hand, it chelated excess iron ions and scavenges free radicals to inhibit lipid peroxidation; on the other hand, it activated the Nrf2 signaling pathway to restore the balance of iron metabolism (promoting the expression of the iron storage protein FTH1 and the iron export protein FPN1) and the activity of antioxidant enzymes (GPX4, SOD), thus alleviated the neuronal damage triggered by iron death. In vivo studies have confirmed that the quercetin‐based triphenylphosphine‐modified nanomedicine significantly ameliorated cognitive deficits and reduced cerebral iron deposition, Aβ plaques, and oxidative stress markers in Alzheimer's disease mice, providing a new multi‐targeted synergistic therapeutic strategy for iron death‐driven neuronal degenerative diseases.^[^ ^202^ ^]^ Additionally, researchers had reported that polyphenol‐based hydrogels chelated excess iron ions and regulated iron transport to activate intrinsic neurogenesis after spinal cord injury, thereby stimulating the regenerative potential of new neurons. The primary regulatory mechanisms involve: 1) upregulating the xCT/GPX4 axis to restore neuronal iron homeostasis, inhibit GSH consumption, and regulate iron transport and metabolism; and 2) combining metal chelation and antioxidant properties to promote scalable differentiation of neural stem cells into neurons and the formation of functional synapses.^[^ ^201^ ^]^ This study demonstrated that polyphenol‐based hydrogels served as a protective barrier for neuronal iron homeostasis, promoting neurogenesis and functional recovery following iron‐accumulating neurological disorders.

## 6 Conclusions and Perspectives

Over the past few years, the use of polyphenols in combination with various substances has been increasingly studied in biomedical fields such as bioreactors, biomaterials, drug delivery, and medical imaging. However, there is a lack of systematic and comprehensive research on the mechanisms and applications of polyphenol assembly with various substances. Therefore, this paper focuses on how polyphenols interact with substances such as metals, proteins, polysaccharides, nucleic acids, and other polyphenols through non‐covalent and covalent bonds to modify or synergize with each other, thereby enhancing their functional properties. Polyphenol complexes exist in various forms, including nanoparticles, coatings, capsules, and hydrogels, and different forms of polyphenol complexes perform corresponding functions according to the specific application scenarios required.

Despite the remarkable achievements in the research of polyphenolic materials in the biomedical field, significant challenges and difficulties still remain. 1) Potential toxicity: polyphenolic structures are complex, and systematic studies on their safety for human use are lacking. 2) Unstable biological activity: the phenolic hydroxyl groups in polyphenols are prone to oxidation and loss of biological activity when exposed to air, light, or physiological environments, leading to the loss of their biological activity. 3) There is currently no method for detecting the entire process of polyphenol self‐assembly product formation. Polyphenol assembly with other substances involves various covalent and non‐covalent interactions, but no strategies to monitor these reaction processes, limiting the summarization and presentation of polyphenol self‐assembly principles. 4) There are many functional materials developed based on polyphenols, but few drugs have actually been clinically converted.

Overall, polyphenols not only provide powerful antioxidant, anti‐inflammatory, and immunomodulatory functions to traditional materials, but also improve the stability, adhesion, and environmental responsiveness of the materials by integrating them with novel materials, which significantly enhances their drug delivery efficiency, wound repair, antibacterial, and anti‐inflammatory properties, and the potential for precision treatment of diseases. Thus, polyphenols are extensively applied in numerous domains.^[^ ^9^, ^203^, ^204^ ^]^ However, there are still spaces for both the fundamental and the applied research on polyphenols. We highlight here some possible areas of research to help further advance the field. 1) Expanding the mixing of polyphenols with different kinds of biomaterials (one or more mixes) may lead to the discovery of new structural features and functional materials. 2) Extended research on the application of polyphenols in different fields, currently known to be in the fields of wastewater treatment, biomedicine, tissue materials, and food. Future applications of polyphenols in other areas (e.g., aerospace, textiles, building materials, etc.) could be explored. 3) To date, extensive research has been reported on typical polyphenolic substances such as tannins, EGCG, and dopamine. Future studies should expand to include other polyphenolic substances to discover more functional polyphenols beneficial to humans and advance the clinical translation of polyphenol‐based drugs or biomaterials. Polyphenol materials exhibit excellent biocompatibility, biodegradability, and environmental friendliness. Efforts should be made to promote the clinical application of polyphenol drugs and biomaterials with practical development as the goal. 4) In the field of biomaterials research, it is essential to clarify the effects of non‐covalent and covalent interactions on the functional properties (structural stability, viscosity, elasticity, biocompatibility, etc.) of polyphenols after their combination with other substances, in order to better understand the structural changes in biomaterials achieved through chemical means. Currently, researches have shown that AI could be used to identify the types of phenolic acids, with machine learning algorithms analyzing them, achieving an accuracy rate of up to 99.0%.^[^ ^205^ ^]^ In the future, AI algorithms could be utilized for the design and optimization of polyphenol‐based materials, with machine learning used to predict the biological activity and interaction mechanisms between polyphenols and other materials to simulate the formation process of polyphenol self‐assembly. 5) Polyphenols are being developed in various ways, such as cell modeling, 3D bioprinting, hydrogel scaffolds, and bioactive coatings. In the context of artificial intelligence (AI), biomaterials with smart responsiveness and smart functions will attract significant attention. For example, polyphenols combined with conductive polymers and carbon nanomaterials play a role in bioelectronics, wearable medical devices, and electrical stimulation therapy. In the future, polyphenols could be further explored in fields such as algorithmic simulation of cellular structure and composition, artificial intelligence biosensors, and smart functional tissue materials. Consequently, in the context of artificial intelligence, methods that integrate multidisciplinary knowledge and scientific exploration have the potential for in‐depth exploration and further discovery.

## Conflict of Interest

The authors declare no conflict of interest.

## References

[1] Z. Lin , H. Liu , J. J. Richardson , W. Xu , J. Chen , J. Zhou , F. Caruso , Chem. Soc. Rev. 2024, 53, 10800. 39364569 10.1039/d3cs00273j

[2] M. A. Rahim , S. L. Kristufek , S. J. Pan , J. J. Richardson , F. Caruso , Angew. Chem. Int. Ed. 2019, 58, 1904. 10.1002/anie.201807804 30221440

[3] a) Y. X. Guo , Q. Sun , F. G. Wu , Y. L. Dai , X. Y. Chen , Adv. Mater. 2021, 33, 2007356; 10.1002/adma.202007356 33876449

[4] D. Wu , J. Zhou , M. N. Creyer , W. Yim , Z. Chen , P. B. Messersmith , J. V. Jokerst , Chem. Soc. Rev. 2021, 50, 4432. 33595004 10.1039/d0cs00908c PMC8106539

[5] X. G. Dang , Y. T. Fu , X. C. Wang , Adv. Funct. Mater. 2024, 34, 2405745.

[6] W. Liu , R. Zhang , G. Duan , L. Zhang , Y. Li , L. Yang , Adv. Fiber Mater. 2024, 6, 952.

[7] T. Wang , J. Zhao , Z. Yang , L. Xiong , L. Li , Z. Gu , Y. Li , Green Chem. 2022, 24, 3605.

[8] H. Cao , L. Yang , R. Tian , H. Wu , Z. Gu , Y. Li , Chem. Soc. Rev. 2022, 51, 4175. 35535743 10.1039/d1cs01165k

[9] Y. Liu , Y. Shi , M. Zhang , F. Han , W. Liao , X. Duan , Eur. J. Med. Chem. 2024, 266, 116141. 38237341 10.1016/j.ejmech.2024.116141

[10] X. Zhang , Z. Li , P. Yang , G. Duan , X. Liu , Z. Gu , Y. Li , Mater. Horiz. 2021, 8, 145. 34821294 10.1039/d0mh01317j

[11] Q. Wang , J. Chen , J. Ling , H. Zhao , X. K. Ouyang , N. Wang , Mater. Today Chem. 2024, 35, 101892.

[12] Y. Zou , X. Wang , Y. Li , Y. Cheng , Mater. Today 2024, 79, 112.

[13] S. Oliver , O. Vittorio , G. Cirillo , C. Boyer , Polym. Chem. 2016, 7, 1529.

[14] D. Çevik , Y. Kan , H. Kırmızıbekmez , Phytomedicine 2019, 58, 152872. 30826527 10.1016/j.phymed.2019.152872

[15] A. Raal , A. Vahtra , O. Koshovyi , T. Ilina , A. Kovalyova , T. Püssa , Molecules 2024, 29, 5016. 39519657 10.3390/molecules29215016 PMC11547628

[16] A. Borah , M. Gogoi , R. Goswami , S. Hazarika , Ind. Crops Prod. 2024, 209, 117986.

[17] A. Mir‐Cerdà , M. Granados , J. Saurina , S. Sentellas , Food Chem. 2024, 456, 140042. 38876070 10.1016/j.foodchem.2024.140042

[18] L. Wen , T. Zhou , Y. Jiang , L. Gong , B. Yang , Phytomedicine 2021, 90, 153641. 34281775 10.1016/j.phymed.2021.153641

[19] J. Vaya , S. Mahmood , BioFactors 2006, 28, 169. 17473377 10.1002/biof.5520280303

[20] a) C. Sehaki , N. Jullian , F. Ayati , F. Fernane , E. Gontier , plants 2023, 12, 279; 36678991 10.3390/plants12020279 PMC9866577

[21] a) S. Silva , E. M. Costa , M. Veiga , R. M. Morais , C. Calhau , M. Pintado , Crit. Rev. Food Sci. 2020, 60, 181; 10.1080/10408398.2018.1518895 30373383

[22] K. Simkova , R. Veberic , M. C. Grohar , M. Pelacci , T. Smrke , T. Ivancic , A. Medic , N. C. Weber , J. Jakopic , Plants 2024, 13, 1419. 38794489 10.3390/plants13101419 PMC11125040

[23] K. S. Coutinho‐Wolino , M. F. S. Melo , J. C. Mota , D. Mafra , J. T. Guimarães , M. B. Stockler‐Pinto , Nutr. Rev. 2023, 82, 248. 10.1093/nutrit/nuad048 37164634

[24] Y. Liu , N. Yan , Q. Chen , L. Dong , Y. Li , P. Weng , Z. Wu , D. Pan , L. Liu , M. A. Farag , L. Wang , L. Liu , Crit. Rev. Food Sci. Nutr. 2024, 64, 11493. 37552798 10.1080/10408398.2023.2239350

[25] H. A. R. Suleria , C. J. Barrow , F. R. Dunshea , Foods 2020, 9, 1206. 32882848 10.3390/foods9091206 PMC7556026

[26] H. Dong , H. Ye , W. Bai , X. Zeng , Q. Wu , Compr. Rev. Food Sci. Food Saf. 2024, 23, 70032. 10.1111/1541-4337.70032 39523696

[27] A. N. Panche , A. D. Diwan , S. R. Chandra , J. Nutr. Sci. 2016, 5, 47. 10.1017/jns.2016.41 PMC5465813 28620474

[28] Z.‐Q. Liu , Eur. J. Med. Chem. 2022, 243, 114671. 36088759 10.1016/j.ejmech.2022.114671

[29] X. Wang , Y. Cao , S. Chen , J. Lin , J. Bian , D. Huang , J. Agric. Food Chem. 2021, 69, 7285. 34160206 10.1021/acs.jafc.1c02015

[30] D. Barreca , D. Trombetta , A. Smeriglio , G. Mandalari , O. Romeo , M. R. Felice , G. Gattuso , S. M. Nabavi , Trends Food Sci. Technol. 2021, 117, 194.

[31] Y. Xu , J. Hu , J. Hu , Y. Cheng , X. Chen , Z. Gu , Y. Li , Prog. Polym. Sci. 2023, 146, 101740.

[32] R. Amarowicz , R. B. Pegg , Adv. Food Nutr. Res. 2024, 110, 327. 38906590 10.1016/bs.afnr.2024.03.001

[33] M. Molnar , M. J. Kovač , V. Pavić , Molecules 2024, 29, 2615. 38893491 10.3390/molecules29112615 PMC11173854

[34] Z. Dehghanian , K. Habibi , M. Dehghanian , S. Aliyar , B. A. Lajayer , T. Astatkie , T. Minkina , C. Keswani , Helyon 2022, 8, 09094. 10.1016/j.heliyon.2022.e09094 PMC8927939 35309390

[35] F. Seidi , Y. Liu , Y. Huang , H. Xiao , D. Crespy , Chem. Soc. Rev. 2025, 54, 3140. 39976198 10.1039/d4cs00440j

[36] P. L. de Hoyos‐Martínez , J. Merle , J. Labidi , F. Charrier ‐ El Bouhtoury , J. Clean. Prod. 2019, 206, 1138.

[37] S. Molino , M. Pilar Francino , J. Ángel Rufián Henares , Food Res. Int. 2023, 173, 113329. 37803691 10.1016/j.foodres.2023.113329

[38] S. Shrestha , W. Zhang , S. D. Smid , Food Biosci. 2021, 39, 100832.

[39] W. Zhang , Q. Zhang , J. Li , X. Ren , Y. Zhang , Q. Niu , J. Agric. Food Chem. 2024, 72, 2634. 38267223 10.1021/acs.jafc.3c08886

[40] N. B. Bottari , L. Q. S. Lopes , K. Pizzuti , C. Filippi dos Santos Alves , M. S. Corrêa , L. P. Bolzan , A. Zago , R. de Almeida Vaucher , A. A. Boligon , J. L. Giongo , M. D. Baldissera , R. C. V. Santos , Microb. Pathogen. 2017, 104, 190. 28126664 10.1016/j.micpath.2017.01.037

[41] a) R. B. Teponno , S. Kusari , M. Spiteller , Nat. Prod. Rep. 2016, 33, 1044; 27157413 10.1039/c6np00021e

[42] L. P. Meagher , G. R. Beecher , V. P. Flanagan , B. W. Li , J. Agric. Food Chem. 1999, 47, 3173. 10552626 10.1021/jf981359y

[43] Y. Hu , Y. Li , L. Sampson , M. Wang , J. E. Manson , E. Rimm , Q. Sun , J. Am. Coll. Cardiol. 2021, 78, 666. 34384548 10.1016/j.jacc.2021.05.049 PMC8432598

[44] Y. Yang , J. Guo , H. Cao , X. Tian , H. Shen , J. Niu , H. Yang , Q. Shi , Y. Xu , Biomaterials 2025, 321, 123326. 40239592 10.1016/j.biomaterials.2025.123326

[45] F. Zamani‐Garmsiri , S. Emamgholipour , S. R. Fard , G. Ghasempour , R. J. Ahvazi , R. Meshkani , Phytother. Res. 2022, 36, 415. 34825416 10.1002/ptr.7329

[46] Y. Li , R. Fu , Z. Duan , C. Zhu , D. Fan , Bioact. Mater. 2022, 9, 461. 34820583 10.1016/j.bioactmat.2021.07.023 PMC8586748

[47] Y. Qian , C. Wang , R. Xu , J. Wang , Q. Chen , Z. Zhu , Q. Hu , Q. Shen , J. Shen , J. Nanobiotechnol. 2025, 23, 135. 10.1186/s12951-025-03220-5 PMC11847370 39987136

[48] N. K. Campbell , H. K. Fitzgerald , J. M. Fletcher , A. Dunne , 2019, 10.

[49] Y. Yang , S. Chen , G. Shi , S. Huang , N. Cui , L. Tan , X. Yang , Phytomedicine 2025, 140, 156572. 40023112 10.1016/j.phymed.2025.156572

[50] D. Nasiry , A. R. Khalatbary , Nutr. Neurosci. 2025, 28, 952. 39825479 10.1080/1028415X.2024.2448924

[51] J. Wang , J. Qu , S. Liu , Q. Xu , X. Li , Y. Zhu , X. Liu , J. Yi , Z. Yuan , P. Huang , Y. Yin , L. Wen , J. Wu , J. Agric. Food Chem. 2023, 71, 12574. 37525894 10.1021/acs.jafc.3c02934

[52] C. F. Silva , P. L. Rosalen , J. C. Soares , A. P. Massarioli , L. H. Campestrini , R. A. Semarini , S. M. Alencar , J. Apicult. Res. 2020, 59, 136.

[53] a) L. G. Naso , E. G. Ferrer , P. A. M. Williams , Coord. Chem. Rev. 2023, 492, 215271;

[54] J. H. Zhang , Y. Fu , P. Yang , X. H. Liu , Y. W. Li , Z. P. Gu , Adv. Mater. Interfaces 2020, 7, 2000632.

[55] S. B. Nimse , D. Pal , RSC Adv. 2015, 5, 27986.

[56] C. Carrasco‐Pozo , K. N. Tan , M. Reyes‐Farias , N. De La Jara , S. T. Ngo , D. F. Garcia‐Diaz , P. Llanos , M. J. Cires , K. Borges , Redox Biol. 2016, 9, 229. 27591402 10.1016/j.redox.2016.08.007 PMC5011185

[57] K. A. Dias , O. L. Alves , P. S. M. Santana , A. L. C. Sette , V. L. C. O. d'Souza , G. R. Vilela , C. M. Della Lucia , Crit. Rev. Food Sci. Nutr. 2025, 10.1080/10408398.2025.24728821.

[58] Y. Du , H. Ding , K. Vanarsa , S. Soomro , S. Baig , J. Hicks , C. Mohan , Nutrients 2019, 11, 1743. 31362373 10.3390/nu11081743 PMC6724056

[59] H. Choi , J. S. Park , K. M. Kim , M. Kim , K. W. Ko , C. G. Hyun , J. W. Ahn , J. H. Seo , S. Y. Kim , J. Ind. Eng. Chem. 2018, 63, 255.

[60] M. Mokhtar , G. Ginestra , F. Youcefi , A. Filocamo , C. Bisignano , A. Riazi , Curr. Microbiol. 2017, 74, 1253. 28721659 10.1007/s00284-017-1310-2

[61] a) W. J. Xu , Z. X. Lin , C. Cortez‐Jugo , G. G. Qiao , F. Caruso , Angew. Chem. Int. Ed. 2025, 64, 202423654; 10.1002/anie.202423654 39905990

[62] S. Jahanizadeh , F. Yazdian , A. Marjani , M. Omidi , H. Rashedi , Int. J. Biol. Macromol. 2017, 105, 757. 28746888 10.1016/j.ijbiomac.2017.07.101

[63] O. M. Ahmed , A. A. Ahmed , H. Fahim , M. Y. Zaky , Drug and Chemical Toxicology 2022, 45, 262. 31665932 10.1080/01480545.2019.1683187

[64] S. T. Han , F. Y. Lin , Y. C. Qi , C. Liu , L. X. Zhou , Y. Q. Xia , K. Chen , J. Xing , Z. L. Liu , W. M. Yu , Y. L. Zhang , X. J. Zhou , T. Rao , F. Cheng , Oxid. Med. Cell. 2022, 2022, 3846217. 10.1155/2022/3846217 PMC9153929 35656025

[65] M. Huo , A. Xia , W. Cheng , M. Zhou , J. Wang , T. Shi , C. Cai , W. Jin , M. Zhou , Y. Liao , Z. Liao , Molecules 2022, 27, 2293. 35408691 10.3390/molecules27072293 PMC9000526

[66] Z. Zhang , C. Qiu , X. Li , D. J. McClements , A. Jiao , J. Wang , Z. Jin , Trends Food Sci. Technol. 2021, 116, 492.

[67] Y. Zhu , Z. Q. Fang , J. Bai , L. H. Wang , J. Q. Chen , Z. H. Zhang , Q. Wang , W. W. Sheng , X. Y. Pan , Z. Y. Gao , D. Q. Xu , P. K. Wu , B. C. Sun , Adv. Sci. 2025, 12, 2411939. 10.1002/advs.202411939 PMC12061243 40067175

[68] H. Liang , B. Zhou , D. Wu , J. Li , B. Li , Adv. Colloid Interface Sci. 2019, 272, 102019. 31445352 10.1016/j.cis.2019.102019

[69] S. Y. Park , S. Kim , S. Y. Shin , W. K. Cho , K. M. Huh , Chem. Eng. J. 2024, 493, 152286.

[70] Z. Fang , X. Guan , J. He , Nano Energy 2025, 133, 110498.

[71] S. Tan , J. Han , X. Yuan , Z. Song , L. Gao , J. Gao , L. Wang , Mater. Des. 2023, 227, 111701.

[72] X. H. Guo , W. K. Luo , L. Y. Wu , L. L. Zhang , Y. X. Chen , T. Li , H. G. Li , W. Zhang , Y. W. Liu , J. Zheng , Y. Wang , Adv. Sci. 2024, 11, 2403388. 10.1002/advs.202403388 PMC11425287 39033533

[73] L. Liu , H. Shi , H. Yu , R. Zhou , J. Yin , S. Luan , Biomater. Sci. 2019, 7, 5035. 31535105 10.1039/c9bm01223k

[74] W. Shu , W. Shi , H. Xie , S. Wang , Q. Zhang , K. Ouyang , F. Xiao , Q. Zhao , Food Chem. 2025, 479, 143732. 40073562 10.1016/j.foodchem.2025.143732

[75] Q. Zhao , X. Yu , C. Zhou , A. E. A. Yagoub , H. Ma , LWT 2020, 124, 109192.

[76] F. J. M. Hoeben , P. Jonkheijm , E. W. Meijer , A. P. H. J. Schenning , Chem. Rev. 2005, 105, 1491. 15826018 10.1021/cr030070z

[77] H. Yan , L. Li , Z. Wang , Y. Wang , M. Guo , X. Shi , J.‐M. Yeh , P. Zhang , ACS Biomater. Sci. Eng. 2020, 6, 634. 33463207 10.1021/acsbiomaterials.9b01601

[78] P. Chen , S. B. He , Z. K. Zou , T. Y. Wang , J. F. Hu , J. R. Tao , L. Yang , Y. W. Li , Adv. Funct. Mater. 2025, 2506308.

[79] P. Y. Zhang , H. M. Geng , Z. L. Gao , J. C. Hao , J. W. Cui , Adv. Mater. Interfaces 2022, 9, 2200779.

[80] M. Li , H. Wang , J. Hu , J. Hu , S. Zhang , Z. Yang , Y. Li , Y. Cheng , Chem. Mater 2019, 31, 7678.

[81] a) J. Zhou , Z. Lin , Y. Ju , M. A. Rahim , J. J. Richardson , F. Caruso , Acc. Chem. Res. 2020, 53, 1269; 32567830 10.1021/acs.accounts.0c00150

[82] Z. Jia , Y. Zeng , P. Tang , D. Gan , W. Xing , Y. Hou , K. Wang , C. Xie , X. Lu , Chem. Mater. 2019, 31, 5625.

[83] H. Luo , J. Xie , X. Su , P. Wang , H. Chen , X. Kuang , J. Liu , Sci. China Mater. 2024, 67, 3833.

[84] Z. Zhang , L. S. Xie , Y. Ju , Y. L. Dai , Small 2021, 17, 2100314. 10.1002/smll.202100314 34018690

[85] L. He , D. E. Fullenkamp , J. G. Rivera , P. B. Messersmith , Chem. Commun. 2011, 47, 7497. 10.1039/c1cc11928a PMC4526106 21629956

[86] B. Liu , J. Li , Z. Zhang , J. D. Roland , B. P. Lee , Chem. Eng. J. 2022, 441, 135808. 35444488 10.1016/j.cej.2022.135808 PMC9015688

[87] D. Li , M. Li , L. Wang , J. Zhang , X. Wang , J. Nie , G. Ma , J. Mater. Chem. B 2024, 12, 2571. 38363109 10.1039/d3tb02685j

[88] W. Kim , Y. Wang , C. Selomulya , Trends Food Sci. Technol. 2024, 147, 104469.

[89] B. D. Mather , K. Viswanathan , K. M. Miller , T. E. Long , Prog. Polym. Sci. 2006, 31, 487.

[90] Z. Chen , C. Wang , J. Chen , X. Li , J. Am. Chem. Soc. 2013, 135, 4179. 23470166 10.1021/ja311374b

[91] H. Qiu , S. Wang , R. Huang , X. Liu , L. Li , Z. Liu , A. Wang , S. Ji , H. Liang , B.‐P. Jiang , X.‐C. Shen , Biomater. Sci. 2024, 12, 3175. 38742916 10.1039/d4bm00490f

[92] C. Sun , X. Zeng , S. Zheng , Y. Wang , Z. Li , H. Zhang , L. Nie , Y. Zhang , Y. Zhao , X. Yang , Chem. Eng. J. 2022, 427, 130843.

[93] J. H. Cho , J. S. Lee , J. Shin , E. J. Jeon , S. An , Y. S. Choi , S. W. Cho , Adv. Funct. Mater. 2018, 28, 1705244.

[94] S. Hong , K. Yang , B. Kang , C. Lee , I. T. Song , E. Byun , K. I. Park , S. W. Cho , H. Lee , Adv. Funct. Mater. 2013, 23, 1774.

[95] S. Fu , X. Yi , Y. Li , Y. Li , X. Qu , P. Miao , Y. Xu , J. Hazard. Mater. 2024, 473, 134680. 38795486 10.1016/j.jhazmat.2024.134680

[96] J. H. Lu , H. Q. Zhao , Z. J. Wang , X. Y. Lin , W. M. Pi , X. Zhang , L. P. Yang , S. C. Yao , Y. Z. Zhang , X. M. Huang , H. M. Lei , P. L. Wang , Adv. Funct. Mater. 2024, 34, 2314089.

[97] J. Duan , Z. Chen , X. Liang , Y. Chen , H. Li , X. Tian , M. Zhang , X. Wang , H. Sun , D. Kong , Y. Li , J. Yang , Biomaterials 2020, 255, 120199. 32580099 10.1016/j.biomaterials.2020.120199

[98] H. Liang , J. Li , Y. He , W. Xu , S. Liu , Y. Li , Y. Chen , B. Li , ACS Biomater. Sci. Eng. 2016, 2, 317. 33429535 10.1021/acsbiomaterials.5b00363

[99] Q. Dai , H. Geng , Q. Yu , J. Hao , J. Cui , Theranostics 2019, 9, 3170. 31244948 10.7150/thno.31847 PMC6567970

[100] H. Geng , Q.‐Z. Zhong , J. Li , Z. Lin , J. Cui , F. Caruso , J. Hao , Chem. Rev. 2022, 122, 11432. 35537069 10.1021/acs.chemrev.1c01042

[101] A. Ali , R. Javed , S. Farhangi , T. Shah , S. Ullah , N. ul Ain , T. Liu , Z. Guo , I. Lynch , F. Raza , P. Zhang , Y. Rui , J. Drug Deliv. Sci. Technol. 2023, 84, 104536.

[102] H. Zhou , Q. P. Qian , Q. Z. Chen , T. Chen , C. Y. Wu , L. J. Chen , Z. G. Zhang , O. Q. Wu , Y. X. Jin , X. Z. Wang , Z. Y. Guo , J. Sun , J. Zhang , S. Y. Shen , X. Y. Wang , M. Jones , M. A. Khan , P. Makvandi , Y. L. Zhou , A. M. Wu , Small 2023, 20, 2308167.

[103] K. Li , G. Xiao , J. J. Richardson , B. L. Tardy , H. Ejima , W. Huang , J. L. Guo , X. P. Liao , B. Shi , Adv. Sci. 2019, 6, 1801688. 10.1002/advs.201801688 PMC6402403 30886799

[104] X. R. Song , S. X. Yu , G. X. Jin , X. Y. Wang , J. Z. Chen , J. Li , G. Liu , H. H. Yang , Small 2016, 12, 1506. 26763187 10.1002/smll.201503250

[105] a) W. F. Zeng , Z. M. Li , Q. L. Huang , C. D. Ding , L. Yang , W. Y. Wang , Z. Q. Shi , Y. Yang , H. Z. Chen , L. Mei , X. W. Zeng , Adv. Funct. Mater. 2024, 34, 2307241;

[106] S. Liu , Y. Feng , Q. Meng , X. Wang , Y. Liang , G. Yang , Y. Su , K. Zhang , C. Qi , K. Cai , X. Lei , Chem. Eng. J. 2024, 503, 158108.

[107] Y. X. Guo , F. G. Wu , Matter 2023, 6, 23.

[108] O. Mazaheri , M. S. Alivand , A. Zavabeti , S. Spoljaric , S. J. Pan , D. L. Chen , F. Caruso , H. C. Suter , K. A. Mumford , Adv. Funct. Mater. 2022, 32, 2111942.

[109] H. Luo , F. Wu , X. Wang , S. Lin , M. Zhang , Z. Cao , J. Liu , Mater. Today 2023, 62, 98.

[110] C. Shan , X. Cui , Z. Gao , M. Li , X. Zhang , M. Ashokkumar , A. Song , J. Cui , ACS Appl. Mater. Interfaces 2024, 16, 27988. 38748900 10.1021/acsami.4c05824

[111] T. Z. Wang , Z. X. Lin , O. Mazaheri , J. Q. Chen , W. J. Xu , S. J. Pan , C. J. Kim , J. J. Zhou , J. J. Richardson , F. Caruso , Angew. Chem. Int. Ed 2024, 63, 202410043. 10.1002/anie.202410043 38922736

[112] Z. Dong , Y. Hao , Q. Li , Z. Yang , Y. Zhu , Z. Liu , L. Feng , Nano Res. 2020, 13, 3057.

[113] J. X. Fan , D. W. Zheng , W. W. Mei , S. Chen , S. Y. Chen , S. X. Cheng , X. Z. Zhang , Small 2017, 13, 1702714. 10.1002/smll.201702714 29125688

[114] J. Y. Song , C. Cortez‐Jugo , S. J. Shirbin , Z. X. Lin , S. J. Pan , G. G. Qiao , F. Caruso , Adv. Funct. Mater. 2022, 32, 2107341.

[115] a) S. J. Pan , R. Guo , N. Bertleff‐Zieschang , S. S. Li , Q. A. Besford , Q. Z. Zhong , G. Yun , Y. T. Zhang , F. Cavalieri , Y. Ju , E. Goudeli , J. J. Richardson , F. Caruso , Angew. Chem. Int. Ed 2020, 59, 275; 10.1002/anie.201912296 31646700

[116] H. Ejima , J. J. Richardson , K. Liang , J. P. Best , M. P. van Koeverden , G. K. Such , J. Cui , F. Caruso , Science 2013, 341, 154. 23846899 10.1126/science.1237265

[117] S. P. Jingqu Chen , J. Zhou , Q.‐Z. Zhong , Y. Qu , J. J. Richardson , F. Caruso , Chem. Mater 2020, 32, 6975.

[118] Y. Ping , J. L. Guo , H. Ejima , X. Chen , J. J. Richardson , H. L. Sun , F. Caruso , Small 2015, 11, 2032. 25556334 10.1002/smll.201403343

[119] W. J. Xu , S. J. Pan , B. B. Noble , Z. X. Lin , S. K. Bhangu , C. J. Kim , J. Q. Chen , Y. Y. Han , I. Yarovsky , F. Caruso , Angew. Chem. Int. Ed. 2023, 62, 202302448. 10.1002/anie.202302448 PMC10947570 36872291

[120] S. Mao , Y. Ren , S. Chen , D. Liu , X. Ye , J. Tian , Carbohydr. Polym. 2023, 320, 121234. 37659819 10.1016/j.carbpol.2023.121234

[121] C. Zhou , Y. Zou , R. Xu , X. Han , Z. Xiang , H. Guo , X. Li , J. Liang , X. Zhang , Y. Fan , Y. Sun , Mater. Horiz 2023, 10, 3114. 37218586 10.1039/d3mh00033h

[122] Z. Zhang , Z. Gao , Y. Wang , L. Guo , C. Yin , X. Zhang , J. Hao , G. Zhang , L. Chen , Macromolecules 2019, 52, 2531.

[123] X. Zhang , L. Chen , C. Zhang , L. Liao , ACS Appl. Mater. Interfaces 2021, 13, 18175. 33826289 10.1021/acsami.1c03999

[124] H. J. Zhang , J. P. Zhang , B. Liu , J. Xiao , M. A. C. Stuart , G. H. Hou , H. R. Zhang , S. Liang , Z. K. Li , Q. M. Wang , S. N. Chen , P. L. Li , X. Li , Y. Li , Adv. Funct. Mater. 2024, 34, 2401064.

[125] a) T. Ozdal , E. Capanoglu , F. Altay , Food Res. Int. 2013, 51, 954;

[126] R. Lu , B. Zhao , L. Yang , S. Zheng , X. Zan , N. Li , ACS Appl. Mater. Interfaces 2023, 15, 20551. 37052959 10.1021/acsami.3c02047

[127] a) C. Chung , T. Rojanasasithara , W. Mutilangi , D. J. McClements , Food Res. Int. 2015, 76, 761; 28455061 10.1016/j.foodres.2015.07.003

[128] M. Shin , H. A. Lee , M. Lee , Y. Shin , J. J. Song , S. W. Kang , D. H. Nam , E. J. Jeon , M. Cho , M. Do , S. Park , M. S. Lee , J. H. Jang , S. W. Cho , K. S. Kim , H. Lee , Nat. Biomed. Eng. 2018, 2, 304. 30936449 10.1038/s41551-018-0227-9

[129] Y. Zou , X. Yang , E. Scholten , Food Hydrocolloid 2019, 89, 163.

[130] X. Chen , B. Zhou , J. Gao , D. Wu , H. Liang , Colloids Surf. A 2022, 652, 129879.

[131] Y. Han , R. P. M. Lafleur , J. Zhou , W. Xu , Z. Lin , J. J. Richardson , F. Caruso , J. Am. Chem. Soc. 2022, 144, 12510. 35775928 10.1021/jacs.2c05052

[132] H. Liang , B. Zhou , J. Li , X. Liu , Z. Deng , B. Li , J. Agric. Food Chem. 2018, 66, 6897. 29877704 10.1021/acs.jafc.8b01208

[133] K. Y. Huo , W. J. Liu , Z. Y. Shou , H. X. Wang , H. Liu , Y. Chen , X. J. Zan , Q. Wang , N. Li , Adv. Sci. 2024, 12, 2412194. 10.1002/advs.202412194 PMC11744643 39587774

[134] D. Chen , S. Stone , J. Ilavsky , O. Campanella , Food Hydrocolloid. 2024, 151, 109827.

[135] M. L. Picchio , M. S. Orellano , M. A. Motta , C. Huck‐Iriart , D. Sánchez‐deAlcázar , R. López‐Domene , B. Martín‐García , A. Larrañaga , A. Beloqui , D. Mecerreyes , M. Calderón , Adv. Funct. Mater. 2024, 34, 2313747.

[136] W. Zhang , J. Yi , X. Hu , M. Du , C. Guo , Food Hydrocolloid. 2025, 160, 110845.

[137] A. Singh , A. S. Jagtap , K. Rajpurohit , K. S. Singh , Carbohydr. Polym. 2025, 352, 123222. 39843117 10.1016/j.carbpol.2025.123222

[138] a) P. A. R. Fernandes , C. L. Bourvellec , C. Renard , D. F. Wessel , S. M. Cardoso , M. A. Coimbra , Carbohydr. Polym. 2020, 230, 115644; 31887907 10.1016/j.carbpol.2019.115644

[139] M. E. Vuillemin , F. Michaux , A. A. Adam , M. Linder , L. Muniglia , J. Jasniewski , Food Hydrocolloid. 2020, 107, 105919.

[140] D. Mozaffarian , Nature Food 2020, 1, 38.

[141] Y. Zheng , S. Chen , Y. Hu , X. Ye , S. Wang , J. Tian , Food Hydrocolloid. 2024, 157, 110361.

[142] S. Liu , F. Meng , R. Sun , Y. Li , H. Li , B. Liu , Carbohydr. Polym. 2025, 351, 123061. 39778992 10.1016/j.carbpol.2024.123061

[143] F. Li , X. Zhang , X. Liu , J. Zhang , D. Zang , X. Zhang , M. Shao , Int. J. Biol. Macromol. 2024, 271, 132444. 38797300 10.1016/j.ijbiomac.2024.132444

[144] J. Koh , Z. Xu , L. Wicker , Food Hydrocolloid. 2020, 99, 105354.

[145] A. Fernandes , J. Oliveira , F. Fonseca , F. Ferreira‐da‐Silva , N. Mateus , J. P. Vincken , V. de Freitas , Food Hydrocolloids 2020, 102, 105625.

[146] K. S. S. Hlaing , M. Fall , N. A. Tristanto , N. V. D. Carole , V. C. Kaharso , H. Golshany , M. Siddiquy , D. Yu , X. Yanshun , J. Qixing , W. Xia , Int. J. Biol. Macromol. 2025, 310, 143351. 40274145 10.1016/j.ijbiomac.2025.143351

[147] a) H. Jin , G. Sun , Q. Tang , S. Wang , S. Liu , Q. Cheng , L. Wang , Y. Li , Carbohydr. Polym. 2025, 354, 123306; 39978916 10.1016/j.carbpol.2025.123306

[148] M. Tudorache , N. Bordenave , Food Hydrocolloid. 2019, 97, 105193.

[149] X. Lin , H. Chen , L. Huang , S. Liu , C. Cai , Y. Li , S. Li , Int. J. Biol. Macromol. 2025, 291, 139043. 39710027 10.1016/j.ijbiomac.2024.139043

[150] X. Lin , L. Zhang , B. Duan , Mater. Horiz. 2021, 8, 2503. 34870294 10.1039/d1mh00878a

[151] D. Massari , M. Sgarzi , M. Gigli , C. Crestini , Adv. Sustain. Syst. 2024, 8, 2400389.

[152] D. Yang , L. Yang , P. Wang , ACS Materials Au 2023, 3, 83. 38089727 10.1021/acsmaterialsau.2c00056 PMC9999474

[153] M. Zhao , R. Wang , K. Yang , Y. Jiang , Y. Peng , Y. Li , Z. Zhang , J. Ding , S. Shi , Acta Pharm. Sin. B 2023, 13, 916. 36970219 10.1016/j.apsb.2022.10.019 PMC10031267

[154] Y. J. Qu , R. De Rose , C. J. Kim , J. J. Zhou , Z. X. Lin , Y. Ju , S. K. Bhangu , C. Cortez‐Jugo , F. Cavalieri , F. Caruso , Angew. Chem. Int. Ed 2023, 62, 202214935. 10.1002/anie.202214935 PMC10946467 36700351

[155] M. Zhang , X. Qin , Y. Gao , J. L. Liang , D. X. Xiao , X. L. Zhang , M. Zhou , Y. F. Lin , Adv. Sci. 2023, 10, 2303706. 10.1002/advs.202303706 PMC10667853 37797168

[156] G. Zhu , L. Mei , H. D. Vishwasrao , O. Jacobson , Z. Wang , Y. Liu , B. C. Yung , X. Fu , A. Jin , G. Niu , Q. Wang , F. Zhang , H. Shroff , X. Chen , Nat. Commun. 2017, 8, 1482. 29133898 10.1038/s41467-017-01386-7 PMC5684198

[157] a) X. Chen , W. Lan , J. Xie , Food Chem. 2024, 440, 138198; 38128429 10.1016/j.foodchem.2023.138198

[158] a) N. N. Zhang , R. F. Lin , W. Y. Gao , H. L. Xu , Y. J. Li , X. H. Huang , Y. C. Wang , X. H. Jing , W. X. Meng , Q. Xie , Adv. Sci. 2025, 12, 2405263; 10.1002/advs.202405263 PMC12005744 39921492

[159] R. Huang , W. Sun , W. Li , R. Hu , R. Meng , Z. Peng , R. Yang , T. Huang , J. Du , L. Shang , C. Xie , Chem. Eng. J. 2024, 500, 156869.

[160] J. Ziegler , P. J. Facchini , Annu. Rev. Plant Biol. 2008, 59, 735. 18251710 10.1146/annurev.arplant.59.032607.092730

[161] W. E. Bao , X. W. Liu , Y. L. Lv , G. H. Lu , F. Li , F. Zhang , B. Liu , D. Li , W. Wei , Y. Li , ACS Nano 2019, 13, 260. 30616348 10.1021/acsnano.8b05602

[162] Y. Xu , Z. Chen , W. Hao , Z. Yang , M. Farag , C. T. Vong , Y. Wang , S. Wang , J. Nanobiotechnol. 2024, 22, 538. 10.1186/s12951-024-02804-x PMC11373475 39227962

[163] W. Jiang , Z. Wu , Z. Gao , M. Wan , M. Zhou , C. Mao , J. Shen , ACS Nano 2022, 16, 15705. 36226996 10.1021/acsnano.2c06104

[164] C. Zhao , J. Li , S. Wang , Z. Xu , X. Wang , X. Liu , L. Wang , X. Huang , ACS Nano 2021, 15, 10048. 34047543 10.1021/acsnano.1c01694

[165] B. You , C. H. Chen , ACS Nano 2024, 18, 33545. 39615041 10.1021/acsnano.4c11349

[166] L. Jiang , Y. Zeng , H. Li , Z. Lin , H. Liu , J. J. Richardson , Z. Gao , D. Wu , L. Liu , F. Caruso , J. Zhou , J. Am. Chem. Soc. 2023, 145, 24108. 37788442 10.1021/jacs.3c07748

[167] X. Z. Lian , Y. Y. Huang , Y. Y. Zhu , Y. Fang , R. Zhao , E. Joseph , J. L. Li , J. P. Pellois , H. C. Zhou , Angew. Chem. Int. Ed. 2018, 57, 5725. 10.1002/anie.201801378 PMC6621563 29536600

[168] X. Cai , L. Jiao , H. Yan , Y. Wu , W. Gu , D. Du , Y. Lin , C. Zhu , Mater. Today 2021, 44, 211.

[169] S. Shi , Y. Han , J. Feng , J. Shi , X. Liu , B. Fu , J. Wang , W. Zhang , J. Duan , Redox Biol. 2024, 73, 103217. 38820984 10.1016/j.redox.2024.103217 PMC11177078

[170] A. Bigham , V. Rahimkhoei , P. Abasian , M. Delfi , J. Naderi , M. Ghomi , F. Dabbagh Moghaddam , T. Waqar , Y. Nuri Ertas , S. Sharifi , N. Rabiee , S. Ersoy , A. Maleki , E. Nazarzadeh Zare , E. Sharifi , E. Jabbari , P. Makvandi , A. Akbari , Chem. Eng. J. 2022, 432, 134146.

[171] B. Mirzaei , A. Zarrabi , A. Noorbakhsh , A. Amini , P. Makvandi , RSC Adv. 2021, 11, 7862. 35423323 10.1039/d0ra10701h PMC8695096

[172] J. Zhao , H. Wang , X. Song , Y. Sun , X. Zhang , J. Zheng , R. Hu , Nano Energy 2024, 126, 109687.

[173] F. C. Lin , W. S. Yang , B. L. Lu , Y. L. Xu , J. P. Chen , X. X. Zheng , S. Y. Liu , C. S. Lin , H. B. Zeng , B. Huang , Adv. Funct. Mater. 2025, 35, 2416419.

[174] J. Zheng , J. He , J. Wu , Y. Yu , Y. Fu , S. Yin , K. Li , Y. Li , L. Cai , Y. Du , X. Lu , C. Xie , ACS Nano 2025, 19, 17796. 40310951 10.1021/acsnano.5c03256

[175] E. Xie , X. Zhang , Y. Zhou , Y. Yang , Y. Lin , Y. Niu , J. Wei , D. Li , Adv. Fiber Mater. 2025, 7, 296.

[176] Q. Ding , Y. Wang , T. Wang , C. Zhang , S. Yang , L. Mao , Y. Cheng , Y. Li , K. Lin , Bioact. Mater. 2025, 43, 550. 40115875 10.1016/j.bioactmat.2024.09.037 PMC11923377

[177] M. A. Rahim , S. L. Kristufek , S. Pan , J. J. Richardson , F. Caruso , Angew. Chem., Int. Ed. 2018, 58, 1904. 10.1002/anie.201807804 30221440

[178] Z. Liu , T. Wang , L. Zhang , Y. Luo , J. Zhao , Y. Chen , Y. Wang , W. Cao , X. Zhao , B. Lu , F. Chen , Z. Zhou , L. Zheng , Adv. Healthcare Mater. 2024, 13, 2304158. 10.1002/adhm.202304158 38319101

[179] G. Gong , Q. Yin , Q. Liu , Y. Zhang , J. Liu , M. Dai , J. Li , X. He , J. Pan , Y. He , B. Wang , J. Tong , J. Guo , Small 2024, 21, 2408746. 10.1002/smll.202408746 39610194

[180] H. Qiu , Q. Tu , P. Gao , X. Li , M. F. Maitz , K. Xiong , N. Huang , Z. Yang , Biomaterials 2021, 269, 120626. 33418199 10.1016/j.biomaterials.2020.120626

[181] a) R. Dong , B. Guo , Nano Today 2021, 41, 101290;

[182] K. Wen , C. Zhang , G. H. Zhang , M. L. Wang , G. K. Mei , Z. Z. Zhang , W. Q. Zhao , W. J. Guo , Q. Zhou , E. Z. Liu , Y. T. Zhu , J. Bai , M. F. Zhu , W. Wang , Z. F. Liu , X. Zhou , Adv. Mater. 2024, 36, 2314158. 10.1002/adma.202314158 39081084

[183] W. Liu , W. Ou‐Yang , C. Zhang , Q. Wang , X. Pan , P. Huang , C. Zhang , Y. Li , D. Kong , W. Wang , ACS Nano 2020, 14, 12905. 32946218 10.1021/acsnano.0c03855

[184] N. Liu , S. Zhu , Y. Deng , M. Xie , M. Zhao , T. Sun , C. Yu , Y. Zhong , R. Guo , K. Cheng , D. Chang , P. Zhu , Bioact. Mater. 2023, 24, 69. 36582352 10.1016/j.bioactmat.2022.12.009 PMC9772805

[185] H. T. Li , J. H. Zhang , H. R. Xue , L. Li , X. Liu , L. Yang , Z. P. Gu , Y. Y. Cheng , Y. W. Li , Q. Huang , Mater. Horiz. 2023, 10, 1789. 36853277 10.1039/d3mh00005b

[186] H. J. Zhang , J. H. Zhang , T. Y. Wang , H. T. Li , R. Zhang , X. C. Chen , Z. P. Gu , Y. W. Li , Adv. Funct. Mater. 2024, 34, 2408462.

[187] T. Chen , L. Wan , Y. Xiao , K. Wang , P. Wu , C. Li , C. Huang , X. Liu , W. Xue , G. Sun , X. Ji , H. Lin , Z. Ji , J. Nanobiotechnol. 2024, 22, 653. 10.1186/s12951-024-02916-4 PMC11515499 39443923

[188] Q. Huang , Q. Shan , F. T. Ma , S. L. Li , P. Sun , Int. J. Biol. Macromol. 2025, 301, 140133. 39842566 10.1016/j.ijbiomac.2025.140133

[189] S. Y. Wei , Z. Y. Shou , D. Yang , L. X. Sun , Y. Guo , Y. Wang , X. J. Zan , L. X. Li , C. W. Zhang , Adv. Sci. 2024, 11, 2407425. 10.1002/advs.202407425 PMC11672291 39556697

[190] J. H. Zhang , J. Zhong , B. Liang , W. J. Liu , H. J. Zhang , T. Y. Wang , L. P. Huang , L. Yang , Z. P. Gu , Y. W. Li , Adv. Funct. Mater. 2025, 10.1002/adfm.202500271.

[191] W. Fu , Z. Huang , W. Li , L. Xu , M. Yang , Y. Ma , H. Liu , H. Qian , W. Wang , Bioact. Mater. 2025, 46, 118. 39760067 10.1016/j.bioactmat.2024.12.004 PMC11697280

[192] D. Wu , B. Zhou , J. Li , X. Y. Wang , B. Li , H. S. Liang , Adv. Funct. Mater. 2022, 11, 2200559.

[193] a) S. Y. Wang , H. Q. Jing , R. Yang , Z. Heger , S. Krizkova , Y. Zhou , X. Y. Liang , V. Adam , N. Li , Adv. Funct. Mater. 2024, 34, 2314194;

[194] P. Sun , Z. Li , D. Zhang , W. Zeng , Y. Zheng , L. Mei , H. Chen , N. Gao , X. Zeng , Chin. Chem. Lett. 2024, 35, 108346.

[195] a) C. Cheng , W. X. Jiang , Y. L. Luo , L. Wan , X. Guo , Z. Y. Xie , R. Tang , T. Huang , J. X. Wang , C. R. Du , Z. G. Wang , H. T. Ran , P. Li , Z. Y. Zhou , J. L. Ren , Small 2023, 19, 2206174; 10.1002/smll.202206174 36651135

[196] a) J. Xie , L. Zhang , Y.‐Y. Bai , W.‐J. Wang , X. Hu , S. Li , Y. Tian , Food Chem. 2025, 467, 142287; 39637665 10.1016/j.foodchem.2024.142287

[197] S. K. Barik , S. Sengupta , R. Arya , S. Kumar , J. J. Kim , R. Chaurasia , Adv. Nutr. 2025, 16, 100346. 39566886 10.1016/j.advnut.2024.100346 PMC11697556

[198] G. Yun , J. J. Richardson , M. Capelli , Y. J. Hu , Q. A. Besford , A. C. G. Weiss , H. Lee , I. S. Choi , B. C. Gibson , P. Reineck , F. Caruso , Adv. Funct. Mater. 2020, 30, 1905805.

[199] Z. X. Lin , J. J. Zhou , Y. J. Qu , S. J. Pan , Y. Y. Han , R. P. M. Lafleur , J. Q. Chen , C. Cortez‐Jugo , J. J. Richardson , F. Caruso , Angew. Chem., Int. Ed. 2021, 60, 24968. 10.1002/anie.202108671 34528750

[200] Y. Wu , S.‐F. Torabi , R. J. Lake , S. Hong , Z. Yu , P. Wu , Z. Yang , K. Nelson , W. Guo , G. T. Pawel , J. Van Stappen , X. Shao , L. M. Mirica , Y. Lu , Sci. Adv. 2023, 9, ade7622. 10.1126/sciadv.ade7622 PMC10115418 37075105

[201] a) H. M. Geng , Z. W. Li , Z. Li , Y. Q. Zhang , Z. L. Gao , L. Sun , X. A. Li , J. W. Cui , S. L. Ni , J. C. Hao , Proc. Natl. Acad. Sci. USA 2023, 120, 2220300120; 10.1073/pnas.2220300120 PMC10655560 37948584

[202] Y. Liu , D. Zhao , F. Yang , C. Ye , Z. Chen , Y. Chen , X. Yu , J. Xie , Y. Dou , J. Chang , ACS Nano 2024, 18, 7890. 38445977 10.1021/acsnano.3c09286

[203] M. Efenberger‐Szmechtyk , N. Agnieszka , A. Czyzowska , Crit. Rev. Food Sci. Nutr. 2021, 61, 149. 32043360 10.1080/10408398.2020.1722060

[204] A. Pizzi , Sustain. Chem. Pharm. 2021, 22, 100481.

[205] P. P. Fan , S. Y. Zhang , Y. Q. Wang , T. Li , H. H. Zhang , P. K. Zhang , S. Huang , Nat. Commun. 2024, 15, 1970. 38443335 10.1038/s41467-024-45543-1 PMC10915175

